# The Origin and Early Evolution of Sauria: Reassessing the Permian Saurian Fossil Record and the Timing of the Crocodile-Lizard Divergence

**DOI:** 10.1371/journal.pone.0089165

**Published:** 2014-02-27

**Authors:** Martín D. Ezcurra, Torsten M. Scheyer, Richard J. Butler

**Affiliations:** 1 School of Geography, Earth and Environmental Sciences, University of Birmingham, Edgbaston, Birmingham, United Kingdom; 2 GeoBio-Center, Ludwig-Maximilian-Universität München, Munich, Germany; 3 Paläontologisches Institut und Museum, Universität Zürich, Zurich, Switzerland; Institute of Biochemistry and Biology, Germany

## Abstract

Sauria is the crown-group of Diapsida and is subdivided into Lepidosauromorpha and Archosauromorpha, comprising a high percentage of the diversity of living and fossil tetrapods. The split between lepidosauromorphs and archosauromorphs (the crocodile-lizard, or bird-lizard, divergence) is considered one of the key calibration points for molecular analyses of tetrapod phylogeny. Saurians have a very rich Mesozoic and Cenozoic fossil record, but their late Paleozoic (Permian) record is problematic. Several Permian specimens have been referred to Sauria, but the phylogenetic affinity of some of these records remains questionable. We reexamine and review all of these specimens here, providing new data on early saurian evolution including osteohistology, and present a new morphological phylogenetic dataset. We support previous studies that find that no valid Permian record for Lepidosauromorpha, and we also reject some of the previous referrals of Permian specimens to Archosauromorpha. The most informative Permian archosauromorph is *Protorosaurus speneri* from the middle Late Permian of Western Europe. A historically problematic specimen from the Late Permian of Tanzania is redescribed and reidentified as a new genus and species of basal archosauromorph: *Aenigmastropheus parringtoni*. The supposed protorosaur *Eorasaurus olsoni* from the Late Permian of Russia is recovered among Archosauriformes and may be the oldest known member of the group but the phylogenetic support for this position is low. The assignment of *Archosaurus rossicus* from the latest Permian of Russia to the archosauromorph clade Proterosuchidae is supported. Our revision suggests a minimum fossil calibration date for the crocodile-lizard split of 254.7 Ma. The occurrences of basal archosauromorphs in the northern (30°N) and southern (55°S) parts of Pangea imply a wider paleobiogeographic distribution for the group during the Late Permian than previously appreciated. Early archosauromorph growth strategies appear to be more diverse than previously suggested based on new data on the osteohistology of *Aenigmastropheus*.

## Introduction

Saurians, or crown group diapsids, are highly taxonomically and morphologically diverse in extant ecosystems, with around 9,400 lepidosaur (snakes, lizards and rhynchocephalians) and 10,000 archosaur (birds and crocodilians) species, including cursorial, semi-aquatic, marine, fossorial and volant forms [1], [2]. The stem-groups of Lepidosauria (non-lepidosaurian Lepidosauromorpha) and Archosauria (non-archosaurian Archosauromorpha) also include several morphologically disparate saurian lineages that were mostly restricted in time to the Triassic. These lineages formed important components of Triassic continental assemblages, and include kuehneosaurids, rhynchosaurs, proterosuchids, erythrosuchids, euparkeriids, doswelliids and proterochampsids [3]–[8]. However, the earliest (i.e. pre-Mesozoic) evolutionary history of Sauria is poorly known and there has been substantial debate regarding the late Paleozoic (i.e. Permian) record of the group (e.g. [3], [9]–[18]).

The best source of information on the early history of Sauria comes from the numerous fossils of the well-known basal archosauromorph *Protorosaurus speneri* from the Late Permian of Germany and England [19]–[21]. Multiple less completely known specimens have been also argued to be Permian members of Sauria (e.g. Parrington's “problematic reptile” from Tanzania, UMZC T836 [9]). A better understanding of the Permian saurian record is fundamental for providing more accurate fossil constraints on the calibration of the crocodile-lizard ( = bird-lizard) divergence, a major split within vertebrates that is of keen interest to molecular and evolutionary biologists and vertebrate paleontologists alike [22]–[25]. A better knowledge of Permian saurians is also necessary to improve understanding of phylogenetic relationships within early members of Diapsida, an area of key interest because of the controversial systematic affinities of several possible saurian lineages including turtles, choristoderans and sauropterygians (e.g. [26]–[39]). New information on the Permian saurian record may also yield fresh insights into survivorship of this clade across the Permian-Triassic mass extinction and the dynamics of the dramatic saurian radiation in post-extinction ecosystems.

Here, we revisit and reexamine the Permian record of Sauria to provide new information on the diversity, phylogeny, morphology, geographic distribution and physiology of Permian members of the clade, and the timing of the crocodile-lizard (or bird-lizard) split. We fully or partially redescribe some Permian saurian specimens (e.g. UMZC T836; BP/1/4220; *Eorasaurus olsoni*) and we erect a new genus and species of archosauromorph, *Aenigmastropheus parringtoni*, for a specimen from the middle Late Permian of Tanzania. Our new data provides an improved understanding of early saurian and early archosauromorph evolutionary history, including calibration dates for molecular biology.

## Materials and Methods

### Access to specimens

The type and referred specimens of *Eorasaurus olsoni* (PIN 156/108–111), the new genus and species *Aenigmastropheus parringtoni* (UMZC T836), and *Archosaurus rossicus* (PIN 1100/55), as well as BP/1/4220 and all specimens that are used here for comparative purposes (indicated by the citation of their taxonomic name and respective collection accession numbers at relevant points in the manuscript) were studied at first-hand, with the explicit permission of appropriate curators and/or collection managers (see Acknowledgments), in recognized, scientifically accessible collections. Repository locations and abbreviations for all specimens discussed in the text and abbreviations listed in the Acknowledgments are listed below. No specimens were purchased or donated for the purpose of this study. The holotype of the new taxon *Aenigmastropheus parringtoni* was loaned from the collection of the UMZC with permission of the collection manager and returned before submission of the manuscript.

### Terminology

We follow here the nomenclature for vertebral laminae and fossae of Wilson [40] and Wilson et al. [41]. We also follow the terminology of Rewcastle [42] for limb orientation in sprawling animals.

### Histological analysis

The paleohistological sections of the holotype of the new taxon *Aenigmastropheus parringtoni* were prepared by one of us (TS) in the facilities of the PIMUZ using standard techniques. Paleohistological slices are reposited with the holotype specimen in the UMZC collections.

### Phylogenetic analysis

In order to test quantitatively the phylogenetic relationships of early saurians, including the new taxon *Aenigmastropheus parringtoni*, we modified the data matrix of Reisz et al. [43] (Appendix SI in File S1). This data matrix was employed by Reisz et al. [43] to test the position of the taxon *Apsisaurus witteri* among basal diapsids and early synapsids (“pelycosaurs”), demonstrating that *Apsisaurus* is a varanopid synapsid rather than a diapsid as originally identified. Similarly, we wished to test whether the new taxon *Aenigmastropheus* is a saurian diapsid, or belongs to some other group of early amniotes; thus, the scope and aims of the Reisz et al. [43] analysis broadly match those of this study.

The taxonomic sample of the data matrix of Reisz et al. [43] was enlarged with the addition of the new taxon *Aenigmastropheus parringtoni* and 13 early saurian species (*Archosaurus rossicus*, *Eorasaurus olsoni*, *Erythrosuchus africanus*, *Euparkeria capensis*, *Gephyrosaurus bridensis*, *Howesia browni*, *Macrocnemus bassanii*, *Mesosuchus browni*, *Noteosuchus colletti*, *Paliguana whitei*, *Proterosuchus fergusi*, *Protorosaurus speneri*, *Tanystropheus longobardicus*). *Eorasaurus olsoni* and *Noteosuchus colletti* are included for the first time in a quantitative phylogenetic analysis. The hypothesis of Dilkes [4] that *Noteosuchus colletti* is a junior synonym of *Mesosuchus browni* was not followed here because this proposal was based on generalized plesiomorphic similarities rather than autapomorphies and the temporal gap between the two species spans most of the Early Triassic [11]. As a result, *Noteosuchus colletti* was scored as an independent operational taxonomic unit (OTU) because of its potential to shed light on the minimal divergence time of Rhynchosauria. The derived rhynchosaur *Hyperodapedon* was pruned *a priori* from the data matrix because its advanced morphology is not congruent with that observed in basal members of the group (*Mesosuchus browni*, *Howesia browni*, *Noteosuchus colletti*) and the absence of species linking these basal forms and *Hyperodapedon* could cause artifacts in the optimization of characters within Archosauromorpha. *Trilophosaurus* was replaced with *Trilophosaurus buettneri* in order to avoid ambiguities in the scorings for the genus from the more poorly known *Trilophosaurus jacobsi* [44]. Several Triassic basal lepidosauromorphs were not included in this phylogenetic analysis because they come from a multi-taxic assemblage of amniote material [45], [46] and their hypodigms may represent more than one species (e.g. *Marmoretta*, *Sophineta*, *Pamelina*).

All the added OTUs were scored based on first hand observations, with the exception of *Gephyrosaurus bridensis*, which was scored following Evans [47], [48] (Table 1). Several scores for the non-saurian neodiapsids *Coelurosauravus* spp., *Youngina capensis*, and *Acerosodontosaurus piveteaui*, and the archosauromorphs *Prolacerta broomi* and *Trilophosaurus buettneri* were modified based on first hand observation of specimens or new published information (Appendix SII in File S1).

**Table 1:** Specimens or references employed here to score the 14 species added to the taxonomic sample of Reisz et al. [43].

*Archosaurus rossicus*	PIN 1100/55 (only the holotype premaxilla was considered); [88]
*Eorasaurus olsoni*	PIN 156/108, 109, 110, 111; [89]
*Erythrosuchus africanus*	AMNH 5596, BP/1/2096, 2529, 3893, 4526, 4645, 4680, 5207; GHG AK-82-22; NHMUK R525, R533, R2790, R3592, R3762 (scapula), R3764, NHMUK unnumbered, NM QR1473, SAM-PK-905, 912, 978, 1315, 3028, 3612, 7684, 11330, K1098, K1118, K10025, UMZC T666, T700; [113]
*Euparkeria capensis*	GPIT 1681, SAM-PK-5867, 5883, 6047, 6048, 6049, 6050, 7696, 7700, 7712, 7713, 10671, 13664, 13665, 13666, K8050, K8309, UMZC T692, T921; [192]
*Gephyrosaurus bridensis*	[47], [48]
*Howesia browni*	SAM-PK-5884, 5885, 5886; [118]
*Macrocnemus bassanii*	PIMUZ T2472, 4355, 4822; [225], [226]
*Mesosuchus browni*	SAM-PK-5882, 6046, 6536, 7416; [4]
*Noteosuchus colletti*	AM 3591 (holotype); [11]
*Paliguana whitei*	AM 3585 (holotype); [10]
*Proterosuchus fergusi*	BP/1/3993, 4016, 4224; BSPG 1934-VIII-514; GHG 231; RC 59; SAM-PK-591 (holotype), 11208, K140, K10603, TM 201; [90], [227]
*Protorosaurus speneri*	BSPG 1995 I 5 (cast of WMSN P47361), AS VII 1207; NHMW 1943I4 (lectotype); SMNS 55387 (cast of Simon/Bartholomäus specimen); USNM 442453 (cast of NMK S 180); ZMR MB R2171-3; [21]
*Tanystropheus longobardicus*	PIMUZ T2189, 2793, 2817, 2818, 3901; SMNS 54147, 54626, 54628, 54630–54632, 54634, 55341, 56289, 59380, 84821: [111], [228]

The character list was enlarged with the addition of 107 characters from Dilkes [4], Müller [32] and Senter [49], plus some new characters (Appendix SI in File S1). As a result, the new data matrix includes 40 taxa and 219 characters (Appendices SI and SIII in File S1).

The data matrix was analyzed under equally-weighted parsimony using TNT 1.1 [50]. A heuristic search of 1,000 replications of Wagner trees (with random addition sequence) followed by the tree bisection and reconnection (TBR) branch-swapping algorithm (holding 10 trees per replicate) was performed. The best tree(s) obtained at the end of the replicates were subjected to a final round of TBR branch swapping. Zero length branches among any of the recovered most parsimonious trees (MPTs) were collapsed (rule 1 of Coddington and Scharff [51]). Characters 2, 6, 8, 10, 12, 16–18, 20, 21, 24, 26, 30, 31, 36–38, 40, 45, 49, 51–55, 57, 58, 62, 70, 73, 79, 91, 92, 94, 103, 143, 160, 198, 214, 216 and 219 represent nested sets of homologies and/or entail presence and absence information and as a result they were treated as additive (ordered).

As measures of tree support, decay indices ( = Bremer support) were calculated and a bootstrap resampling analysis, with 10,000 pseudoreplicates, was performed. We report both absolute and GC (i.e. difference between the frequency that the original group and the most frequent contradictory group are recovered in the pseudoreplicates) frequencies. Taxa with high amounts of missing data may reduce node support values not as a result of a real low robustness of the node but because of ambiguous optimizations generated by unknown character states. Accordingly, a second round of decay indices and bootstrap resampling was conducted following the *a posteriori* pruning of saurian OTUs with large amount of missing data (i.e. *Aenigmastropheus parringtoni*, *Archosaurus rossicus*, *Eorasaurus olsoni*, *Noteosuchus colletti*, *Paliguana whitei*).

### Nomenclatural Acts

The electronic edition of this article conforms to the requirements of the amended International Code of Zoological Nomenclature, and hence the new names contained herein are available under that Code from the electronic edition of this article. This published work and the nomenclatural acts it contains have been registered in ZooBank, the online registration system for the ICZN. The ZooBank LSIDs (Life Science Identifiers) can be resolved and the associated information viewed through any standard web browser by appending the LSID to the prefix “http://zoobank.org/”. The LSID for this publication is: urn:lsid:zoobank.org:pub:A899B6F4-7362-44FB-88BD-188E8C642E0D. The electronic edition of this work was published in a journal with an ISSN, and has been archived and is available from the following digital repositories: PubMed Central and LOCKSS.

### Institutional abbreviations

AM, Albany Museum, Grahamstown, South Africa; AMNH, American Museum of Natural History, New York, USA; BP, Evolutionary Studies Institute (formerly Bernard Price Institute for Palaeontological Research), University of the Witswatersrand, Johannesburg, South Africa; BSPG, Bayerische Staatssammlung für Paläontologie und Geologie, Munich, Germany; FC-DPV, Colección de Vertebrados Fósiles, Departamento de Paleontología, Facultad de Ciencias, Universidad de la República, Montevideo, Uruguay; FMNH, Field Museum of Natural History, Chicago, USA; GHG, Geological Survey, Pretoria, South Africa; GPIT, Paläontologische Sammlung der Universität Tübingen, Tübingen, Germany; IVPP, Institute of Vertebrate Paleontology and Paleoanthropology, Beijing, China; MB, Museum für Naturkunde – Leibniz-Institut für Evolutions- und Biodiversitätsforschung, Berlin, Germany; MCNAM, Museo de Ciencias Naturales y Antropológicas de Mendoza (J. C. Moyano), Mendoza, Argentina; MCP, Museu de Ciências e Tecnologia da Pontifícia Universidade Católica do Rio Grande do Sul, Porto Alegre, Brazil; MCZ, Museum of Comparative Zoology, Cambridge, USA; MNHN, Muséum national d'Histoire naturelle, Paris, France; NHMUK, The Natural History Museum, London, UK; NHMW, Naturhistorisches Museum Wien, Vienna, Austria; NM, National Museum, Bloemfontein, South Africa; PIMUZ, Paläontologisches Institut und Museum der Universität Zürich, Zurich, Switzerland; PIN, Paleontological Institute of the Russian Academy of Sciences, Moscow, Russia; PVL, Paleontología de Vertebrados, Instituto ‘Miguel Lillo’, San Miguel de Tucumán, Argentina; PVSJ, División de Paleontología de Vertebrados del Museo de Ciencias Naturales y Universidad Nacional de San Juan, San Juan, Argentina; SAM, Iziko South African Museum, Cape Town, South Africa; SMNS, Staatliches Museum für Naturkunde Stuttgart, Stuttgart, Germany; SSWG, Sektion Geologie, Ernst-Moritz-Arndt Universität, Greifswald, Germany; TM, Ditsong National Museum of Natural History (formerly Transvaal Museum), Pretoria, South Africa; TMM, Texas Memorial Museum, Austin, USA; UMZC, University Museum of Zoology, Cambridge, UK; USNM, National Museum of Natural History (formerly United States National Museum), Smithsonian Institution, Washington, D.C., USA; UTGD, School of Earth Sciences, University of Tasmania, Hobart, Australia; WMSN, Westfälisches Museum für Naturkunde, Münster, Germany; ZAR, Muséum national d'Histoire naturelle (Zarzaitine collection), Paris, France.

## Results

### Phylogenetic analysis

**Figure 1:**
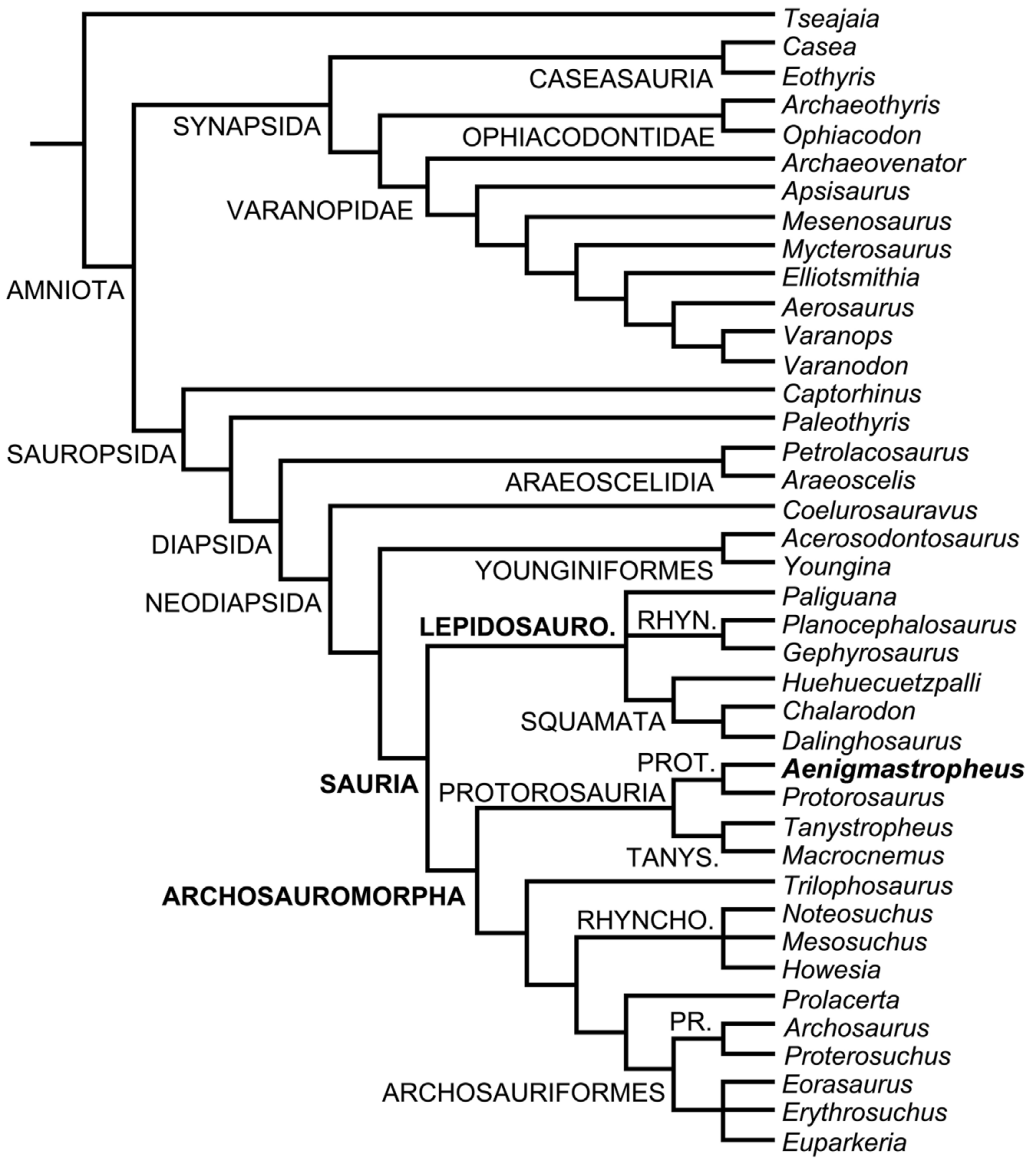
Phylogenetic relationships of *Aenigmastropheus parringtoni* and other basal archosauromorphs. Single most parsimonious tree recovered here with zero length branches collapsed. Abbreviations: Lepidosauro., Lepidosauromorpha; Pr., Proterosuchidae; Prot., Protorosauridae; Rhyn., Rhynchocephalia; Rhyncho., Rhynchosauria; Tanys., Tanystropheidae.

The phylogenetic analysis recovered one MPT of 861 steps (Fig. 1; Appendix SIV in File S1), with a consistency index (CI) of 0.337, a retention index (RI) of 0.645, and the best score hit 320 times out of the 1000 replications. The topology of the tree is completely congruent with that obtained by Reisz et al. [43] and only differs in the resolution of the polytomies recovered by Reisz et al. [43] within Squamata and Varanopidae. As a result, only the relationships within Sauria, which includes the species that we have added to the phylogenetic dataset, will be described here.

**Figure 2:**
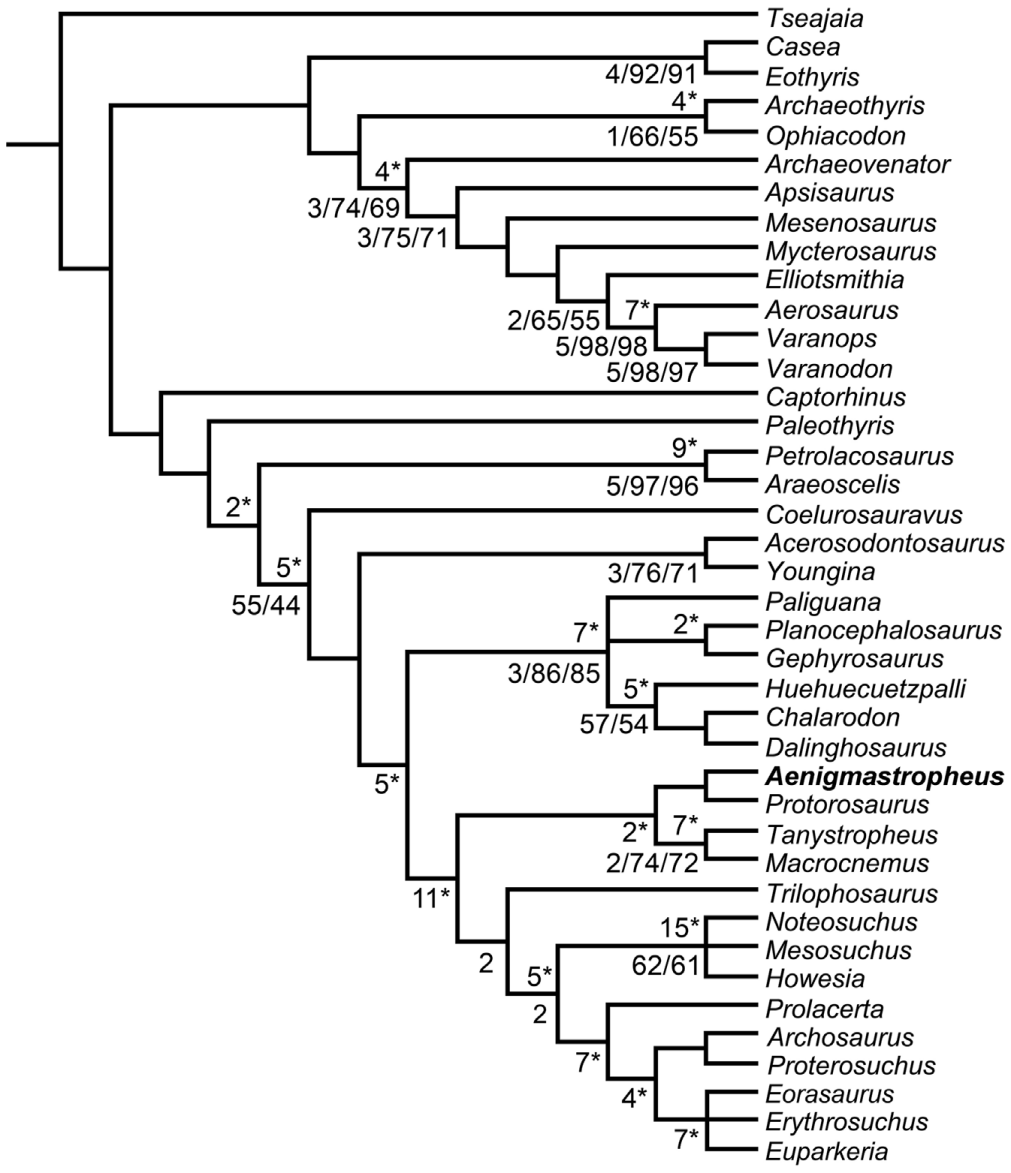
Bremer support and bootstrap resampling frequencies for the phylogenetic hypothesis presented here. Numbers below the nodes are Bremer support, absolute bootstrap and GC bootstrap resampling frequencies, respectively, for the single most parsimonious tree recovered here. Numbers above the nodes and with an asterisk are Bremer support values after *a posteriori* pruning of fragmentary saurian terminals (see text).

The new taxon *Aenigmastropheus parringtoni* was recovered as a basal archosauromorph, being nested within Protorosauria as the sister-taxon of *Protorosaurus speneri* from the Late Permian of Europe. The decay indices for Archosauromorpha, Protorosauria and Protorosauridae (i.e. the *Protorosaurus*+*Aenigmastropheus* clade) were minimal (i.e. 1) and the bootstrap resampling frequencies below 50% (although the absolute bootstrap support of Archosauromorpha is 49%) (Fig. 2). When *Aenigmastropheus* and other saurians with high amounts of missing data (i.e. *Paliguana*, *Noteosuchus*, *Eorasaurus*, *Archosaurus*) were *a posteriori* pruned, the new decay index for Archosauromorpha was 11 and the absolute and GC bootstrap resampling frequencies rose to 97% and 96%, respectively.

The Middle Triassic taxa *Tanystropheus longobardicus* and *Macrocnemus bassanii* were also found as members of Protorosauria, being sister-taxa and comprising the clade Tanystropheidae (sensu Dilkes [4]). Following the pruning of fragmentary saurians, the decay index for Proterosauria increased to 2 but the bootstrap resampling frequencies remained lower than 50%. Support values were moderately high for Tanystropheidae, with a decay index of 2 and absolute and GC bootstrap resampling frequencies of 74% and 72%, respectively. Following the pruning of fragmentary saurians the decay index for Tanystropheidae rose to 7 and the resampling frequencies rose to 93% and 91%, respectively. Protorosauria was recovered as the most basal clade within Archosauromorpha, being the sister group of a clade composed of *Trilophosaurus buettneri* and more derived archosauromorphs (Rhynchosauria, *Prolacerta broomi* and Archosauriformes). The decay index for the latter clade was 2 and absolute and GC bootstrap resampling frequencies remained below 50% even when fragmentary saurians were pruned.

The recovery of *Trilophosaurus buettneri* as the sister-taxon of the Rhynchosauria+*Prolacerta*+Archosauriformes clade agrees with several previous hypotheses (e.g. [4], [52]–[54]) but contrasts with some studies that recovered a *Trilophosaurus*+Rhynchosauria clade (e.g. [21], [55]) or a position of *Trilophosaurus* as closer to archosauriforms than to rhynchosaurs (e.g. [56], [57]). The decay index of the Rhynchosauria+*Prolacerta*+Archosauriformes clade was 2 and the absolute and GC bootstrap resampling frequencies were below 50%. Following the *a posteriori* pruning of poorly known saurians the decay index rose to 5 and the bootstrap frequencies rose to 72% and 64%, respectively.

*Howesia browni*, *Mesosuchus browni* and *Noteosuchus colletti* were found within a monophyletic Rhynchosauria (sensu Dilkes [4]), but relationships among these three taxa were unresolved. The decay index was minimal, but absolute and GC bootstrap resampling frequencies were 72% and 64%. This was the first quantitative analysis to include *Noteosuchus colletti* and the results support its identification as the oldest known rhynchosaur [4], [11], [58]. Following the pruning of fragmentary saurian (including *Noteosuchus*) the decay index of Rhynchosauria rose to 15 and the resampling frequencies both rose to 99%.

*Prolacerta broomi* was found as the sister-taxon of Archosauriformes. As a result, Prolacertiformes sensu Benton [52] was polyphyletic, as also recovered by several other recent analyses [21], [53], [54]. The decay index for the *Prolacerta*+Archosauriformes clade was minimal and the absolute and GC bootstrap resampling frequencies were below 50%, but following the pruning of fragmentary saurians the decay index rose to 7 and the resampling frequencies rose to 80% and 76%, respectively.

Two clades were found within Archosauriformes, one composed of the proterosuchids *Proterosuchus fergusi* and *Archosaurus rossicus* and the other consisting of a trichotomy including the Late Permian *Eorasaurus olsoni* and the early Middle Triassic *Euparkeria capensis* and *Erythrosuchus africanus*. This topology is consistent with the interrelationships recently recovered for the clade by other analyses [59] (see below for a discussion of the phylogenetic position of *Eorasaurus olsoni*). Decay indices for Archosauriformes and clades nested within Archosauriformes were minimal and absolute and GC bootstrap resampling frequencies were below 50%, although the *Proterosuchus*+*Archosaurus* clade had bootstrap resampling frequencies of 47% and 43%, respectively. However, following the pruning of fragmentary saurians the decay index of Archosauriformes rose to 4 and the bootstrap resampling frequencies rose to 82% and 73%, respectively, and the decay index of the *Erythrosuchus+Euparkeria* clade rose to 7 and the bootstrap resampling frequencies to 93% and 90%, respectively.

Lepidosauromorpha was composed by three lineages: *Paliguana whitei*, Rhynchocephalia (*Gephyrosaurus bridensis* and *Planocephalosaurus robinsonae*) and Squamata (*Dalinghosaurus longidigitus*, *Huehuecuetzpalli mixtecus* and *Chalarodon madagascariensis*), a result congruent with that of Evans and Borsuk-Białynicka [45]. The relationships between these three lineages were unresolved. Support values for Lepidosauromorpha were relatively high, with a decay index of 3 and absolute and GC bootstrap resampling frequencies of 86% and 85%, respectively. The decay index of Lepidosauromorpha rose to 7 and the bootstrap resampling frequencies increased to 94% and 90%, respectively, following the pruning of fragmentary saurians. Support values were low for Rhynchocephalia (absolute and GC bootstrap resampling frequencies of 48% and 35%). Following the pruning of fragmentary saurians, the decay index of Rhynchocephalia increased to 2 and the absolute and GC bootstrap resampling frequencies increased to 76% and 62%. The decay index of Squamata was minimal and the absolute and GC bootstrap resampling frequencies were 57% and 54%. Following the pruning of fragmentary taxa the decay index rose to 5 and the bootstrap resampling frequencies increased to 90% and 85%, respectively.

### Review of the Permian saurian record

Sauria comprises Lepidosauromorpha, Archosauromorpha, their most recent common ancestor, and all their extinct descendants [57]. Several footprints and ichnotaxa potentially attributable to both lepidosauromorphs (e.g. *Ganasauripus ladinus*, *Paradoxichnium radeinensis*) and archosauromorphs (e.g. *Protochirotherium* isp., *Synaptichnium* isp.) have been described from Upper Permian beds of southern Europe [60], [61] and northern Africa [62]. However, here we focus solely on the body fossil record of Permian saurians.

#### “Younginiformes”

**Figure 3:**
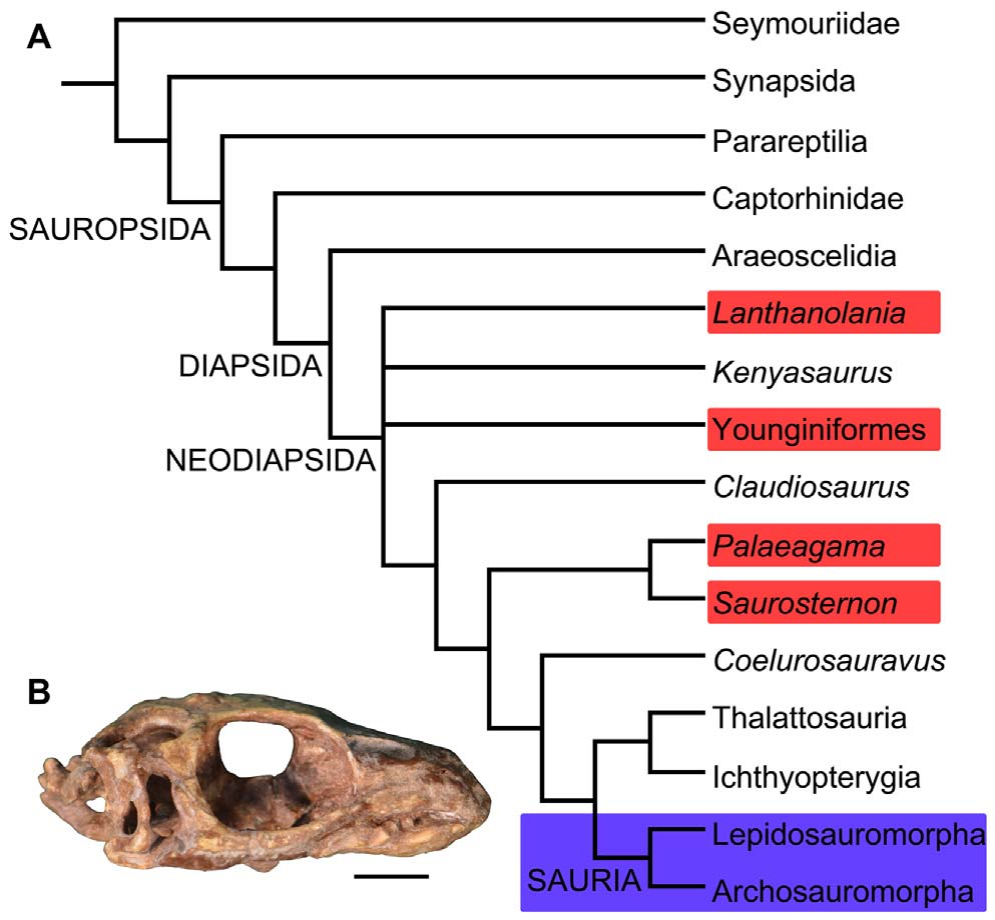
Simplified phylogenetic relationships of Diapsida. (A) Phylogenetic positions of Sauria (blue box) and several species previously considered as Permo-Triassic lepidosauromorphs (red boxes), based upon the phylogenetic analysis of Bickelmann et al. [70] (illustration simplified) (B) Holotype (AM 3585) of *Paliguana whitei*, the oldest known lepidosauromorph from the Early Triassic of South Africa, in right lateral view. Scale bar equals 5 mm.

Several Late Permian diapsid species (all referred to at various points as “eosuchians”) from South Africa (*Youngina capensis* [63], [64]), Tanzania (*Tangasaurus mennelli* [65], [66]) and Madagascar (*Hovasaurus boulei* [67], [68]; *Acerosodontosaurus piveteaui* [69], [70]; *Thadeosaurus colcanapi* [71]) have been considered by some authors to form a monophyletic Younginiformes [52], [56], [66] within Lepidosauromorpha [52], [56], [57], [72]. However, subsequent work has suggested that “Younginiformes” form a paraphyletic assemblage [70], and these species are now widely accepted as basal non-saurian neodiapsids and therefore not lepidosauromorphs (e.g. [4], [13], [32], [43], [49], [70], [73]–[75]) (Fig. 3A). “Younginiformes” do not, therefore, represent Permian saurians.

#### Other putative Permian lepidosauromorph records

Five basal diapsid species (all referred to at various points as “eolacertilians”) have been proposed as possible Permian lepidosauromorphs, and have often been identified as lizards: *Saurosternon bainii*, *Palaeagama vielhaueri*, *Paliguana whitei* and *Lacertulus bipes* from South Africa [10], [76]–[80], the former three species forming the “Paliguanidae” of Carroll [10], and *Lanthanolania ivakhnenkoi* from the Middle Permian of Russia [15].

Among the South African specimens, the exact stratigraphic position of the type and only known specimen of *Saurosternon bainii* is poorly constrained [76], but it is of definite Late Permian age [10]. By contrast, the type and only specimen of *Palaeagama vielhaueri* [79] cannot be stratigraphically constrained with certainty beyond a Late Permian–Early Triassic age, although an Early Triassic age may be more likely [10]. The stratigraphic position of the type and only specimen of *Lacertulus bipes* is not constrained beyond Permian–Triassic [80]. Whereas Carroll [10] considered *Paliguana whitei* [77] to be of uncertain Late Permian–Triassic age, Kitching [81] and Groenwald & Kitching [82] listed this species as derived from the *Lystrosaurus* Assemblage Zone of Early Triassic age (Induan–?Olenekian [83]–[85]).

Several of these species are of uncertain phylogenetic position. *Saurosternon bainii* and *Palaeagama vielhaueri* were assigned to Lepidosauromorpha by Gauthier et al. [57], whereas Evans [3] considered *Saurosternon bainii* as a possible basal lepidosauromorph and *Palaeagama vielhaueri* as an indeterminate diapsid. However, more recent quantitative phylogenetic analyses identified both species as non-saurian basal diapsids [18], possibly forming a monophyletic clade [32], [70] (Fig. 3A). These results do not therefore support the positions of *Saurosternon bainii* and *Palaeagama vielhaueri* within Sauria. *Lacertulus bipes* is not a squamate, but its phylogenetic relationships cannot be further determined because of the poor preservation of the specimen [3], [86].

*Paliguana whitei* (Fig. 3B) possesses a quadrate conch ([17], [45], [87]; AM 201, MDE pers. obs.), a character widely accepted as a diagnostic feature of Lepidosauromorpha [57]. As a result, Evans and Borsuk-Białynicka [45] and Evans and Jones [17] considered *Paliguana whitei* as referable to Lepidosauromorpha. In agreement with this hypothesis, our phylogenetic results recovered *Paliguana whitei* as a basal lepidosauromorph. However, as discussed above, *Paliguana whitei* is currently considered Early Triassic in age. Therefore, although *Paliguana whitei* is accepted as one of the oldest known lepidosauromorphs [17] it does not represent a Permian record of the group.

Finally, in the original description of the species *Lanthanolania ivakhnenkoi* from the Middle Permian of Russia, Modesto and Reisz [15] recovered this species as a lepidosauromorph in some of the most parsimonious trees and as the sister-taxon of Sauria in others. These results suggested possible but uncertain saurian affinities. However, a more recent phylogenetic analysis recovered *Lanthanolania ivakhnenkoi* close to the base of Neodiapsida [75] and outside of Sauria, thus contradicting the possible inclusion of this species within Lepidosauromorpha or Sauria.

In summary, there is currently no Permian specimen that can unambiguously assigned to Lepidosauromorpha. The earliest known member of Lepidosauromorpha (*Paliguana whitei*) comes from lowermost Triassic (Induan–?Olenekian) rocks that were deposited in the aftermath of the Permo-Triassic mass extinction.

#### Permian records of Archosauromorpha

Only three Permian species are currently considered as unambiguous members of Archosauromorpha: *Protorosaurus speneri* [19] from Germany and England, and *Archosaurus rossicus* [88] and *Eorasaurus olsoni* [89], both from Russia. In addition to these species, several incomplete specimens of Permian or possible Permian age have been considered as possible members of Archosauromorpha. This material includes a “problematic reptile” from Tanzania [9], an isolated cervical vertebra from South Africa [90], and some vertebral material from Uruguay [91]. We discuss these three unambiguous Permian archosauromorphs and the additional possible records of the clade in more detail below.

“*Acanthotoposaurus bremneri*”, based upon a single specimen (SAM-PK-K6888) from the Late Permian of South Africa, has been also considered an early member of Archosauromorpha [92]. However, Reisz et al. [14] provided a strong rebuttal to this interpretation and considered “*Acanthotoposaurus bremneri*” to be a subjective junior synonym of the “younginiform” *Youngina capensis* and, as a result, a non-saurian diapsid (see above). Another South African Late Permian taxon, *Heleosaurus scholtzi*, was suggested as a possible archosaur ancestor by Carroll [93]. However, *Heleosaurus* has never been formally referred to Archosauromorpha, and Reisz & Modesto [16] reinterpreted *Heleosaurus* as a varanopid synapsid, and thus non-diapsid. *Mesenosaurus romeri* from the Permian of Russia was reinterpreted by Ivachnenko [12] as the oldest known archosaur, rather than a “pelycosaurian” synapsid as described by previous authors [94], [95]. However, additional specimens and new anatomical work have demonstrated that *Mesenosaurus romeri* is a varanopid synapsid [43], [96].

#### *Protorosaurus speneri*

**Figure 4:**
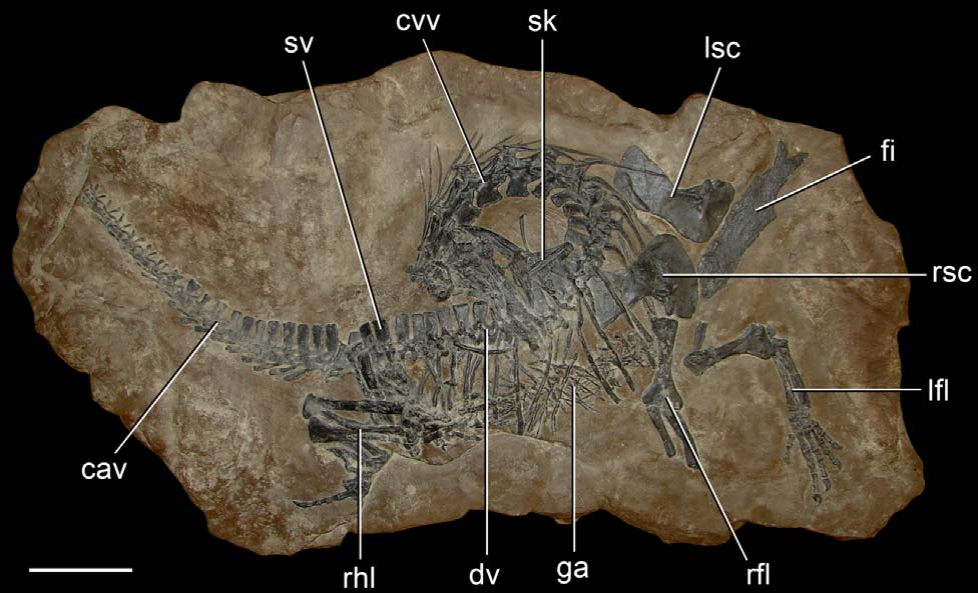
*Protorosaurus speneri*, a protorosaurian archosauromorph from the middle Late Permian of Western Europe. Axial skeleton primarily exposed in right lateral view (BSPG 1995 I 5, cast of WMSN P47361) collected near Münster, Germany. Abbreviations: cav, caudal vertebrae; cvv, cervical vertebrae; dv, dorsal vertebrae; fi, fisch; ga, gastralia; lfl, left forelimb; lsc, left scapula and coracoid; rfl, right forelimb; rhl, right hindlimb; rsc, right scapula and coracoid; sk, skull; sv, sacral vertebrae. Scale bar equals 10 cm.

*Protorosaurus speneri* [19] was a quadrupedal archosauromorph reaching a body length of 1.5–2 meters [21], known from numerous specimens from the Upper Permian Kupferschiefer Formation of Germany and England (Fig. 4). The first fossil specimen of *Protorosaurus speneri* was discovered in Germany in 1706, and Spener [97] published a description of this specimen (identifying it as the remains of a Nile crocodile), making the taxon one of the first fossil reptiles ever described. Meyer [19], [98], [99] identified the remains as of a previously unknown extinct reptile, erected the new species *Protorosaurus speneri*, and published a monographic description. Subsequently, *Protorosaurus* remains were also recovered from England [20], and Gottman-Quesada & Sander [21] recently published a full monographic redescription of *Protorosaurus speneri*, based on the abundant German material.

At least 28 *Protorosaurus speneri* specimens are known from the states of Thuringia and Hesse in central Germany. All of these German specimens come from the Kupferschiefer, part of the classic Permian Zechstein Group, which is divided into six cycles (Z1–Z6; e.g. [100]). The Kupferschiefer forms part of the basal cycle of the Zechstein (Z1) and is a dark bituminous and calcareous shale deposited in a marine environment. The Kupferschiefer is often given as Tatarian (equivalent to the Wordian–Wuchiapingian) (e.g. [21]) or Capitanian (e.g. [24]) in age. Brauns et al. [101] reported a date of 257.3±2.6 Ma (Late Permian/Lopingian: Wuchiapingian) for the Kupferschiefer based on a Re-Os geochronological study. The presence of the conodont *Mesogondolella britannica* in Kupferschiefer equivalents supports a middle Wuchiapingian age for the *Protorosaurus*-bearing levels ([102], [103]; Schneider pers. comm. 2012). *Protorosaurus* specimens from northwest England have been discovered in the Marl Slate [20], considered a lateral equivalent of the Kupferschiefer on the basis of independent geological data. A putative second species of *Protorosaurus* from England, *P. huxleyi* [104], was referred to the genus *Adelosaurus* by Evans [56] and considered as a probable diapsid of uncertain affinities.

The phylogenetic position of *Protorosaurus speneri* within Archosauromorpha has been widely accepted and is uncontroversial [4], [21], [52], [56], [72]–[74], [105], [106] and supported by our phylogenetic results. *Protorosaurus speneri* has generally been considered to belong to a clade of otherwise Triassic archosauromorphs referred to either as Prolacertiformes or Protorosauria, although the composition and monophyly of this grouping is debated (see summary in Gottman-Quesada & Sander [21]). Our phylogenetic results support referral of this species to Protorosauria, and suggest that “Prolacertiformes” is polyphyletic.

Sues & Munk [107] briefly mentioned archosauromorph cranial and postcranial remains from fissure fill deposits at Korbach, in Hesse, central Germany, including “a *Protorosaurus*-like form and tooth-bearing jaw fragments of a large, as yet unidentifiable taxon”. The formation and infilling of this fissure was inferred to have taken place during the Z2 cycle of the Zechstein, indicating that these archosauromorph remains are slightly younger than *Protorosaurus speneri*. Unfortunately, more detailed descriptions of this material have not yet been published.

#### *Eorasaurus olsoni*

**Figure 5:**
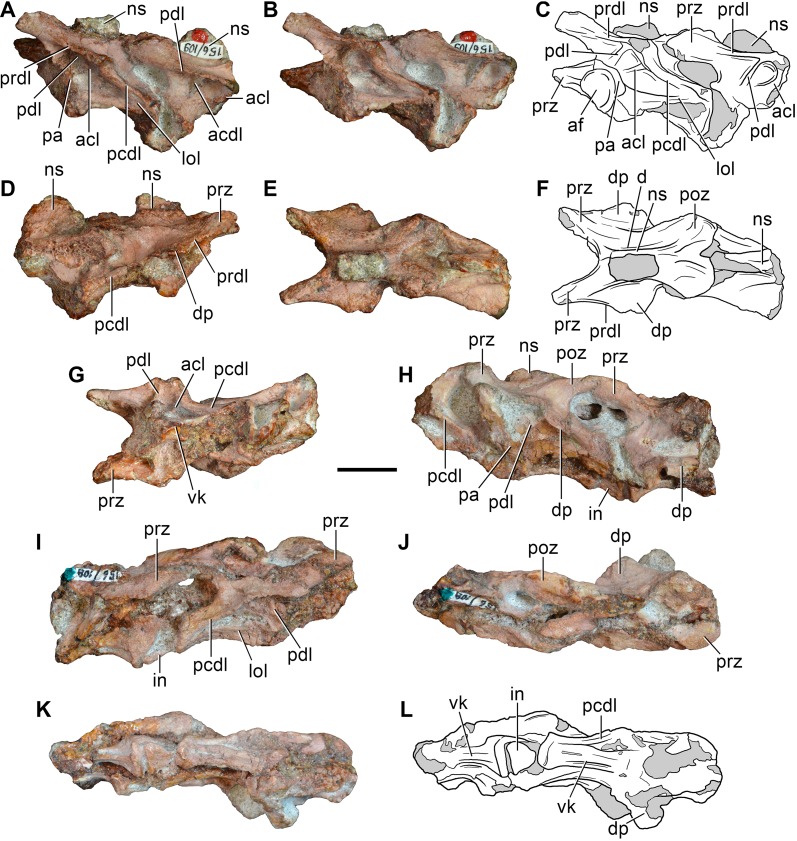
*Eorasaurus olsoni*, a possible early archosauriform from the late Middle–early Late Permian of Russia. Middle (PIN 156/109: A–G) and posterior (PIN 156/108, holotype: H–L) cervical vertebrae in left lateral (A, H), left ventrolateral (B, C), right lateral (D, I), dorsal (E, F, J), and ventral (G, K, L) views. Abbreviations: acl, accessory lamina; af, anterior articular facet; d, depression; dp, diapophysis; in, intercentrum; lol, longitudinal lamina; ns, neural spine; pa, parapophysis; pcdl, posterior centrodiapophyseal lamina; pdl, paradiapophyseal lamina; poz, postzygapophysis; prdl, prezygodiapophyseal lamina; prz, prezygapophysis; vk, ventral keel. Scale bar equals 1 cm.

**Figure 6:**
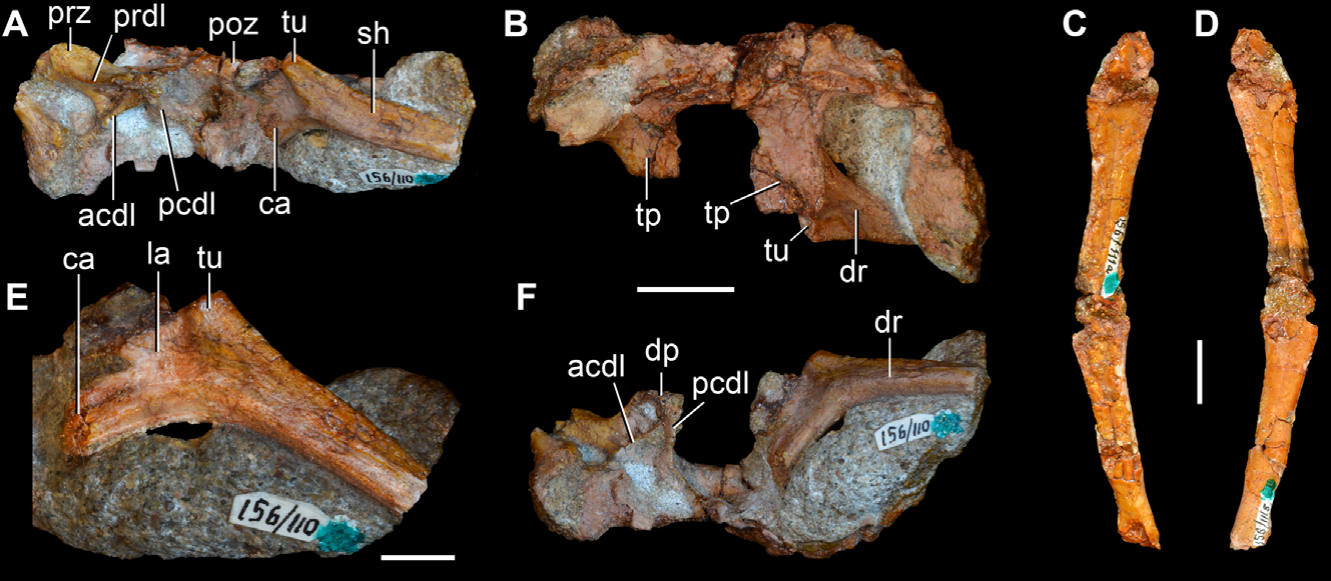
*Eorasaurus olsoni*, a possible early archosauriform from the late Middle–early Late Permian of Russia. Anterior dorsal vertebrae and rib in articulation (PIN 156/110: A, B, F), close-up of the anterior dorsal rib (E), and probable long bones (PIN 156/111a, b: C, D) in left lateral (A, E), dorsal (B), and ventral (F) views. Abbreviations: acdl, anterior centrodiapophyseal lamina; ca, capitulum; dp, diapophysis; dr, anterior dorsal rib; la, lamina; pcdl, posterior centrodiapophyseal lamina; prdl, prezygodiapophyseal lamina; poz, postzygapophysis; prz, prezygapophysis; sh, shaft; tp, transverse process; tu, tuberculum. Scale bars equal 1 cm in (A–D, F) and 5 mm in (E).

Sennikov [89] erected *Eorasaurus olsoni* based on a sequence of cervico-dorsal vertebrae with one dorsal rib in articulation and some additional bone fragments (Figs. 5, 6). This material was collected from the bank of the Volga River in Tatarstan in European Russia during the 1930s. *Eorasaurus olsoni* comes from the upper substage of the Severodvinian regional stage [89], [108]. Recent magnetostratigraphic evidence suggests that the base of the Severodvinian stage is within the Capitanian (approximately middle Capitanian), but there exists uncertainty regarding the age of the upper boundary of the Severodvinian, which may be close to the Wuchiapingian–Changhsingian boundary ([109]: fig. 8) or to the Capitanian–Wuchiapingian boundary ([109]: p. 46). Accordingly, *Eorasaurus olsoni* is late Capitanian–Wuchiapingian in age and, as a result, roughly contemporaneous with (or possibly slightly older than) the middle Wuchiapingian *Protorosaurus speneri*.

Sennikov [89] considered *Eorasaurus olsoni* to be closely related to *Protorosaurus speneri* and considered both taxa to be members of Protorosauridae. *Eorasaurus olsoni* was diagnosed by Sennikov on the basis of a combination of characters of the cervico-dorsal axial skeleton, such as moderately elongated and strongly parallelogram-shaped vertebral centra, well-developed ridges situated below the diapophyses, absence of intercentra and three-headed anterior dorsal ribs ([89]: p. 95). Despite the importance of *Eorasaurus olsoni* as one of the oldest known archosauromorphs, this taxon has been largely ignored by subsequent authors. Sennikov [89] provided a detailed description and drawings of *Eorasaurus olsoni*, and, as a result, a full redescription is not necessary here. However, we complement the original description of the species with some additional observations and provide a few reinterpretations based upon first hand examination of the specimens.

We agree with Sennikov [89] in considering the holotype (PIN 156/109) and referred specimens (PIN 156/108, 110, 111) of *Eorasaurus olsoni* to belong to a single individual, because they possess the same mode of preservation and are congruent in size and morphology. The preserved bones of *Eorasaurus olsoni* are generally well preserved, but there are several broken areas and damaged surfaces. The vertebrae of *Eorasaurus olsoni* probably represent a continuous series of nine postaxial vertebrae, including middle (PIN 156/109; Fig. 5A–G) and posterior (PIN 156/108; Fig. 5H–L) cervical vertebrae and anterior dorsal vertebrae (PIN 156/110; Fig. 6A, B, E, F) (Table 2). The vertebrae of PIN 156/108 are interpreted as belonging to the posterior cervical series because the parapophyses are situated on the dorsal halves of the centra and the vertebrae of PIN 156/109 are identified as middle cervicals because the parapophyses are situated at mid-height on the anterior margins of the centra (Fig. 5: pa). By contrast, Sennikov [89] originally interpreted the holotype vertebrae of PIN 156/109 as posterior cervical vertebrae and those of PIN 156/108 as more anterior cervicals. No traces of neurocentral sutures were observed in the vertebrae of *Eorasaurus olsoni*, suggesting that the specimen was not a juvenile when it died [110].

**Table 2:** Measurements of the preserved bones of *Eorasaurus olsoni* (PIN 156/108, 109, 110) in millimeters.

**PIN 156/108 (most anterior almost complete vertebra)**			
	Centrum length	18.3	
	Anterior articular facet height	(8.4)	
	Anterior articular facet width	ca. 6.4	
	Posterior articular facet width	10.2	
	Maximum height of the vertebra	(21.8)	
	Length along zygapophyses	28.8	
	Length of base of neural spine	(11.4)	
	Intercentrum length	4.7	
	Intercentrum width	5.7	
**PIN 156/109 (most anterior almost complete vertebra)**			
	Centrum length	16.9	
	Anterior articular facet height	10.0	
	Anterior articular facet width	9.2	
	Posterior articular facet height	10.4	
	Posterior articular facet width	ca. 9.8	
	Maximum height of the vertebra	(18.8)	
	Neural canal height	2.9	
	Neural canal width	6.7	
	Length along zygapophyses	28.2	
	Length of base of neural spine	11.0	
**PIN 156/110**	**Vertebra**	**A**	**B**
	Centrum length	16.5	-
	Anterior articular facet height	(8.4)	-
	Anterior articular facet width	(6.8)	-
	Maximum height of the vertebra	(17.4)	(11.0)
	Length along zygapophyses	(23.3)	ca. 21.2
	Transverse process width	(5.8)	11.5
	Transverse process length at distal end	-	7.6
	Neural spine length	(8.7)	(9.6)
**Anterior dorsal rib**			
	Length	(23.9)	
	Anteroposterior proximal depth	(15.8)	
	Length tubercular facet	3.6	
**PIN 156/111**	**Long bone**	**A**	**B**
	Length	(46.4)	(38.7)

Values between brackets indicate incomplete measurements and the value given is the maximum measurable. The length along the zygapophyses is the maximum anteroposterior length between the anterior tips of the prezygapophyses and the posterior tips of the postzygapophyses. In PIN 156/110 the vertebrae were labeled as A or B, where A corresponds to the most anterior element in the specimen. Maximum deviation of the digital caliper equals 0.02 mm but measurements were rounded to the nearest 0.1 millimeter.

The centra of the middle cervical vertebrae (PIN 156/109; Fig. 5A–G) possess low and well defined, median longitudinal ventral keels (Fig. 5: vk). The lateral surface of the centrum possesses a horizontal lamina that extends from the base of the parapophysis to the posterior margin of the centrum (Fig. 5: lol), resembling the condition of *Macrocnemus bassanii* (PIMUZ T4822) and *Tanystropheus longobardicus* (PIMUZ T2818). The lateral surface of the centrum immediately dorsal to the horizontal lamina is strongly concave, but the degree of concavity seems to be exaggerated due to the collapse of the cortical bone. The neural canal is considerably wider than tall in anterior view. Well-developed paradiapophyseal, posterior centrodiapophyseal and prezygodiapophyseal laminae extend away from the base of the diapophysis (Fig. 5: acdl, pcdl, pdl, prdl), as also occurs in the posterior cervical and/or dorsal vertebrae of the enigmatic neodiapsid *Helveticosaurus zollingeri* (PIMUZ T4352), numerous basal archosauromorphs (e.g. *Tanystropheus longobardicus* [111]: figs. 52–54; *Protorosaurus speneri*, BSPG 1995 I 5, cast of WMSN P47361; *Spinosuchus caseanus* [112]), and several basal archosauriforms and crown group archosaurs (e.g. *Erythrosuchus africanus*, NHMUK R3592, [113]; *Euparkeria capensis*, UMZC T921; *Bromsgroveia walkeri* [114]; *Hypselorhachis mirabilis* [115]; *Silesaurus opolensis* [116]; *Herrerasaurus ischigualastensis*, PVSJ 373, [117]). The posterior centrodiapophyseal lamina extends to the posterodorsal corner of the centrum and contacts in this area the horizontal lamina of the centrum.

The neural arch laminae delimit centrodiapophyseal and prezygapophyseal centrodiapophyseal fossae, but the postzygapophyseal centrodiapophyseal fossa is absent. The centrodiapophyseal fossa is subdivided by an accessory lamina that extends anteriorly from the posterior centrodiapophyseal lamina and contacts the base of the parapophysis and the paradiapophyseal lamina (Fig. 5: acl). This accessory lamina is not present in other basal diapsids and represents an autapomorphy of *Eorasaurus olsoni*. A ridge extends anteriorly from the base of the postzygapophysis onto the lateral surface of the neural arch, and curves ventrally, being positioned between the prezygapophysis and diapophysis but without reaching either of these structures. This ridge delimits the lateral margin of a shallow depression positioned next to the base of the neural spine (Fig. 5: d). A similar depression is also found in the cervical vertebrae of several basal archosauromorphs (e.g. *Protorosaurus speneri*, BSPG 1995 I 5; *Prolacerta broomi*, BP/1/2675; *Proterosuchus fergusi*, GHG 231). The base of the neural spine is transversely thin and not as wide as it appears in the drawing in the original description ([89]: fig. 1d). There is no evidence of intercentra in PIN 156/109 [74].

The posterior cervical vertebrae (PIN 156/108) (Fig. 5H–L) possess a morphology that is very similar to that found in the middle cervical vertebrae, including the presence of a ventral longitudinal keel and the same suite of laminae on the centrum and neural arch. The accessory lamina that divides the centrodiapophyseal fossa is even more extensively developed laterally in the posterior cervical vertebrae than the middle cervicals. The neural arches of the posterior cervicals of PIN 156/108 each possess an incipient postzygapophyseal centrodiapophyseal fossa, consistent with their more posterior position in the axial series than the middle cervical vertebrae of PIN 156/109. There is no depression lateral to the base of the neural spine. Two intercentra are present in PIN 156/108 but were overlooked in the original description of the specimen. The presence of intercentra resembles the condition observed in the postaxial cervical vertebrae of several basal archosauromorphs (e.g. *Macrocnemus bassanii*, PIMUZ T4822; *Trilophosaurus buettneri* [44]: fig. 30; *Proterosuchus fergusi*, NM QR 1484). By contrast, postaxial cervical intercentra are absent in *Tanystropheus longobardicus* (PIMUZ T2817), *Protorosaurus speneri* [21], *Mesosuchus browni* [4] and *Howesia browni* [118]. The intercentra are situated anterior to the most complete vertebrae of PIN 156/108 (Fig. 5: in). The intercentra are proportionally large and subtriangular in ventral view, with a transversely broad posterior margin and a tapering anterior end.

The general morphology of the anterior dorsal vertebrae (PIN 156/110) is congruent with that of the cervical vertebrae, but in the anterior dorsals the centrum is subrectangular in lateral view (Fig. 6A, B, E, F). The neural arches of the anterior dorsal vertebrae possess prezygodiapophyseal, posterior centrodiapophyseal and anterior centrodiapophyseal/paradiapophyseal laminae (Fig. 6: acdl, pcdl, prdl). It is not possible to determine whether or not the latter lamina reached the parapophysis because the relevant area is damaged. The centrodiapophyseal, prezygapophyseal centrodiapophyseal, and postzygapophyseal centrodiapophyseal fossae are present and the latter fossa is better developed than in the posterior cervical vertebrae (PIN 156/108). There is no accessory lamina subdividing the centrodiapophyseal fossa, contrasting with the condition in the cervical vertebrae (PIN 156/108, 109). The left transverse process of the third anterior dorsal vertebra of PIN 156/110 is complete and is very strongly developed laterally, with a transverse length to centrum length ratio of 0.70 (Fig. 6B: tp). This ratio resembles that observed in the anterior dorsal vertebrae of *Trilophosaurus buettneri* (0.84 [44]: fig. 37), *Proterosuchus fergusi* (0.95, NM QR 1484) and *Erythrosuchus africanus* (0.85, NHMUK R3592). By contrast, proportionally shorter transverse processes are present in the anterior dorsal vertebrae of *Youngina capensis* (0.46, BP/1/3859), early lepidosaurs (e.g. *Gephyrosaurus bridensis* [48]: figs. 5, 6; *Planocephalosaurus robinsonae*, 0.18–0.25 [119]: figs. 5, 6), protorosaurs (*Protorosaurus speneri*, 0.38–0.45, BSPG 1995 I 5; *Tanystropheus longobardicus*, 0.46, SMNS 54628), *Macrocnemus bassanii* (0.56, PIMUZ T2472), *Mesosuchus browni* (approximately 0.5 [4]: p. 513) and *Prolacerta broomi* (0.55, BP/1/2675). The transverse process of *Eorasaurus olsoni* is slightly anteroposteriorly compressed close to its base, but possesses an overall subrectangular outline in dorsal view. There is no depression on the neural arch lateral to the base of the neural spine, similar to the condition in the posterior cervical vertebrae (PIN 156/108) but contrasting with condition in the middle cervical vertebrae (PIN 156/109).

The proximal half of the left dorsal rib is preserved in near articulation with the third vertebra of PIN 156/110 (Fig. 6A, B, E, F). The capitulum of this anterior dorsal rib lacks its distal end, but the process is relatively long. The tuberculum is complete and is very short. The articular facet of the tuberculum is flat and oval, with an acute posterior margin. The tuberculum is not well differentiated from the rest of the rib due to the presence of a thin lamina of bone that connects it with the capitulum (Fig. 6E: la). The lamina extends up to the same level as the articular facet of the tuberculum. An apparent notch between the capitulum and the lamina is the result of breakage. There is no conclusive evidence for the presence of a third articular facet on the anterior dorsal rib (contra Sennikov [89]). Although the lamina between the capitulum and tuberculum resembles a similar lamina that houses the third articular facet in *Prolacerta broomi* (BP/1/2675), *Proterosuchus fergusi* (NM QR 1484) and *Erythrosuchus africanus* [113], the preserved portion of the lamina in PIN 156/110 lacks the transverse thickening that bears the facet in the those taxa.

PIN 156/111 is represented by two long bones (PIN 156/111a, 111b; Fig. 6C, D) and a small block of matrix with some unidentified partial bones (PIN 156/111). PIN 156/111a and 111b are interpreted as two limb bones in articulation. They do not seem to be rib shafts because they lack the curvature that would be expected for a rib and the proximal and distal ends of the bones are subequally expanded (contra Sennikov [89]). Neither bones appears to be a femur and, as a result, they may represent a humerus and an ulna or radius. The long bones are strongly flattened, resembling the condition of the forelimb bones of protorosaurian archosauromorphs (e.g. *Tanystropheus longobardicus* [111]). However, there are no clear features that would allow a confident identification of these bones; as a consequence, they are not very informative.

The morphology of *Eorasaurus olsoni* is congruent with that observed in basal archosauromorphs (e.g. presence of well developed prezygodiapophyseal and anterior and posterior centrodiapohyseal laminae, anterior dorsal zygapophyses close to the sagittal plane of the axial skeleton; cf. Sennikov [89]). Indeed, our phylogenetic results recovered *Eorasaurus olsoni* as a derived archosauromorph within Archosauriformes.

Our reexamination of the anatomy of *Eorasaurus olsoni* allowed us to reinterpret some characters that were included in the original diagnosis of the species, including the supposed absence of intercentra and the presence of a dorsal rib with three articular facets. Accordingly, we provide here an emended diagnosis for the species. *Eorasaurus olsoni* is a small archosauromorph that differs from other diapsids in possessing the following combination of characters: presence of prezygodiapophyseal and anterior and posterior centrodiapophyseal laminae; an accessory lamina that extends anteroventrally from the posterior centrodiapophyseal lamina and subdivides the centrodiapophyseal fossa (autapomorphic); strongly parallelogram-shaped middle and posterior cervical centra; and presence of postaxial cervical intercentra.

#### *Archosaurus rossicus*

Tatarinov [88] described *Archosaurus rossicus* on the basis of fragmentary cranial and postcranial remains from the upper substage of the Vyatskian regional stage at the Vyazniki locality in Vladamir region, Russia. *Archosaurus rossicus* is considered to belong to the uppermost part of the Tatarian series (e.g. [120], [121]), and part of the “Vyazniki Biotic Assemblage” which also includes pareiasaurs, chroniosuchians, therocephalians, and dicynodonts [122]. The late Tatarian and Vyazniki Biotic Assemblage have in recent years been generally correlated to the late Changhsingian, or terminal Permian [84], [122]–[125].

**Figure 7:**
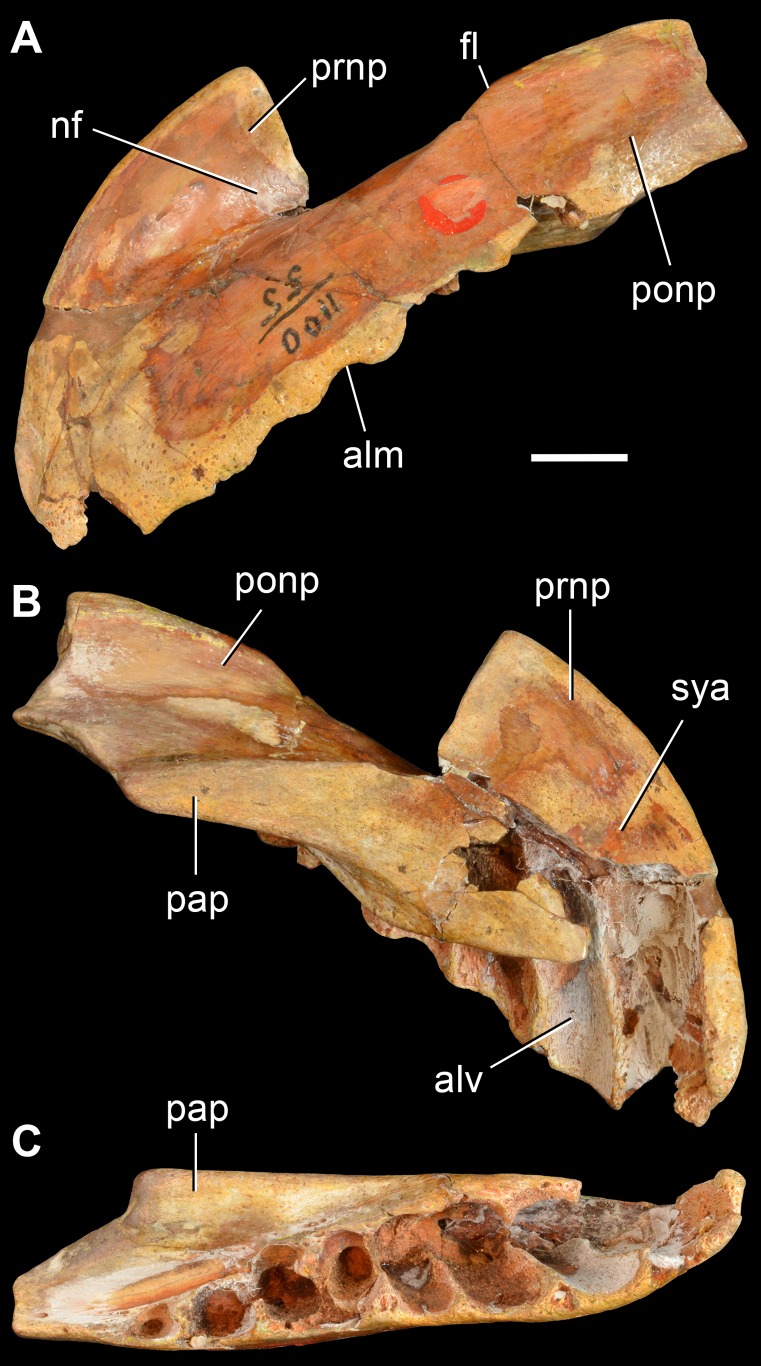
*Archosaurus rossicus*, a proterosuchid archosauriform from the latest Permian of Russia. Left premaxilla (PIN 1100/55, holotype) in lateral (A), medial (B) and ventral (C) views. Abbreviations: alm, alveolar margin; alv, alveolus; fl, lateral flange; nf, narial fossa; pap, palatal process; ponp, postnarial process; prnp, prenarial process; sya, symphyseal area. Scale bar equals 1 cm.

Sennikov [120] subsequently referred to *Archosaurus rossicus* an additional dentary from the same general locality as the holotype and paratypes, as well as two isolated elements from a second locality. These referrals were based on the presence of congruent proterosuchid morphology. Sennikov [120] also revised the taxonomic status of *Archosaurus rossicus* (see also [121], [126]). However, we note that some caution is warranted with regard to the assignment of the paratype and referred specimens from the type locality to *Archosaurus rossicus* given that they come from three different geographical points and different stratigraphic levels within a geographically large locality with a stratigraphic thickness of around 25 meters (Sennikov pers. comm., 2013). In addition to the holotype premaxilla (PIN 1100/55; Fig. 7; Table 3), we consider that the only previously referred specimens of *Archosaurus rossicus* that can be confidently identified as referable to Archosauriformes are the left dentary (PIN 1100/78), skull roof (PIN 1100/48) and possibly a tooth crown (PIN 1100/85). In addition, the cervical vertebrae (PIN 1100/66, 66a, 66b) referred by Tatarinov [88] to *Archosaurus rossicus* possess a morphology that is very similar to and congruent with that of the cervical vertebrae of *Proterosuchus fergusi* (NM QR 1484, GHG 236), and therefore they also possibly belong to an archosauriform.

**Table 3:** Measurements of the holotype of *Archosaurus rossicus* (PIN 1100/55) in millimeters.

**Premaxilla**		
	Length	(83.3)
	Height of the premaxillary body	19.8
	Length of the premaxillary body	73.6
	Maximum height	(34.6)
	Length first alveolus	11.1
	Length second alveolus	7.8
	Length third alveolus	8.3
	Length fourth alveolus	6.7
	Length fifth alveolus	5.6
	Length sixth alveolus	6.3
	Length seventh alveolus	7.1
	Length eighth alveolus	5.4

Values between brackets indicate incomplete measurements and the value given is the maximum measurable. Maximum deviation of the digital caliper equals 0.02 mm but measurements were rounded to the nearest 0.1 millimeter.

The bone identified as a squamosal (PIN 1100/84a) and referred to *Archosaurus rossicus* by Tatarinov [88] does not possess a morphology congruent with that of a squamosal. For example, it lacks a facet for articulation with the quadrate head and possesses a tuberosity on the posterodorsal border of the supposed supratemporal fenestra. Moreover, the anterior process is unusually transversely thick. Accordingly, we doubt the identification of this bone and, as a result, its archosauriform affinities.

The holotype premaxilla of *Archosaurus rossicus* (Fig. 7) differs from most basal archosauromorphs in that the first four premaxillary alveoli open lateroventrally (Fig. 7C), contrasting with the mostly ventrally opening anterior alveoli of *Prolacerta broomi* (BP/1/471), *Sarmatosuchus otschevi* (PIN 2865/68-9), *Tasmaniosaurus triassicus* (UTGD 54655) and *Erythrosuchus africanus* (NHMUK R3592). In addition, the angle formed between the anterior margin of the premaxillary body and the alveolar margin is more acute in *Archosaurus rossicus* than in *Proterosuchus fergusi* (RC 59, SAM-PK-11208) and “*Chasmatosaurus*” *yuani* (IVPP V90002, V4067). Accordingly, the holotype specimen of *Archosaurus rossicus* is diagnostic and, as a result, the genus and species can be considered valid.

*Archosaurus rossicus* has been widely accepted as a proterosuchid archosauriform [88], [120], [121], [127], and quantitative phylogenetic support for this position has been recovered by Nesbitt [59] and by our phylogenetic analysis.

#### Putative proterosuchian from the Late Permian of South Africa

**Figure 8:**
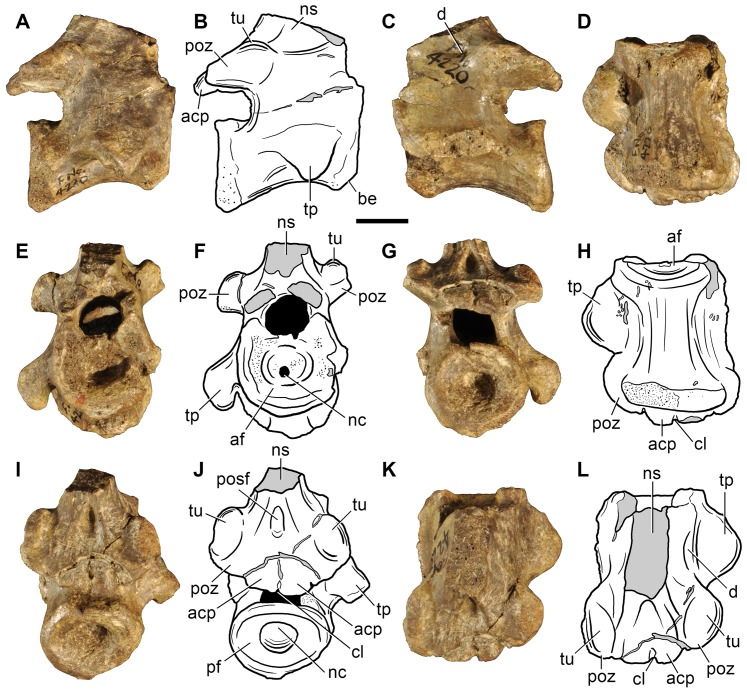
Indeterminate reptiliomorph from the Late Permian–Early Triassic of South Africa. Cervical vertebra (BP/1/4220) in right lateral (A), left lateral (C), ventral (D, H), anterior (E, F), posterior (G), posterodorsal (I, J) and dorsal (K, L) views. Abbreviations: acp, accessory (interpostzygapophyseal) process; af, anterior facet; be, ventral beveling; cl, median cleft; d, depression; nc, notochordal canal; ns, neural spine; tu, tuberosity; pf, posterior facet; poz, postzygapophysis; posf, postspinal fossa; tp, transverse process. Scale bar equals 1 cm.

Cruickshank [90] reported that all known specimens of the early archosauriform *Proterosuchus* came from the lowermost Triassic *Lystrosaurus* Assemblage Zone of South Africa, with one possible exception: a cervical vertebra (BP/1/4220; Fig. 8; Table 4) collected from the Upper Permian upper *Cistecephalus* Assemblage Zone. BP/1/4220 was collected in May 1969 at the farm Gegund 532 near Harrismith in Free State. Cruickshank ([90]: table 1) identified BP/1/4220 as ?*Proterosuchus* sp. and figured the specimen ([90]: fig. 4a). Subsequently, Reisz et al. [14] briefly noted that they could not find any evidence of archosauromorph features in BP/1/4220. Additionally, Reisz et al. ([14]: 443) cast doubts on the exact providence of the specimen and concluded that it could be Triassic rather than Permian in age, but without providing supporting evidence.

**Table 4:** Measurements of the preserved bones of BP/1/4220 in millimeters.

**Cervical vertebra**		
	Centrum length	28.5
	Anterior articular facet height	20.6
	Anterior articular facet width	20.3
	Posterior articular facet height	17.7
	Posterior articular facet width	20.1
	Maximum height of the vertebra	(38.5)
	Length along zygapophyses	(28.8)
	Length of base of neural spine	18.6
	Width of neural spine	9.4
	Width along postzygapophyses	26.4
	Width of accessory processes	13.2
**rib**		
	Length	(109.5)

Values between brackets indicate incomplete measurements and the value given is the maximum measurable. The length along the zygapophyses is the maximum anteroposterior length between the anterior tips of the prezygapophyses and the posterior tips of the postzygapophyses. Maximum deviation of the digital caliper equals 0.02 mm but measurements were rounded to the nearest 0.1 millimeter.

Re-examination of BP/1/4220 revealed a morphology that does not conform to that expected for a basal archosauriform (cf. Reisz et al. [14]). However, we did observe some unusual features not reported in any other tetrapod that we are aware of. Because no detailed description of the specimen has ever been published, we here describe BP/1/4220 in detail for the first time and reassess its possible phylogenetic position.

BP/1/4220 (Fig. 8) is an almost complete vertebra that is not an axis because the transverse processes are well-developed, but may have belonged to the middle cervical series based on the presence of a parallelogram-shaped centrum in lateral view (the anterior articular surface is positioned distinctly dorsal to the posterior surface) and a diapophysis that is placed well below the neural arch (ventral to the level of the dorsal margin of the centrum). The vertebra is notochordal, with an open notochordal canal that is wider than tall and which completely pierces the centrum (Fig. 8: nc). Series of concentric bony laminae surround the notochordal canal, indicating the partial resorption of the notochord during life. In addition, the neurocentral suture is obliterated, suggesting that the animal was not a juvenile at the time of its death [110], and that the presence of an open notochordal canal is therefore not a result of an early ontogenetic stage. The persistence of an open notochordal canal in a non-juvenile individual resembles the condition in multiple lineages of basal reptiliomorphs, parareptiles, basal synapsids, basal sauropsids, basal lepidosauromorphs, and the new archosauromorph species *Aenigmastropheus parringtoni* from the Late Permian of Tanzania ([48], [70], [119], [128]–[134], see below).

The anterior and posterior articular surfaces of the centrum are wider than tall. The anterior and posterior borders of the centrum are strongly beveled on their ventral margin in lateral view (Fig. 8: be), indicating the probable presence of small intercentra. The centrum is slightly transversely compressed at mid-length, and has a spool-shape in ventral view. The ventral surface of the centrum is mostly planar and well differentiated from the lateral surfaces. The lateral surfaces of the centrum are concave in ventral view and possess shallow and poorly defined fossae. The centrum lacks parapophyses and it is likely that the parapophyses would have been placed on the intercentrum, as occurs in several basal amniotes (e.g. *Procolophon trigoniceps*: [135]). Subcentral foramina are absent in BP/1/4220.

In the neural arch, the transverse processes are robust and directed posterolaterally and slightly ventrally. BP/1/4220 completely lacks any development of centrodiapophyseal or paradiapophyseal laminae, contrasting with the condition observed in some caudatans, “pelycosaurian” synapsids, basal diapsids and archosauromorphs (see below). The postzygapophyses are separated from the posterior end of the centrum by a tall and deep notch in lateral view. Only the bases of the prezygapophyses are preserved and they are well separated transversely from one another, as is also the case for the postzygapophyses. The presence of broadly separated zygapophyses contrasts with the condition observed in the cervical vertebrae of most archosauromorphs and some “pelycosaurian” synapsids, in which the zygapophyses are placed close to each other in dorsal view (e.g. *Ophiacodon* sp., MCZ 1426; *Prolacerta broomi*, BP/1/2675; *Protorosaurus speneri*, ZMR MB R2173). The neural arch lacks a prezygodiapophyseal lamina, contrasting with the condition observed in the varanopid *Apsisaurus witteri* ([73]: fig. 6; sensu Reisz et al. [43]) and several archosauromorphs (e.g. *Tanystropheus longobardicus*, SMNS 54628; *Protorosaurus speneri*, BSPG 1995 I 5; *Erythrosuchus africanus*, NHMUK R3592; *Garjainia prima*, PIN 2394/5-16).

The articular facets of the postzygapophyses of BP/1/4220 are oval, anteroposteriorly long and transversely wide, and face ventrally and slightly laterally. The dorsal surfaces of the postzygapophyses possess thick, rounded tuberosities (Fig. 8: tu) that resemble the epipophyses present in the cervical vertebrae of dinosaurs [136], the tanystropheids *Macrocnemus bassanii* (PIMUZ T4822) and *Tanystropheus longobardicus* (SMNS 54630, 54654), and derived rhynchosaurs [137]. However, in contrast to the latter taxa, in BP/1/4220 the tuberosity is situated not on the posterior half of the postzygapophysis, but at the level of the anterior margin of its articular facet. The tuberosity possesses a rugose surface, which may suggest a tendinous attachment.

The neural arch possesses two posteriorly directed median or interpostzygapophyseal processes between the postzygapophyses that lack their most posterior ends (Fig. 8: acp). Despite being damaged posteriorly, the interpostzygapophyseal processes project further posteriorly than do the postzygapophyses. The position of these interpostzygapophyseal processes in BP/1/4420 is similar to the transpostzygapophyseal lamina of trilophosaurids [98] and the accessory intervertebral articular processes of some saurians (i.e. the non-homologous hyposphene of archosauriforms and the zygosphene of squamate lepidosauromorphs and sauropterygians) [138]. The interpostzygapophyseal processes of BP/1/4220 are oval, posteriorly and slightly ventrally oriented, and separated from one another by a deep but transversely narrow median cleft (Fig. 8: cl). The presence of a cleft between the interpostzygapophyseal processes and the posterior extension of the processes beyond the level of the postzygapophyses differs from the morphology of the archosauromorph hyposphene and is in complete contrast with the depressed morphology of the lepidosauromorph zygosphene. No articular facet is discernable on the preserved portions of the interpostzygapophyseal processes of BP/1/4220. The interpostzygapophyseal processes of BP/1/4220 also differ from the accessory articular processes of tangasaurids (e.g. *Hovasaurus* [68]), which are vertically oriented and placed dorsal to the postzygapophyses at the base of the neural spine, and from those of diadectomorphs and seymouriamorphs [139], in which the accessory processes are medioventral projections of the postzygapophyses. The presence of a median cleft and the possible absence of articular facets in the interpostzygapophyseal processes of BP/1/4220 resemble the condition present in the transpostzygapophyseal lamina of trilophosaurids [112], but in the latter taxa the lamina does not extend posteriorly beyond the level of the posterior margin of the postzygapophysis. Accordingly, the condition observed in BP/1/4220 does not match with the morphology of any other tetrapod of which we are aware.

The neural spine is transversely thick at its base and moderately expanded anteroposteriorly (Fig. 8: ns). The neural arch possesses a shallow depression lateral to the base of the neural spine on its left side (Fig. 8: d). This condition resembles that observed in some “pelycosaurian” synapsids (e.g. *Apsisaurus witteri* [73]) and archosauromorphs (e.g. *Protorosaurus speneri*, BSPG 1995 I 5; *Prolacerta broomi*, BP/1/2675), but contrasts with the deeper and better-defined depressions of the araeoscelidians *Araeoscelis gracilis* [128] and *Petrolacosaurus kansensis* [131]. Nevertheless, this depression is absent on the right side of the neural arch of BP/1/4220. The neural spine possesses a very deep and transversely wide postspinal fossa that is well defined laterally by sharp edges forming the posterolateral corners of the neural spine (Fig. 8: posf). The postspinal fossa is not completely preserved dorsally, but it is shallow at its most dorsal preserved portion suggesting that it would have extended only along the ventral portion of the neural spine. The postspinal fossa is subtriangular in posterior view.

Three indeterminate bone fragments and a possible fragment of rib shaft are also preserved in BP/1/4220. The possible rib shaft possesses a plate-like end that becomes rod-like, with an elliptical cross-section, towards the other end of the bone. No articular facet is preserved on this fragment of bone.

We are unable to recognize any synapomorphies that would allow assignment of BP/1/4220 to Archosauromorpha (see also Reisz et al. [14]), Lepidosauromorpha or Sauria. Indeed, BP/1/4220 differs from several archosauromorphs (e.g. the new archosauromorph species *Aenigmastropheus parringtoni*, see below: UMZC T836; *Prolacerta broomi*, BP/1/2675; *Proterosuchus fergusi*, GHG 231) in possessing postzygapophyses that are strongly divergent posteriorly (although this condition is present in *Trilophosaurus* and rhynchosaurs; see below) and the absence of laminae on the neural arch (laminae are also absent in rhynchosaurs; see below). BP/1/4220 further differs from saurians in the absence of parapophyses on the centrum and the extreme transverse thickness of the neural spine at its base. As a result, the assignment of BP/1/4220 to Archosauromorpha by Cruickshank [90] is not followed here.

BP/1/4220 possesses a striking combination of features unknown in any amniote that we are familiar with (e.g. notochordal centrum, thick and anterodorsally oriented neural spine, large tubercle on the dorsal surface of the postzygapophysis, interpostzygapophyseal processes). Although BP/1/4220 appears to represent a distinct amniote taxon we do not erect a new species for it due to the highly incomplete nature of the specimen. BP/1/4220 can be unambiguously assigned to Reptiliomorpha (diadectomorphs+amniotes) based on the presence of a large pleurocentrum (with a reduced intercentrum, if present; cf. Romer [138]). However, we could not identify any feature that would allow the specimen to be assigned to a less inclusive reptiliomorph clade. BP/1/4220 was not included in the phylogenetic analysis conducted here because of its highly incomplete condition, and because of the absence of some major amniote clades in the taxonomic sample of the analysis (e.g. parareptiles). In summary, we interpret BP/1/4220 as belonging to Reptiliomorpha, and it may represent a previously unrecognized reptiliomorph lineage within the Late Permian of South Africa.

#### Specimens identified as either varanopid “pelycosaurs” or basal archosauromorphs from the Permo-Triassic of Uruguay

Piñeiro et al. [140] described multiple isolated dorsal and caudal vertebrae from the Buena Vista Formation of northwestern Uruguay. This sedimentary unit was deposited during the Late Permian and probably also during the Early Triassic as part of the infill of the Paraná Basin [141]. Piñeiro et al. [140] assigned the vertebrae to varanopid “pelycosaurs”, noting strong resemblances to the Permian species *Mycterosaurus longiceps* and *Mesenosaurus romeri*.

Subsequently, Dias-da-Silva et al. [91] stated that the identification of “pelycosaurian” synapsids in the Buena Vista Formation was unwarranted and that the isolated vertebrae described by Piñeiro et al. [140] closely resembled those of the basal archosauromorph *Prolacerta broomi*. Dias-da-Silva et al. [91] concluded that the vertebrae reported from the Buena Vista Formation may belong to a basal archosauromorph or to another kind of diapsid. At the same time, Dias-da-Silva et al. [91] pointed out that the other tetrapods (i.e. temnospondyl and procolophonoid remains) collected from the Buena Vista Formation [142], [143] are not strongly indicative of a Late Permian age.

Our re-examination of the isolated vertebrae described by Piñeiro et al. [140] (FC-DPV 1182, 1183, 1189, 1194, 1199, 1200 and 1333) does not reveal the presence of any archosauromorph synapomorphies in these specimens (e.g. there are no anterior or posterior centrodiapophyseal or prezygodiapophyseal laminae). The overall morphology of these vertebrae is congruent with the vertebrae of basal archosauromorphs (e.g. *Prolacerta broomi*; see also Dias-da-Silva et al. [91]), but also with those of some varanopid “pelycosaurs” [140]. As a result, we do not support an unambiguous assignment of these vertebrae to Archosauromorpha. Nevertheless, some unpublished specimens also collected from the Buena Vista Formation can be assigned to archosauromorphs that are probably closely related to protorosaurs, *Prolacerta* and proterosuchids (MDE pers. obs.). These specimens will be described elsewhere.

In sum, although the Buena Vista Formation yields (currently unpublished) saurian remains, the current poor stratigraphic constraints on its age mean that the putative Permian age of specimens from this unit is ambiguous.

#### “Problematic reptile” from the Late Permian of Tanzania

Parrington [9] described the remains (several vertebrae and some fragmentary forelimb elements) of an enigmatic Permian specimen (UMZC T836) collected in the Ruhuhu Valley of Tanzania. He highlighted the apparent contrast between the primitive appearance of the forelimb bones and the more derived appearance of the vertebrae, with neural arch laminae (“buttresses”) and articular rib facets resembling those of archosaurs. Parrington [9] concluded that the bones of UMZC T836 did not bear close resemblances to any known synapsid, and suggested instead that the specimen might have close affinities with archosaurs because of the vertebral morphology and the presence of hollow limb bones and an ectepicondylar groove on the humerus.

Subsequently, Hughes [144] noted that the vertebrae of UMZC T836 were not as archosaurian in appearance as Parrington originally thought and that laminae on the neural arch also occur in “pelycosaurian” synapsids. Hughes [144] further noted that a notochordal centrum is present in “pelycosaurs”, but is unknown among archosaurs. However, Hughes [144] concluded that the combination of a derived vertebral column and a primitive limb structure occurs in proterosuchian archosauromorphs, and suggested that UMZC T836 might possibly be an “incipient proterosuchian” (i.e. a proterosuchian ancestor). Reig [145] noted that the vertebrae of UMZC T836 were transitional between those of “pelycosaurs” and archosaurs. Charig and Sues [127] listed this specimen as a possible member of Proterosuchidae in their review of “Proterosuchia”, but also highlighted the skepticism raised by Hughes [144] as to the archosaurian affinities of UMZC T836. Gower and Sennikov [121] noted that UMZC T836 is probably indeterminate, but could possibly be archosaurian. Most recently, Ezcurra et al. [5] indicated that UMZC T836 is likely not referable to Archosauriformes (the archosauromorph clade that includes proterosuchians).

**Figure 9:**
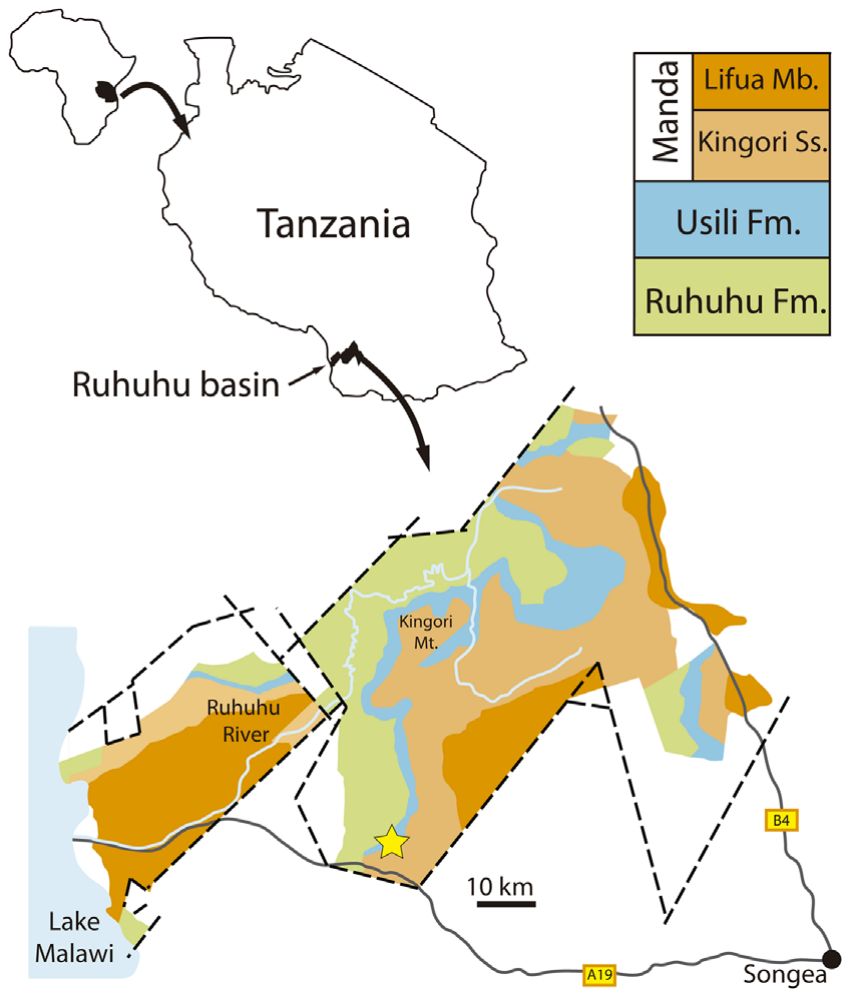
Type locality of *Aenigmastropheus parringtoni* in the Ruhuhu Basin, southwestern Tanzania, Africa. Star indicates the approximate geographic and stratigraphic occurrence of *Aenigmastropheus parringtoni* (locality B35). Abbreviations: Fm, formation; Mb, member; Mt, mountain; Ss, sandstone. Figure modified from Nesbitt et al. [224].

Parrington [9] reported that he collected UMZC T836 in the Ruhuhu Valley of Tanzania in 1933. These fossil-bearing levels correspond to locality B35 of Stockley [146], which is located near the town of Ruanda in the Songea District of southern Tanzania ([146]: plate 38; [147]: fig. 1) (Fig. 9). Stockley [146] considered locality B35 to be part of the “Lower Bone Bed”, corresponding to his K6 horizon of the Songea Series. The K6 horizon is currently assigned to the Usili Formation (formerly the Kawinga Formation) of the Songea Group of the Ruhuhu Basin. Wopfner et al. [148] and Sidor et al. [149] described the Usili Formation as a 260 meters thick fluviolacustrine succession made up of a lowermost conglomeratic interval that is approximately 5 meters thick, grading up into a trough cross-bedded, coarse-grained, sandstone-dominated interval that is 25–40 meters thick, overlain by massive nodular siltstone and laminated mudstone beds with minor ribbon sandstones forming the bulk of the succession.

Sidor et al. [149] recognized a single tetrapod faunal assemblage in the Usili Formation, which includes, in addition to UMZC T836, temnospondyls, pareiasaurs, gorgonopsians, therocephalians, cynodonts, and dicynodonts [147], [149]–[151]. In particular, the locality from which UMZC T836 was collected also yielded an isolated maxilla of a dicynodont listed by Parrington [9] as cf. “*Esoterodon*” *uniseries* (UMZC T969), as well as other dicynodont (UMZC T779, T1170) and gorgonopsid (UMZC T882, T883) remains ([9], [152]; UMZC catalogue and unpublished field notes of Parrington in UMZC collections). Parrington [9] proposed that locality B35 is equivalent in age to the South African horizons that yield *Endothiodon* (currently known in the late *Pristerognathus*, *Tropidostoma*, and early *Cistecephalus* assemblage zones of South Africa: [84], [149], [153]–[155]) because of the presence of cf. “*Esoterodon*” *uniseries* (“*Esoterodon*” is currently considered to be a junior synonym of *Endothiodon* [156]). More recently, Angielczyk *et al.* [155] considered that the common presence of the dicynodonts *Dicynodon huenei* and possibly *Katumbia parringtoni* allow a direct correlation between the faunistic associations of the Usili Formation and the Zambian Upper Madumabisa Mudstone. As a result, the well-supported correlation of the Upper Madumabisa Mudstone with the rocks of the *Cistecephalus* Assemblage Zone in the South African Karoo Basin implies that the Usili Formation can be considered a lateral equivalent of the *Cistecephalus* Assemblage Zone [155], constrained to the middle–late Wuchiapingian (ca. middle Late Permian, 260–255 Ma [157]).

Several authors commented on the phylogenetic relationships of UMZC T836 following the original description of Parrington [9]. However, a detailed redescription, illustrations and comparisons of the specimen are currently lacking. The unusual combination of archosauromorph-like features and amniote plesiomorphies recognized in UMZC T836 by Parrington [9] led us to revisit its anatomy and phylogenetic relationships, and allowed us to recognize this specimen as a new taxon, *Aenigmastropheus parringtoni* gen. et sp. nov.

### Systematic Paleontology

AMNIOTA Haeckel, 1866 [158]

DIAPSIDA Osborn, 1903 [159] sensu Laurin (1991) [73]

SAURIA Gauthier, 1984 [72] sensu Gauthier et al. (1988) [57]

ARCHOSAUROMORPHA Huene, 1946 [160] sensu Dilkes (1998) [4]

?PROTOROSAURIA Huxley, 1871 [161] (new explicit definition)

#### Phylogenetic definition

Protorosauria Huxley, 1871 [161] is a stem-based clade that includes all taxa more closely related to *Protorosaurus speneri* Meyer 1830 [98] than to *Varanus komodoensis* Ouwens 1912 [162] or *Crocodylus niloticus* Laurenti 1768 [163] (new definition).

?PROTOROSAURIDAE Lydekker, 1888 [164] (new explicit definition)

#### Phylogenetic definition

Protorosauridae Lydekker, 1888 [164] is a stem-based clade that includes all taxa more closely related to *Protorosaurus speneri* Meyer 1830 [98] than to *Tanystropheus longobardicus* Bassani 1886 [165], *Prolacerta broomi* Parrington 1935 [166], *Sharovipteryx mirabilis* (Sharov, 1971) [167], *Drepanosaurus unguicaudatus* Pinna 1980 [168] or *Varanus komodoensis* Ouwens 1912 [162] (new definition).

*Aenigmastropheus* gen. nov.

urn:lsid:zoobank.org:act:354E966B-CDA9-4509-84F5-2F130E23B2B5

*Aenigmastropheus parringtoni* sp. nov.

urn:lsid:zoobank.org:act: 78DF791F-C4F4-4592-8C3E-D333C8C91E58

(Figures 10–15, 16B, 17A, D, 18A)

**Figure 10:**
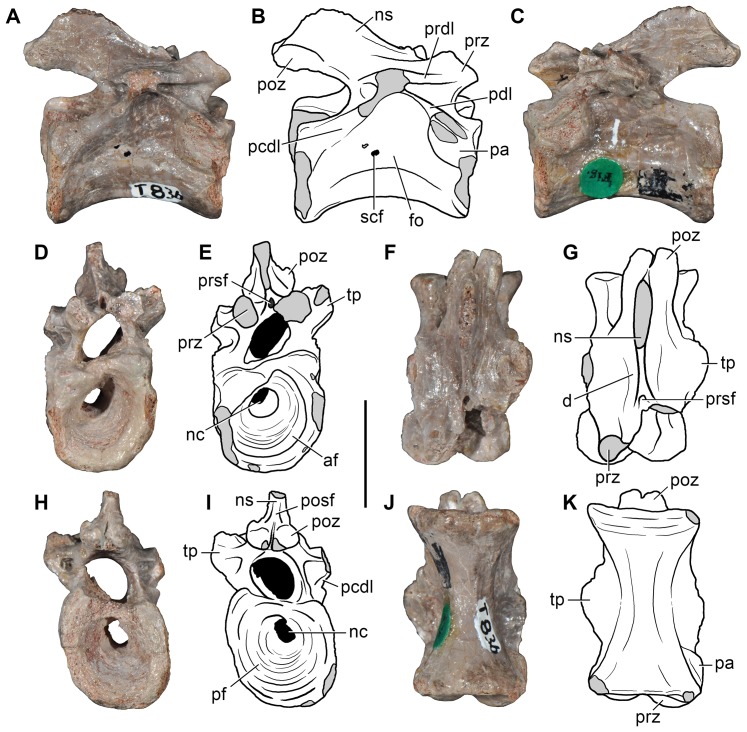
*Aenigmastropheus parringtoni*, an early archosauromorph from the middle Late Permian of Tanzania. Cervical vertebra (vertebra 1 sensu Parrington [9]) (UMZC T836, holotype) in right lateral (A, B), left lateral (C), anterior (D, E), dorsal (F, G), posterior (H, I) and ventral (J, K) views. Abbreviations: af, anterior facet; d, depression; fo, lateral fossa; nc, notochordal canal; ns, neural spine; pa, parapophysis; pcdl, posterior centrodiapophyseal lamina; pdl, paradiapophyseal lamina; pf, posterior facet; posf, postspinal fossa; poz, postzygapophysis; prdl, prezygodiapophyseal lamina; prz, prezygapophysis; prsf, prespinal fossa; scf, subcentral foramen; tp, transverse process. Scale bar equals 1 cm.

**Figure 11:**
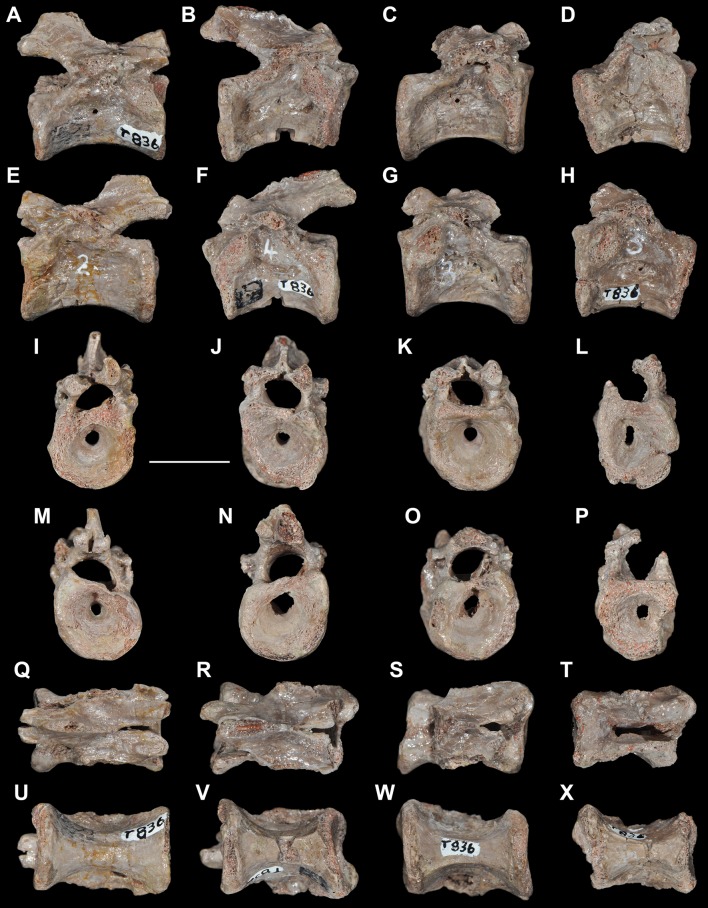
*Aenigmastropheus parringtoni*, an early archosauromorph from the middle Late Permian of Tanzania. Cervico-dorsal vertebrae (UMZC T836, holotype) in right lateral (A–D), left lateral (E–H), anterior (I–L), posterior (M–P), dorsal (Q–T) and ventral (U–X) views. Vertebrae 2 (A, E, I, M, Q, U), 3 (C, G, K, O, S, W), 4 (B, F, J, N, R, V) and 5 (D, H, L, P, T, X) sensu Parrington [8]. Scale bar equals 1 cm.

**Figure 12:**
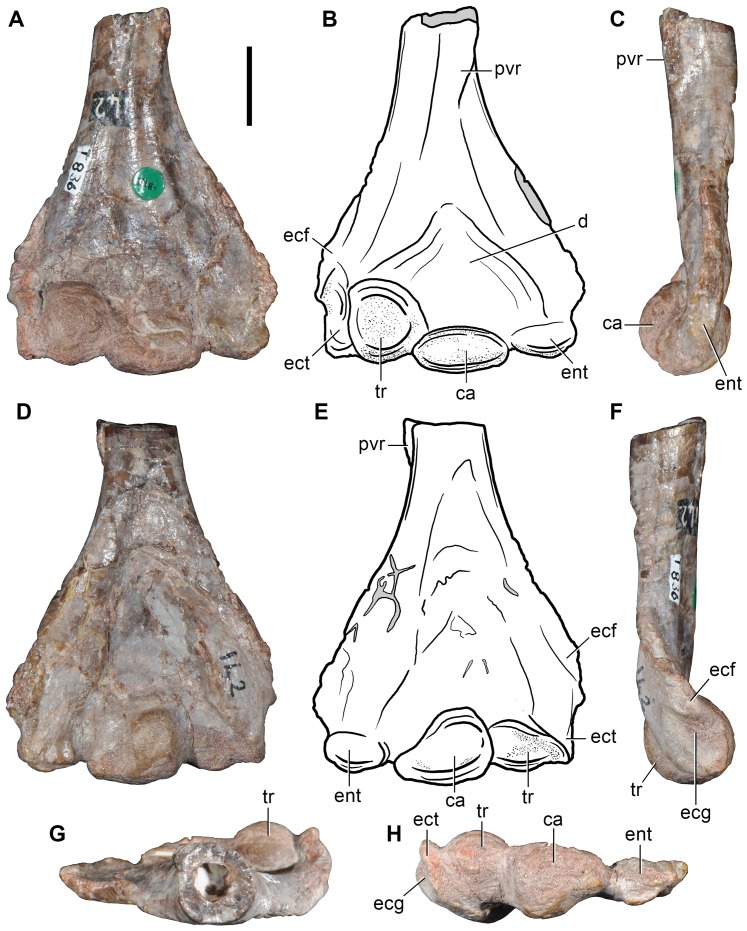
*Aenigmastropheus parringtoni*, an early archosauromorph from the middle Late Permian of Tanzania. Distal half of the right humerus (UMZC T836, holotype) in ventral (A, B), posterior (C), dorsal (D, E), anterior (F), proximal (G) and distal (H) views. Abbreviations: ca, capitellum (radial condyle); d, depression; ecf, ectepicondylar flange; ecg, ectepicondylar groove; ect, ectepicondyle; ent, entepicondyle; pvr, posteroventral ridge; tr, trochlea (ulnar condyle). Scale bar equals 1 cm.

**Figure 13:**
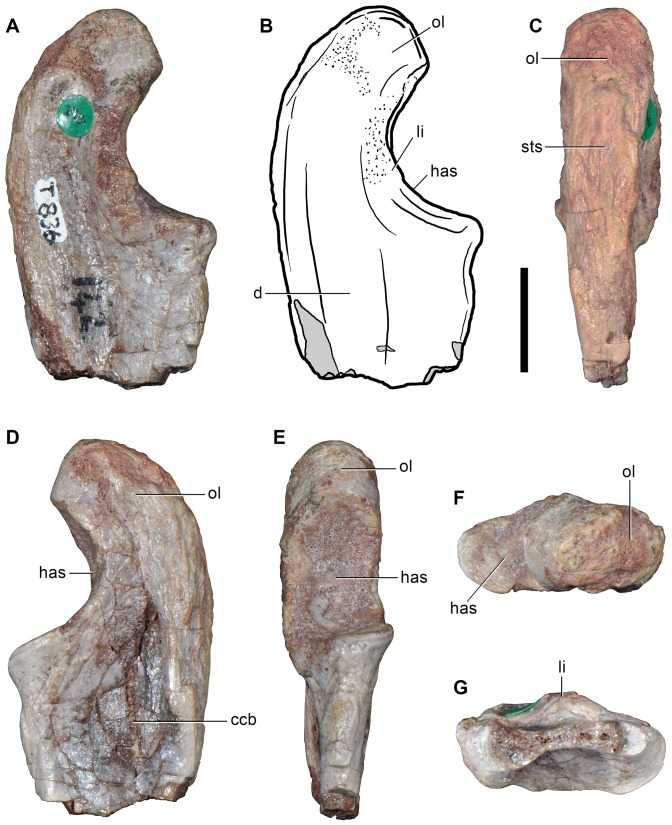
*Aenigmastropheus parringtoni*, an early archosauromorph from the middle Late Permian of Tanzania. Proximal end of the right ulna (UMZC T836, holotype) in anterior (A, B), dorsal (C), posterior (D), ventral (E), proximal (F) and distal (G). Abbreviations: ccb, collapsed cortical bone; d, depression; has, humeral articular surface; li, lip; ol, olecranon; sts, striated surface. Scale bar equals 1 cm.

**Figure 14:**
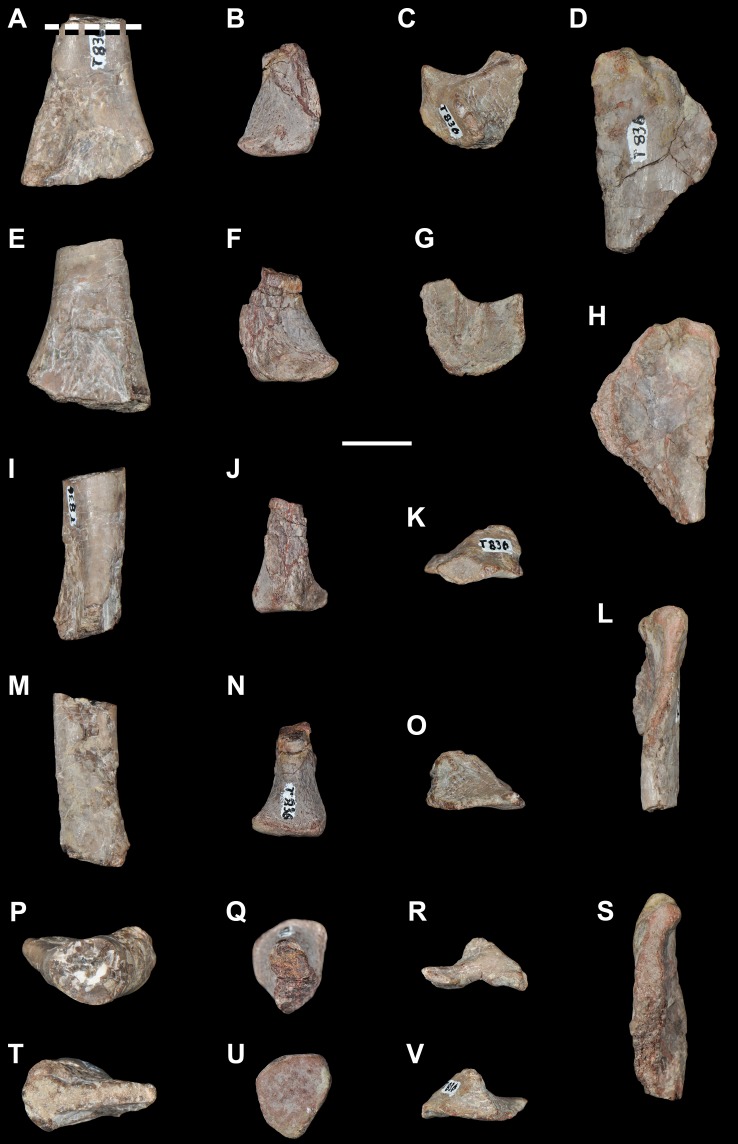
*Aenigmastropheus parringtoni*, an early archosauromorph from the middle Late Permian of Tanzania. Possible left humeral shaft (A, E, I, M, P, T), possible distal end of radius (B, F, J, N, Q, U) and two indeterminate bones (C, D, G, H, K, L, O, R, S, V) (UMZC T836, holotype) in several views. The dashed line indicates the area sampled for the paleohistological study. Scale bar equals 1 cm.

**Figure 15:**
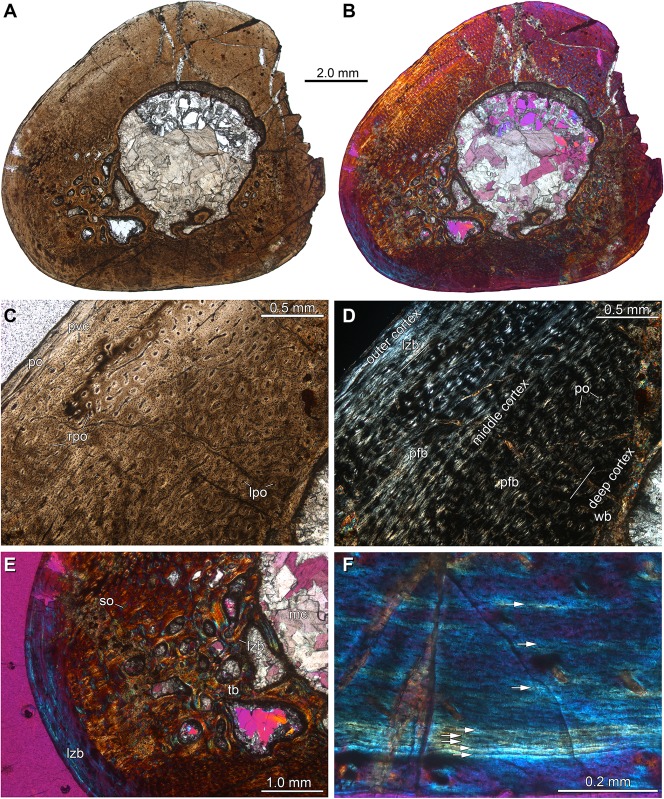
*Aenigmastropheus parringtoni*, an early archosauromorph from the middle Late Permian of Tanzania. Paleohistological slices of possible left humerus (UMZC T836, holotype). Overview of the bone section in normal light (A) and cross-polarized light with lambda compensator (B); upper left quadrant showing the complete section of the cortex, in which the medullary cavity is to the lower right of the image, in normal (C) and cross-polarized light (D); lower left quadrant showing the complete section of the cortex in cross-polarized light with lambda compensator (E); and close-up of the outer part of the periosteal cortex in cross-polarized light with lambda compensator (F). White arrows in (F) indicate lines of arrested growth (LAGs). Abbreviations: lpo, longitudinal primary osteons; lzb, lamellar-zonal bone; mc, medullary cavity; pfb, parallel-fibred bone; po, primary osteons; pvc, primary vascular canal; rpo, reticular primary osteons; so, secondary osteons; tb, trabecular bone; wb, woven bone.

**Figure 16:**
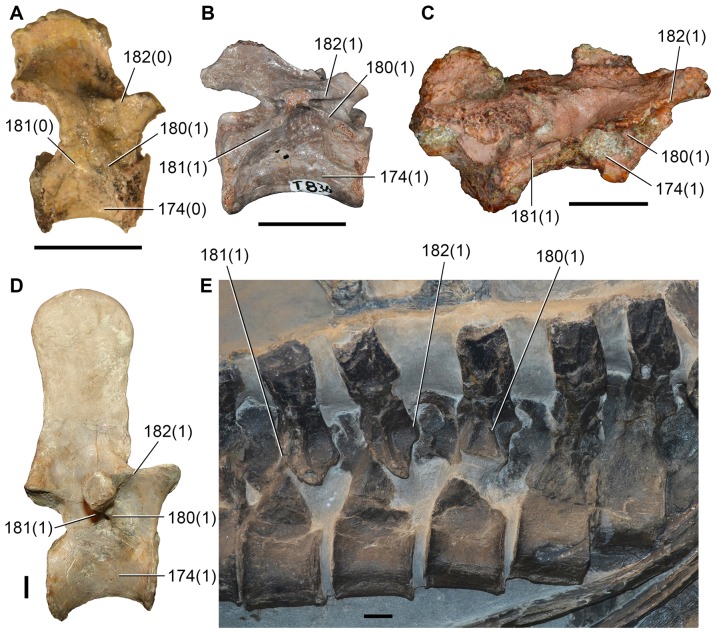
Character states supporting the phylogenetic positions of *Aenigmastropheus parringtoni* and *Eorasaurus olsoni*. Anterior and middle dorsal vertebrae of *Youngina capensis* (BP/1/3859) (A), *Tanystropheus longobardicus* (SMNS 55341, reversed) (D) and *Helveticosaurus* (PIMUZ T4352, holotype, reversed) (E) and middle-posterior cervical vertebrae of *Aenigmastropheus parringtoni* (UMZC T836, vertebra 1, holotype, reversed) (B) and *Eorasaurus olsoni* (PIN 156/109) (C) in right lateral views. Scale bar equals 5 mm in (A) and 1 cm in (B–E).

**Figure 17:**
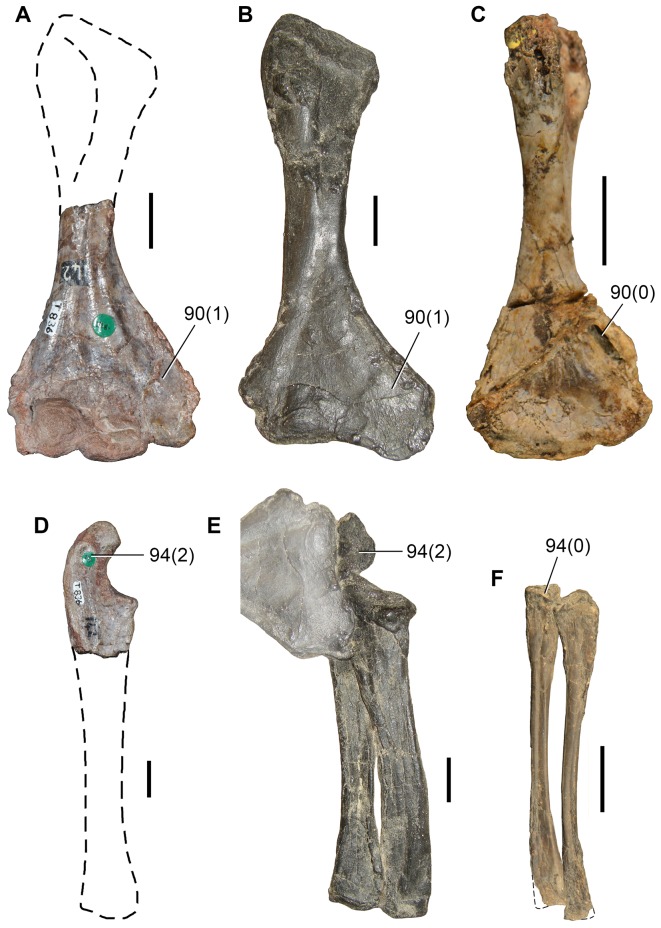
Character states supporting the phylogenetic position of *Aenigmastropheus parringtoni*. Humeri of *Aenigmastropheus parringtoni* (UMZC T836, holotype) (A), *Protorosaurus speneri* (BSPG 1995 I 5, cast of WMSN P47361, reversed) (B) and *Youngina capensis* (BP/1/3859) (C) in ventral views; ulna of *Aenigmastropheus parringtoni* (UMZC T836, holotype) (D) and ulnae and radii of *Protorosaurus speneri* (BSPG 1995 I 5, cast of WMSN P47361) (E) and *Macrocnemus bassanii* (PIMUZ T4355) (F) in anterior views. Scale bar equal 1 cm in (A, B, D–F) and 5 mm in (C).

**Figure 18:**
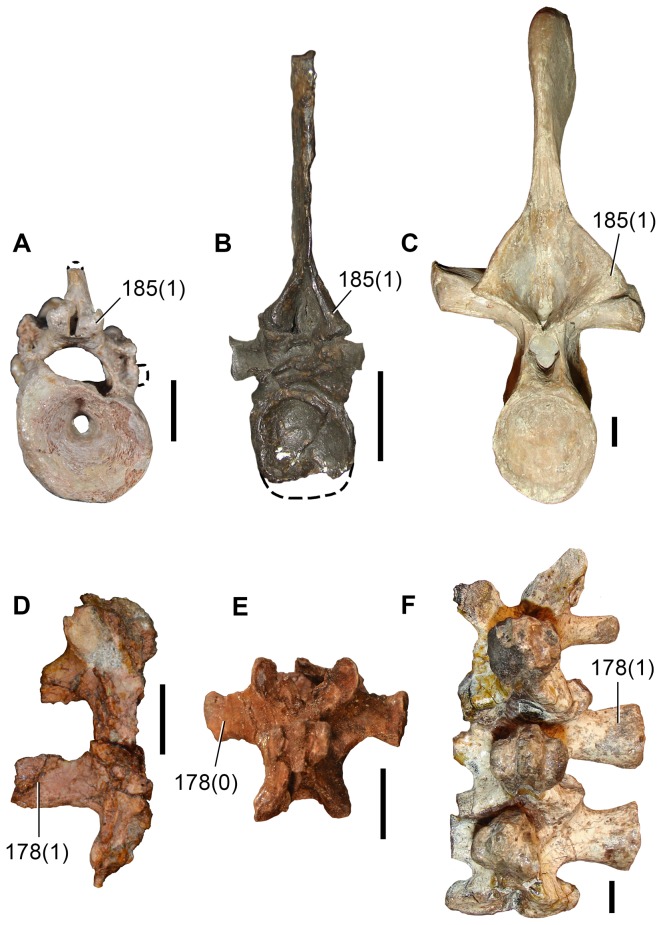
Character states supporting the phylogenetic positions of *Aenigmastropheus parringtoni* and *Eorasaurus olsoni*. Cervical and dorsal vertebrae of *Aenigmastropheus parringtoni* (UMZC T836, vertebra 2, holotype) (A), *Protorosaurus speneri* (ZMR MB R2173) (B) and *Tanystropheus longobardicus* (SMNS 55341) (C) in posterior views, and cervico-dorsal vertebrae of *Eorasaurus olsoni* (PIN 156/110) (D), *Prolacerta broomi* (BP/1/2675) (E) and *Proterosuchus fergusi* (GHG 363) (F) in dorsal views. Scale bars equal 5 mm in (A, E) and 1 cm in (B, C, D, F).

#### Etymology

The generic name (“enigmatic vertebra”) is derived from the Latin word *aenigma* (enigmatic) and the Greek word *stropheus* (vertebra) in allusion to the problematic taxonomic history of the holotype specimen. The specific name honors the British paleontologist Dr. F. R. Parrington for his contribution to the understanding of Permo-Triassic amniotes and his discovery and initial description of the holotype specimen.

#### Holotype

UMZC T836, partial postcranial skeleton including five posterior cervical–anterior dorsal vertebrae, distal half of the right humerus, fragment of probable left humeral shaft, proximal end of the right ulna, and three indeterminate fragments of bone (one of which may represent part of a radius) (Figs. 10–15).

#### Diagnosis

*Aenigmastropheus parringtoni* is a medium-sized archosauromorph saurian distinguished from other amniotes by the following combination of features: posterior cervical and anterior dorsal vertebrae notochordal, with well-developed anterior and posterior centrodiapophyseal and prezygodiapophyseal laminae, and sub-triangular neural spines in lateral view; humerus with a strong diagonal ridge on the anterior surface of the shaft (autapomorphy); humerus with strongly developed capitellum (radial condyle) and trochlea (ulnar condyle) and without entepicondylar and ectepicondylar foramina; ulna with strongly developed olecranon process forming a single ossification with the rest of the bone.

#### Locality

Locality B35 of Stockley, close to the road near Ruanda, Songea District, Ruhuhu Valley, southern Tanzania [9], [146] (Fig. 9).

#### Horizon and Age

Usili Formation (formerly the Kawinga Formation), deposited during the middle–late Wuchiapingian (middle Late Permian; [155]), Songea Group, Ruhuhu Basin.

#### Comments

*Aenigmastropheus* possesses a striking combination of features that complicates assessment of its phylogenetic relationships (see Discussion). However, these features also support its distinctiveness from other amniotes, including diapsids and “pelycosaur” synapsids. The combination of posterior centrodiapophyseal and prezygodiapophyseal laminae, zygapophyses that are positioned close to each other medially, and the absence of an entepicondylar foramen in the distal end of the humerus distinguish *Aenigmastropheus* from non-archosauromorph sauropsids. *Aenigmastropheus* differs from the enigmatic neodiapsid *Helveticosaurus zollingeri* due to the presence of low neural arches with subtriangular neural spines in the cervico-dorsal transition region, well-developed distal condyles of the humerus and a well-developed olecranon process on the proximal end of the ulna. Within Archosauromorpha, *Aenigmastropheus* differs from other basal members of the group due to the presence of notochordal vertebrae, a strongly developed olecranon process as part of a single ossification with the rest of the ulna (convergently acquired in some crown archosaurs) and a thick posteroventral ridge along the humeral shaft. The latter character is an autapopmorphy of *Aenigmastropheus parringtoni* among basal diapsids.

#### Anatomical description

Parrington [9] apparently considered all the bones catalogued as UMZC T836 to belong to a single individual. The five preserved vertebrae do indeed possess a congruent morphology and similar size, consistent with belonging to a single individual (Figs. 10, 11; Table 5). The right humerus and ulna are also consistent in size with belonging to a single individual, and the trochanter (ulnar condyle) of the humerus fits quite well when articulated with the proximal articular facet of the ulna (Figs. 12, 13; Table 6). However, assessing the assignment of the axial and appendicular elements to a single individual is less straightforward. The ratio between the posterior widths of the centra and the maximum distal width of the humerus ranges between 0.29–0.33 in *Aenigmastropheus parringtoni*. This range is very similar to or overlaps the ratio observed in basal reptiliomorphs (e.g. 0.31 in the diadectomorph *Diadectes* [169]), synapsids (e.g. 0.35–0.37 in the varanopid *Varanops*, TMM 43628-1 in Reisz et al. [43]; 0.27–0.29 in *Ophiacodon mirus*, FMNH (WM) 671 in Romer and Price [95]; 0.27–0.32 in *Dimetrodon loomsi*, FMNH (WM) 114 in Romer and Price [95]) and diapsids (e.g. 0.38–0.41 in the archosauriform *Erythrosuchus africanus*, SAM-PK-905). Accordingly, this ratio supports the interpretation of Parrington [9] that all the bones of UMZC T836 belong to a single individual.

**Table 5:** Measurements of the preserved axial bones of *Aenigmastropheus parringtoni* nov. gen. et nov. sp. (UMZC T836) in millimeters.

Vertebrae (sensu Parrington)	1	2	3	4	5
Centrum length	16.4	16.5	15.17	16.6	(15.7)
Anterior articular facet height	10.2	11.3	11.2	(11.3)	11.5
Anterior articular facet width	(9.9)	10.8	11.4	(11.0)	10.4
Posterior articular facet height	11.6	(10.9)	10.9	10.8	(10.4)
Posterior articular facet width	10.2	11.0	11.3	11.3	(9.8)
Maximum height of the vertebra	(19.8)	(18.6)	(17.4)	(19.5)	(16.9)
Length along zygapophyses	(19.5)	(19.1)	(12.7)	(17.7)	(10.3)

Values between brackets indicate incomplete measurements (due to post-mortem damage) and the value given is the maximum measurable. The length along the zygapophyses is the maximum anteroposterior length between the anterior tips of the prezygapophyses and the posterior tips of the postzygapophyses. Maximum deviation of the digital caliper is 0.02 mm but measurements were rounded to the nearest 0.1 millimeter.

**Table 6:** Measurements of the preserved forelimb bones of *Aenigmastropheus parringtoni* nov. gen. et nov. sp. (UMZC T836) in millimeters.

**Humerus**		
	Length	(46.4)
	Width close to mid-shaft	9.5
	Depth close to mid-shaft	10.0
	Perimeter close to mid-shaft	32
	Distal width	34.1
	Distal depth	10.5
	Width of entepicondyle	7.6
	Depth of entepicondyle	5.9
	Width of trochlea	12.0
	Depth of trochlea	9.2
	Width of capitellum	8.9
	Depth of capitellum	8.7
	Width of ectepicondyle	3.3
	Depth of ectepicondyle	8.2
**Ulna**		
	Length	(37.5)
	Length of articular facet for humerus	15.5
	Width of articular facet for humerus	10.0
	Depth at distal broken surface	15.9
	Width at distal broken surface	4.8

Values between brackets indicate incomplete measurements (due to post-mortem damage) and the value given is the maximum measurable. For the humerus the width is measured in the anteroposterior plane and the depth in the dorsoventral plane. Maximum deviation of the digital caliper is 0.02 mm but measurements were rounded to the nearest 0.1 millimeter. The perimeter close to mid-shaft was rounded to the nearest millimeter because the measurement cannot be made directly with the caliper.

The bones are generally well preserved, but possess some damaged surfaces, some of which possibly result from preparation with acetic acid. In a few areas the cortical bone has collapsed or has broken away, and some degree of post-mortem distortion is evident in some elements (most notably in the vertebrae).

Five presacral **vertebrae** are preserved (Figs. 10, 11), and exhibit slight post-mortem distortion, with the left sides (e.g. zygapophyses and transverse processes) of some of the vertebrae having being displaced dorsally relative to the right side. Unfortunately, the prezygapophyses, transverse processes and neural spines are incomplete in all of the vertebrae. Parrington ([9]: fig. 1) designated the vertebrae of UMZC T836 as specimens “1”–“5” for descriptive purposes (hereafter referred as vertebrae 1–5, retaining Parrington's original numbering), and these numbers are written on the left lateral surfaces of the elements. Although we cannot assess with confidence whether or not the preserved vertebrae represent a continuous series, vertebra 4 at least articulates well at its posterior end with vertebra 3 and vertebra 3 at its posterior end with vertebra 5. Thus, it is likely that these three were continuous elements. Our interpretation of their order therefore differs from the numbering used by Parrington. In addition, all five of the vertebrae were likely close to one another within the axial series based on the similar positions of the parapophyses and the similar morphologies of the laminae of the neural arches. The parapophyses are placed primarily on the anterodorsal corners of the centra and extend only a short distance onto the base of the neural arch. Thus, as Parrington [9] noted, these vertebrae likely correspond to the region of the cervico–dorsal transition. Vertebra 1 is here interpreted as the most anterior preserved element because of the relatively ventral position of the parapophysis. Vertebrae 2 and 4 most likely successively followed vertebra 1, because they possess slightly more dorsally placed parapophyses and similarly elongated centra. The most posterior preserved elements seem to be vertebrae 3 and 5, with vertebra 5 the most posterior of the two. These elements exhibit more dorsally positioned parapophyses and likely correspond to anterior dorsal vertebrae.

All the preserved vertebrae possess a completely open notochordal canal (Fig. 10: nc), but the notochordal canal is still connected to the neural canal through an hourglass-shaped opening in at least some elements [9]. This is best observed in vertebra 5, in which most of the neural arch is broken away. The neurocentral sutures are closed, without any trace of the suture remaining visible on the external surface. Thus, UMZC T836 was likely not a juvenile individual on the basis of the closed neurocentral sutures, but possibly also not fully-grown based upon the connected notochordal and neural canals [9].

The centra are anteroposteriorly elongated, with a length to anterior height ratio of 1.61 in vertebra 1, 1.46 in vertebra 2, and 1.40 in vertebra 3 (Table 5), resembling the elongated cervical vertebrae of some basal synapsids (e.g. *Apsisaurus witteri* [73]) and several diapsids (e.g. *Protorosaurus speneri*, BSPG 1995 I 5; *Araeoscelis gracilis* [128]; *Endennasaurus acutirostris* [170]; *Boreopricea funerea* [105]; *Trilophosaurus buettneri* [44]). Vertebrae 1 and 2 have sub-rectangular centra in lateral view. By contrast, in vertebrae 3 and 4 the posterior articular surface terminates in a more ventral position than the anterior one in lateral view. This condition would likely have resulted in a dorsally curved cervico–dorsal transition in lateral view, when the vertebrae were articulated with one another. The condition in vertebra 5 cannot be determined due to extensive damage to the posterior end of the centrum. In ventral view, the centra are hourglass-shaped, being transversely constricted at mid-length, as commonly occurs in many amniote lineages [171]. In lateral view, the ventral margin of the centrum is arched upwards. None of the preserved vertebrae possess a transversely thin median ventral keel, contrasting with the condition commonly found among the cervico–dorsal vertebrae of some basal parareptiles (e.g. *Millerosaurus pricei* [129]; *Procolophon trigoniceps* [135]), some “pelycosaurian” synapsids (e.g. *Apsisaurus witteri* [73]; *Varanops brevirostris* [172]), araeoscelidians (e.g. *Petrolacosaurus kansensis* [131]; *Araeoscelis gracilis* [128]) and some basal archosauromorphs (e.g. *Trilophosaurus buettneri* [44]; *Euparkeria capensis*, SAM-PK-5867). Instead, the ventral surface of the centrum is flattened in vertebrae 3 to 5, with subtle longitudinal ridges laterally delimiting these planar ventral surfaces. In vertebrae 1 and 2 this flattening of the ventral surface is less well developed.

The ventral half of the lateral surface of the centrum is planar to very gently concave in all the vertebrae; more dorsally, the centrum possesses a deeper longitudinal fossa without well-defined margins, directly below the inferred position of the neurocentral suture (Fig. 10: fo). The presence of lateral fossae in the vertebral centrum has been also described for the dorsal vertebrae of probable choristoderan basal diapsids (e.g. *Pachystropheus rhaeticus* [173]) and the presacral vertebrae of numerous archosauriforms (e.g. *Koilamasuchus gonzalezdiazi* [174]; *Tarjadia ruthae* [175]; *Erythrosuchus africanus*, NHMUK R3592; *Euparkeria capensis*, UMZC T692j; *Cuyosuchus huenei*, MCNAM 2669; *Pseudopalatus mccauleyi* [110]; *Arizonasaurus babbitti* [176]; *Aetosauroides scagliai* [177]; *Marasuchus lilloensis*, PVL 3870; *Pantydraco caducus* [178]).

Parrington [9] described foramina that pierce the lateral faces of the centra (Fig. 10: scf). These foramina lie within the lateral fossae in UMZC T836. A pair of foramina seems to be present on the right side of vertebra 1, but only the ventral margin of the most ventral foramen can be confidently identified as a natural border. Furthermore, it should be noted that Parrington ([9]: fig. 1A_1_) only figured one foramen for this vertebra, which corresponds to the more ventral of the two openings currently present. Accordingly, the second “foramen” currently observed on vertebra 1 seems to be a break in the lateral surface of the centrum. The foramen of vertebra 1 is positioned slightly posterior to the mid-length of the centrum. No foramen is observed within the lateral fossa on the left side of the same vertebra. In vertebra 2 a well-defined circular foramen with well-preserved natural borders is present on the right side of the element, being of similar size and placed in the same position to that of vertebra 1. A circular foramen with well-defined natural borders is present on both right and left sides of vertebra 3, and is identical in position to the foramina of the more anterior elements, but the foramen on the left side is considerably smaller than that on the right side. In vertebra 4, a foramen is also present in the right side in an identical position to the foramina of the other vertebrae, but the pair of foramina figured by Parrington [9] on the left side of this vertebra cannot be identified and the surface of the left side seems instead to be devoid of foramina. Finally, a foramen is observed on the left side of vertebra 5, but the condition on the right side cannot be assessed due to damage. Accordingly, the foramina are irregular in their occurrence on each vertebra [9], but usually at least one foramen is present on at least one side of the centrum. The positions, sizes and shapes of the foramina are similar through the preserved vertebrae.

Parrington [9] considered that these foramina would have had a nutritive function and were related to the persistence of the notochord. A foramen of similar shape and position to that of *Aenigmastropheus parringtoni* is present and termed a subcentral foramen in several tetrapods, such as amphiumid caudatans (e.g. *Amphiuma means* [179]), “younginiforms” (e.g. *Acerosodontosaurus piveteaui* [70]), sauropterygians (e.g. *Bobosaurus forojuliensis* [180]), and basal lepidosauromorphs (e.g. *Gephyrosaurus bridensis* [48]). Nevertheless, subcentral foramina occur in amniotes with both notochordal and non-notochordal vertebrae (e.g. sauropterygians [180]). Similar circular vascular foramina are also present in the non-notochordal vertebrae of extant lepidosaurs and crocodiles [114], [180]–[182], which sometimes occur within a lateral fossa that is associated with fat deposits (e.g. *Crocodylus acutus* [182]). Accordingly, the nutritive function for the subcentral foramina in *Aenigmastropheus parringtoni* proposed by Parrington [9] seems reasonable but their proposed association with the notochordal canal is ambiguous.

The anterior and posterior articular surfaces of the vertebrae are roughly sub-circular in outline, being slightly higher than wide in vertebra 2 and 5, and slightly wider than tall in vertebra 3. In all the vertebrae both anterior and posterior articular surfaces are strongly amphicoelous and the centrum is completely pierced by a notochordal canal positioned slightly dorsal to the center of the centrum in anterior or posterior view (Fig. 10: nc). The presence of a notochordal canal in *Aenigmastropheus parringtoni* resembles the condition observed in several basal reptiliomorphs (e.g. *Tseajaia campi* [130]), parareptiles (e.g. *Millerosaurus pricei* [129]; *Procolophon trigoniceps* [135]), basal synapsids (e.g. *Casea broilii*, *Archaeothyris florensis*, *Ophiacodon* sp., *Mycterosaurus longiceps*, *Mesenosaurus romeri*, *Aerosaurus wellesi*, *Varanops brevirostris*, *Varanodon agilis*, *Archaeovenator hamiltonensis*, *Apsisaurus witteri* [73], [43]: Appendix S2, [95], [172]), basal sauropsids (e.g. *Captorhinus aguti* [183]; *Coelurosauravus jaekeli* [132]; *Petrolacosaurus kensensis* [131]; *Araeoscelis gracilis* [128]; *Acerosodontosaurus piveteaui* [70]; *Youngina capensis*, BP/1/3859), the enigmatic neodiapsids *Helveticosaurus zollingeri* [133] and *Hypuronector limnaios* [134], and basal lepidosauromorphs (e.g. *Gephyrosaurus bridensis* [48]; *Planocephalosaurus robinsonae* [119]). The presence of a notochordal canal was described in the basal archosauromorph *Jesairosaurus lehmani* [74], but unequivocal evidence for the presence of this feature could not be identified during direct restudy of the specimens of this taxon (in the holotype, ZAR 06, the center of an anterior dorsal centrum that is exposed in cross-section is filled by quartz crystals, whereas in ZAR 13 the exposed section of the dorsal vertebra possesses several trabeculae, but no notochordal canal).

The dorsal borders of the anterior articular surfaces of vertebrae 1, 3 and 4 possess a subtle notch at the midline, whereas in vertebrae 2 and 5 this border is not preserved due to damage. The centra do not possess beveled surfaces or facets for articulation with intercentra at the ventral margins of the articular surfaces, but it cannot be assessed with certainty whether intracentra were present or not. The parapophyses are not raised on peduncles, and they are oval, with the long axis being orientated posterodorsally in lateral view. The articular surfaces of the parapophyses are slightly concave and bounded by thick lips.

The most complete neural arches are preserved in vertebrae 1, 2 and 4. Vertebra 3 preserves the pedicles of the neural arch and the base of the prezygapophyses, whereas in vertebra 5 only parts of the neural arch pedicles are available. In all of the available vertebrae of *Aenigmastropheus parringtoni*, the dorsoventral height of the neural arch, from its base up to the base of the neural spine, is lower than that of the centrum. A similar condition is usually found in the posterior cervical and anterior dorsal vertebrae of basal amniotes, such as the synapsids *Apsisaurus witteri* [73] and *Varanops brevirostris* [172], and the diapsids *Araeoscelis gracilis* [128], *Petrolacosaurus kensensis* [131], *Youngina capensis* (BP/1/3859), *Prolacerta broomi* (BP/1/2675), *Tanystropheus longobardicus* (PIMUZ T2817), *Macrocnemus bassanii* (PIMUZ T2472) and *Protorosaurus speneri* (BSPG 1995 I 5). By contrast, in the basal lepidosaurs *Gephyrosaurus bridensis* [48] and *Planocephalosaurus robinsonae* [119] the neural arch is considerably higher than the centrum.

The neural canal of *Aenigmastropheus parringtoni* is oval in outline in the less-deformed vertebrae, being wider than tall. The base of the transverse process is anteroposteriorly short and dorsoventrally compressed, and did not extend onto the lateral margin of the prezygapophysis. A series of thin and well-developed laminae connect the transverse processes with other structures in all the preserved vertebrae [9]. A paradiapophyseal lamina connects the transverse process with the parapophysis (Fig. 10: pdl), whereas a posterior centrodiapophyseal lamina extends posteroventrally from the transverse process towards the posterodorsal corner of the centrum (Fig. 10: pcdl). The posterior centrodiapophyseal lamina becomes thicker and lower posteriorly. The paradiapophyseal and posterior centrodiapophyseal laminae bound a shallow concave depression that is separated from the lateral fossa of the centrum by a gently convex surface, and which can be recognized as a centrodiapophyseal fossa [41]. A prezygodiapophyseal lamina connects the transverse process with the prezygapophysis (Fig. 10: prdl), and bounds together with the paradiapophyseal lamina a deep and sub-triangular prezygapophyseal centrodiapophyseal fossa that opens anterolaterally. The transverse process extends posteriorly as a thin ridge. However, this ridge cannot be identified as a postzygodiapophyseal lamina in a strict sense, because it does not reach the postzygapophysis (see Wilson [40]), but it is topologically equivalent with such a feature. The ridge extending from the posterior end of the transverse process and the posterior centrodiapophyseal lamina bound a deep and sub-triangular depression, which is located in the same position as the postzygapophyseal centrodiapophyseal fossa of archosaurs (see Wilson et al. [41]). This depression is considerably shorter anteroposteriorly than the prezygapophyseal centrodiapophyseal fossa.

The presence of well-developed laminae in the neural arch is also observed in some caudatans, basal synapsids, basal diapsids and several archosauropomorphs. For example, the ophiacodontid synapsid *Ophiacodon* sp. (MCZ 1426), the basal diapsids *Acerosodontosaurus piveteaui* (MNHN 1908-32-57) and *Youngina capensis* (BP/1/3859), and the basal archosauriforms *Proterosuchus fergusi* (SAM-PK-K140, GHG 363) and “*Chasmatosaurus*” *yuani* (IVPP V2719) have paradiapophyseal or anterior centrodiapophyseal laminae. The amphiumid caudatan *Amphiuma means* [179] has anterior and posterior centrodiapophyseal laminae (in *Amphiuma* the structures have been termed alar processes [sensu Gardner [179]] but they are considered here as topologically equivalent to amniote laminae, although not homologous). The varanopid synapsid *Apsisaurus witteri* ([73]: fig. 6; sensu Reisz et al. [43]) and the basal archosauromorphs *Prolacerta broomi* (BP/1/2675), *Tasmaniosaurus triassicus* (UTGD 54655) and *Sarmatosuchus otschevi* (PIN 2865/68) possess both prezygodiapophyseal and anterior centrodiapophyseal (or paradiapophyseal) laminae. The basal archosauromorph *Trilophosaurus buettneri* possesses prezygodiapophyseal and postzygodiapophyseal laminae (S. Nesbitt pers. comm. 2013; USNM mounted skeleton, MDE pers. obs.). Finally, the paradiapophyseal or anterior centrodiapophyseal, posterior centrodiapophyseal and prezygodiapophyseal laminae are all present, as in *Aenigmastropheus parringtoni*, in the enigmatic neodiapsid *Helveticosaurus zollingeri* (PIMUZ T4352), the basal archosauromorphs *Tanystropheus longobardicus* (SMNS 54628, [111]: fig. 52–54), *Protorosaurus speneri* (BSPG 1995 I 5), *Spinosuchus caseanus* [112] and *Macrocnemus bassanii* (PIMUZ T2472, 4822; although the presence of a posterior centrodiapophyseal lamina cannot be determined in this species), the basal archosauriforms *Erythrosuchus africanus* (NHMUK R3592 [99]), *Garjainia prima* (PIN 2394/5-16), *Shansisuchus shansisuchus* ([184]: fig. 21) and *Euparkeria capensis* (UMZC T921), and several archosaurs (e.g. *Bromsgroveia walkeri* [114]; *Hypselorhachis mirabilis* [115]; *Silesaurus opolensis* [116]; *Herrerasaurus ischigualastensis*, PVSJ 373).

Only the bases of the prezygapophyses are preserved in vertebrae 1–4, and the prezygapophyses are missing completely in vertebra 5. The prezygapophyses are upturned in lateral view, being anterodorsally directed in all the vertebrae (Fig. 10: prz), which is in agreement with the wide notch formed by the postzygapophyses and the posterodorsal corner of the centra in lateral view. The prezygapophyses possess a thin, medially developed ridge that runs along the ventromedial edge of the articular surface in vertebrae 1 and 2, and along the mid-height of the structure in vertebrae 3 and 4. As a result, the prezygapophyses of vertebrae 1 and 2 are L-shaped in cross-section and those of vertebrae 3 and 4 are T-shaped. These ridges constrain transversely the space between the prezygapophyses at the midline to an extremely narrow longitudinal notch. The most complete prezygapophysis, on the left side of vertebra 2, indicates that, at least in this vertebra, the prezygapophyses extended anteriorly beyond the anterior margin of the centrum.

The postzygapophyses are short in all the preserved vertebrae and poorly laterally distinguished from the base of the neural spine (Fig. 10: poz), resembling the condition observed in most archosauromorphs (e.g. *Protorosaurus speneri*, ZMR MB R2173; *Prolacerta broomi*, BP/1/2675; *Proterosuchus fergusi*, GHG 231) and some “pelycosaurian” synapsids (e.g. *Varanops brevirostris* [172]; *Ophiacodon* sp., MCZ 1426). By contrast, the zygapophyses are laterally deflected from the rest of the neural arch in most basal sauropsids and lepidosaurs, such as *Araeoscelis gracilis* [128], *Petrolacosaurus kansensis* [131], *Youngina capensis* (BP/1/3859), *Gephyrosaurus bridensis* [48] and *Planocephalosaurus robinsonae* [119]. In vertebrae 1 and 2 the postzygapophyseal articular facets are mainly laterally facing, with only a slight ventral orientation [9]. This orientation of the postzygapophyseal articular facets would have mostly prevented lateral movement between the vertebrae in that region of the axial skeleton, which would probably correspond to the posterior end of the neck. By contrast, in vertebra 4 the articular facet of the preserved left postzygapophysis faces ventrolaterally, which would have allowed both lateral and dorsoventral movements among the vertebrae of this region, probably corresponding to the anterior end of the trunk. In vertebrae 1, 2 and 4 the postzygapophyses extend posteriorly beyond the posterior end of the centrum, but the condition cannot be assessed in vertebrae 3 and 5. The postzygapophyses are separated from one another along the posterior midline by a deep vertical furrow that does not extend dorsally along the neural spine.

The neural arches of vertebrae 1, 2 and 4 do not possess the depressions on both sides at the base of the neural spine (Fig. 10: d) that are usually observed in varanopid “pelycosaurs” [172], [185], [186], araeoscelidians [128], [131] and some archosauromorphs (e.g. *Protorosaurus speneri*, BSPG 1995 I 5; *Mesosuchus browni*, SAM-PK-6046; *Prolacerta broomi*, BP/1/2675; *Proterosuchus fergusi*, GHG 231). The bases of the neural spines are anteroposteriorly long, extending from the base of the prezygapophyses up to a point just anterior to the posterior ends of the postzygapophyses. The preserved regions of the neural spines of vertebrae 1 and 2 suggest that the anterior portion of the anterior margin possessed a low angle to the long axis of the vertebra up to a point approximately level with the mid-length of the centrum. Beyond this point, the anterior margin became sharply upturned to form the neural spine (Fig. 10: ns). Unfortunately, the total height of the neural spine and the morphology of its distal end cannot be assessed in any of the preserved vertebrae.

The microstructure of the vertebrae can be observed due to breakages and damaged surfaces and its pattern varies in different regions of the axial elements. The articular face of the centrum possesses multiple layers of bone laminae distributed in a concentric pattern that follows the outline of the notochordal canal. These bone layers may represent successive sequences of ossification of the vertebral centrum along the internal surface of the notochordal canal, implying at least partial reabsorption of the notochord during ontogeny. Within the external borders of the centrum and along the neural arch a typical trabecular bone microstructure is visible.

The distal half of a right **humerus** is preserved, and has a well-preserved external bone surface (Fig. 12). The preserved portion of the shaft is roughly oval in cross-section, being slightly dorsoventrally deeper than anteroposteriorly wide (Table 6). The outline of the shaft in cross-section is asymmetric, with convex ventral, dorsal and anterior margins. The posterior margin is slightly sigmoid due to the presence of a posterior depression extending proximodistally along the shaft that is ventrally bounded by a thick ridge (Fig. 12: pvr). This ridge rises from the central portion of the ventral surface of the shaft and extends more posteriorly towards its proximal end. It becomes thicker towards the proximal end of the bone; distally, the ridge does not reach the distal end of the humerus but fades out on the ventral surface. The posteroventral ridge observed in the humerus of *Aenigmastropheus parringtoni* does not appear to be homologous with that of basal synapsids (e.g. dicynodonts [151]), which connects the deltopectoral crest with the entepicondyle, because in basal synapsids the ridge is directed anteriorly towards its proximal end. A similar ridge is not present in basal diapsids (e.g. *Petrolacosaurus kansensis* [131]; *Araeoscelis gracilis*, MCZ 4383; *Youngina capensis*, BP/1/3859; *Protorosaurus speneri*, BSPG 1995 I 514; *Prolacerta broomi*, BP/1/2675). This ridge is identified here as autapomorphic for *Aenigmastropheus parringtoni*.

The distal end of the humerus of *Aenigmastropheus parringtoni* is strongly anteroposteriorly expanded, being around 3.6 times wider than the proximal end of the shaft at the point at which it is broken, resembling the condition in several amniotes (e.g. *Barasaurus besairiei* [187]: fig. 3d; *Millerosaurus pricei* [129]; *Varanosaurus acutirostris*, *Dimetrodon kempae* [95]; *Dicynodontoides* spp. [151]; *Captorhinus aguti* [188]; *Araeoscelis gracilis*, MCZ 4383; *Boreopricea funerea* [105]; *Trilophosaurus buettneri* [44]). In addition, the most proximal portion of the preserved shaft indicates that the bone was still tapering proximally and the ratio of the anteroposterior width of the distal end to the minimum shaft width would have been even greater than currently preserved. By contrast, the dorsoventral thickness of the distal end of the bone is less than that of the shaft.

The distal end of the humerus possesses four well-developed and distinct distal articular condyles, which represent the entepicondyle, ectepicondyle, capitellum (radial condyle) and trochlea (ulnar condyle) (Fig. 12: ca, ect, ent, tr). The presence of four well-developed distal articular condyles resembles the condition in several basal amniotes (e.g. parareptiles [129], [187]; basal synapsids [95], [151], [189]; captorhinids [188]; basal diapsids [190]) and the basal archosauromorph *Trilophosaurus buettneri* [44]. *Protorosaurus speneri* possesses at least three distinct distal condyles, which as preserved are considerably less well developed those of *Aenigmastropheus parringtoni*. Nevertheless, it is likely that the degree of development of the distal humeral condyles of *Protorosaurus speneri* is underestimated due to the strong compression that specimens suffered during fossilization (e.g. BSPG 1995 I 5, BSPG AS VII 1207). In other archosauromorphs only the ulnar and radial condyles are distinctly developed (e.g. *Boreopricea funerea* [105]; *Malutinisuchus gratus* [191]; *Tanystropheus longobardicus* [111]; *Mesosuchus browni* [4]; *Proterosuchus fergusi* [90]; *Erythrosuchus africanus* [113]; *Euparkeria capensis* [192]).

The surfaces of the distal articular condyles are porous and covered by low striations, indicating that they were probably covered by hyaline cartilage that participated in a synovial elbow joint. The ventral surface of the humerus, proximal to the distal condyles, possesses a complex topography. Two ridge-like convexities extend from the shaft in an inverted Y-shaped pattern. The thinner convexity is anterodistally directed and contacts the base of the ectepicondyle, whereas the thicker convexity is posterodistally directed and almost reaches the base of the entepicondyle. Both convexities and the capitellum and trochlea define a sub-triangular depressed area (Fig. 12: d), the depth of which appears to be exaggerated by damage and partial collapse of the cortical bone. This ventral depression would have housed the attachment area for the antebrachial ligaments (see Angielczyk et al. [151]). Both ectepicondylar and entepicondylar foramina are absent from the distal end of the humerus of *Aenigmastropheus parringtoni*. Either both foramina, or the entepicondylar foramen alone, occur widely among amniotes (e.g. parareptiles [129], [187]; basal synapsids [95], [151], [189]; captorhinids [188]; basal diapsids [138]). By contrast, the absence of both foramina from the humerus of *Aenigmastropheus parringtoni* is a condition shared with the enigmatic neodiapsid *Helveticosaurus zollingeri* [133] and almost all archosauromorphs (e.g. *Mesosuchus browni* [4]; *Hyperodapedon gordoni* [193]; *Prolacerta broomi*, BP/1/2675; *Tanystropheus longobardicus* [111]; *Macrocnemus bassanii*, PIMUZ T4355; *Protorosaurus speneri* [21]; *Boreopricea funerea* [105]; *Trilophosaurus buettneri* [44]; *Proterosuchus fergusi* [90]; *Erythrosuchus africanus* [113]; *Euparkeria capensis*, SAM-PK-5867), with the exception of the putative protorosaurs *Czatkowiella harae*, which possesses an entepicondylar foramen [46], and *Jesairosaurus lehmani*, which possesses an ectepicondylar foramen (ZAR 09; [74]).

The anterior margin of the distal end of the humerus, above the ectepicondyle, has a prominent supinator ridge or ectepicondylar flange (Fig. 12: ecf), which was likely the area of origin of the *M. supinator* (e.g. [193]). The posterior margin of the distal end of the bone is not completely preserved, but the available portion is very thin and suggests that only a small portion of bone has been lost. This posterior margin is folded ventrally, and delimits together with the thicker of the ventral convexities a proximodistally-extending concave depression that terminates a substantial distance from the base of the entepicondyle. The dorsal surface of the distal end of the humerus is mostly occupied by a large and sub-triangular depression that would have borne the origin of the *M. triceps humeralis medialis* (see Angielczyk et al. [151]). The depth of the central and proximal areas of this depression is exaggerated by damage with collapse of cortical bone. A raised shelf of bone that is continuous with the ectepicondyle delimits the anterior border of this dorsal depression and possesses a slightly rugose dorsal surface that would have housed the area for origin of the antebrachial extensor muscles (see Angielczyk et al. [151]). The posterior border of the depression is bounded by a faint posterodistally extending convexity that is continuous with the entepicondyle. The surface of the bone posterior to this convexity is slightly rugose, representing the area of origin of the antebrachial flexor muscles (see Angielczyk et al. [151]).

The ectepicondyle is the smallest of the distal condyles and is restricted to a rounded structure with a dorsoventrally oriented main axis. The anterior surface of the ectepicondyle possesses a shallow teardrop-shaped depression that corresponds to the ectepicondylar groove [9] for the passage of the radial nerve [95] (Fig. 12: ecg). This feature is widely distributed among amniotes (e.g. *Apsisaurus witteri* [73]; *Varanops brevirostris* [172]; *Dimetrodon* sp., SAM-PK-K8670; *Youngina capensis*, BP/1/3859; *Prolacerta broomi*, BP/1/2675; *Erythrosuchus africanus* [113]) and the ectepicondylar groove of *Aenigmastropheus parringtoni* is considerably less well-developed than in some basal archosauromorphs (e.g. *Tanystropheus conspicuous* [111]; *Trilophosaurus buettneri* [44]: figs. 66–68; *Otischalkia elderae* [194]), in which the passage is represented by a deep notch.

The capitellum (Fig. 12: ca), which articulated with the radius, is a ball-shaped, proximoventrally-projecting condyle. The trochlea is the widest and deepest of the condyles of the distal end (Fig. 12: tr). It possesses a slightly convex ventral surface and a strongly convex dorsal surface that articulated with the U-shaped proximal articular surface of the ulna. In distal view, the trochlea has a comma-shaped outline, with a concavity on its posterodorsal margin. The entepicondyle (Fig. 12: ent) is smaller than the capitellum and trochlea, but larger than the ectepicondyle. It has an overall morphology that is very similar to that of the trochlea and its convex surface is dorsally oriented. The posterior surface of the entepicondyle possesses a small and deep oval concavity.

A fragment from the shaft of a long bone is interpreted as part of a probable left humerus (Fig. 14A, E, I, M, P, T). One of its ends is roughly sub-circular in cross-section and the opposite end is strongly compressed. The compressed end appears to have collapsed cortical bone on both of its main surfaces; it can thus be inferred to have had an originally sub-triangular cross-section. The size and shape of the end with a sub-circular cross-section is very similar to that of the shaft of the partial right humerus. Although this fragment of bone could belong to part of the left humeral shaft, it might also represent a fragment of tibial or femoral shaft.

The proximal end of the right **ulna** is preserved (Fig. 13). It has a well-preserved external bone surface, but the cortical bone on the posterior surface of the element distal to the olecranon process has collapsed, probably exaggerating the anteroposterior compression of the shaft (Fig. 13: ccb). This collapse is also evident in the constriction of the medullary space of the bone on the broken cross-section of the shaft (Fig. 13G). The ulna has a very large olecranon process (Fig. 13: ol), resembling the condition of some basal synapsids (e.g. *Ophiacodon navajovicus*, *Edaphosaurus boanerges* [95]; *Dinodontosaurus turpior* [189]), basal sauropsids (e.g. *Captorhinus aguti* [183]; *Thuringothyris mahlendorffae* [195]), the basal archosauromorphs *Protorosaurus speneri* [21] and *Trilophosaurus buettneri* [44], and several archosaurs (e.g. *Typothorax coccinarum* [196]; *Fasolasuchus tenax*, PVL 3850; *Saturnalia tupiniqium*, MCP 3845-PV, [197]; *Eodromaeus murphi*, PVSJ 560). By contrast, the olecranon process is poorly developed or absent in varanopid synapsids [172], “younginiforms” (e.g. *Youngina capensis* [64]; *Acerosodontosaurus piveteaui* [70]), the neodiapsid *Helveticosaurus zollingeri* (PIMUZ T4352), and most basal archosauromorphs (e.g. *Prolacerta broomi* [64]; *Tanystropheus longobardicus*, PIMUZ T2817; *Macrocnemus bassanii*, PIMUZ T4822; *Mesosuchus browni* [4]).

The olecranon process of *Aenigmastropheus parringtoni* tapers gradually towards its apex, contrasting with that of at least some “pelycosaurian” synapsids, in which the process possesses an anteroposterior expansion towards its apex (e.g. *Dimetrodon* sp., SAM-PK-K8670; *Ophiacodon* sp., MCZ 1426). There are no traces of sutures in the olecranon of *Aenigmastropheus parringtoni*; thus, it seems that this structure formed a single ossification with the ulna. By contrast, in the basal archosauromorph *Protorosaurus speneri* the olecranon is developed as a separate ossification from the rest of the ulna [21].

The proximal bone surface of the olecranon process is damaged. The olecranon process curves ventrally in order to form a U-shaped, large, oval and deeply concave ventral articular facet for the reception of the humeral trochlea (Fig. 13: has). The surface of this facet is porous and covered by faint striations, indicating that it was covered by hyaline cartilage in life. A thick lip bounds the anterior margin of the articular surface (Fig. 13: li). Dorsal to this lip, the olecranon has a concave longitudinal depression that extends distally along the anterior surface of the ulnar shaft (Fig. 13: d). The dorsal surface of the olecranon process is strongly convex and possesses a series of roughly longitudinal striations that represent the scars of the area of insertion of the *Mm. triceps* (Fig. 13: sts). The posterior surface of the olecranon possesses a longitudinal furrow, but this seems to be an artifact resulting from the collapse of the cortical bone. A lip also bounds the posterodistal border of the articular surface, but this lip is considerably lower than the anterior one. The ulnar shaft is strongly anteroposteriorly compressed, and the anterior and posterior longitudinal depressions (although the posterior depression has been exaggerated by damage) give the shaft a figure-of-eight-shaped cross-section at the point at which it is broken.

Parrington [9] included the end of a bone with a planar articular surface among the indeterminate elements of UMZC T836, and this is here identified as a possible **radius** (Fig. 14B, F, J, N, Q, U). Although one of the borders of the bone has broken away, the preserved portion of the shaft suggests that it was oval in cross section when complete. The bone clearly does not belong to a humerus, femur or tibia because it is proportionally too small. It does not appear to represent the distal end of an ulna because distal ulnae are usually considerably more compressed in amniotes. However, the morphology of this bone matches to that of a distal radius, particularly in possessing an expanded medial border, an approximately planar distal articular surface, and well-developed scars for probable ligament attachments. Indeed, the morphology of the distal articular surface in distal view is very similar to and matches that of the right radius of some amniotes (e.g. *Captorhinus aguti* [188]), with both tapering and more rounded borders. Nevertheless, the identity of this fragmentary bone cannot be established with certainty, nor can it be determined to which side it belonged.

Parrington [9] also considered two other fragments of bone within UMZC T836 as indeterminate elements. One of the elements is a flattened and plate-like element (Fig. 14D, H, L, S). Four distinct borders can be recognized when the element is viewed perpendicular to its main plane. Two of these borders are broken margins, whereas the other two are damaged but maintain their overall shape. A short convex margin possesses a sub-triangular concavity that likely formed an articular area. A straight margin extends away from this short convex margin, and gradually increases in thickness, forming a low rounded tuberosity. One of the main surfaces of the bone is convex and the other one concave. This bone might represent part of a thickened cranial bone or a pectoral/pelvic girdle element.

A small, approximately pyramidal bone is also included among the indeterminate bones listed by Parrington [9] (Fig. 14C, G, K, O, R, V). The bone is comma-shaped when it is viewed perpendicular to its main plane, but one of the tapering ends of the element in this view is broken away. Thus, the bone would likely have had an “L”-shaped morphology when it was complete. One of the main surfaces of the element is planar, whereas the opposite surface bears a high and asymmetrically placed keel formed by two concave surfaces, one deeper than the other. The textures of the planar and the deepest concave surfaces suggest that they were articular surfaces. The remaining shallow concavity appears to be a non-articular surface. The morphology of this bone does not agree with that of a proximal end of a metacarpal or metatarsal because it does not have any process with a circular or oval cross section that could represent a shaft and the positions of the articular surfaces do not match the morphology expected for a metapodial. The size of the bone is very large relative to that expected for a carpal or distal tarsal bone. As such, we are unable to provide an identification for this element.

#### Histological description

Two thin section slices were made of the fragment of shaft of the possible left humerus (Fig. 14A). If this bone does not actually represent a humerus, it would instead represent another limb bone with the same kind of preservation as the remaining elements of UMZC T836. As described above, the cross-section of the bone is ovoid to triangular-shaped, but part of the outer cortex is damaged in one quadrant (diameter ranging from 9.1 to 10.2 mm, the latter value is based on reconstructed cortex) (Fig. 15A, B). The thickness of the periosteal cortex ranges between 1.8 and 4.0 mm. The section reveals a circular medullary cavity (4.3 to 4.7 mm in diameter), which is placed slightly off center. Few bone trabeculae are present deep within the cortex, and are present mainly in the quadrant where the cortical bone is thickest. Otherwise the interior of the medulla is devoid of trabeculae.

The cortex consists of fibrolamellar bone in the deepest parts, with a woven bone matrix that is vascularized by circumferentially arranged longitudinal primary osteons (Fig. 15C, D). In the middle part of the cortex, the woven-fibered matrix is substituted to a large degree by parallel-fibered bone in the middle parts of the cortex, although a few interspaced, thin layers of woven bone do occur. Although longitudinally arranged primary osteons constitute the majority of the vascular system, reticular patterns are locally developed. The outer part of the cortex is composed of lamellar-zonal bone, which is weakly vascularized by scattered primary osteons and more radially oriented short simple vascular canals (Fig. 15C–F). In this part of the cortex, eight growth cycles are visible with the fourth to eighth growth mark (i.e. lines of arrested growth, LAG) being closely spaced just below the external bone surface (Fig. 15F: white arrows). The first (inner) three growth marks are less conspicuous and represent annuli instead of LAGs. Locally, the outermost cortical layer does not show growth marks.

In the areas of the bone where no trabecular bone has been developed, a secondary endosteal layer of lamellar bone lines the marrow cavity. The bone trabeculae are also mostly secondary in nature, consisting of lamellar bone, with only a few interstitial areas of primary bone matrix being preserved deep within some of the thicker trabeculae (Fig. 15E). Towards the more external parts of the cortex, the cavities decrease in size and only few scattered secondary osteons are present in the surrounding primary bone matrix. Sharpey's fibers are rare in the section and restricted to few locations in the outermost parts of the cortex, indicating few localized areas of tendinous muscle attachment.

## Discussion

### Phylogenetic position of *Aenigmastropheus parringtoni*

The position of *Aenigmastropheus parringtoni* within Archosauromorpha is supported by three unambiguous synapomorphies in our analysis, the presence of parallelogram-shaped centra, in which the anterior articular surface is placed dorsal to the posterior surface, in the cervico-dorsal vertebrae in lateral view (character 174, state 1, CI = 0.5000; RI = 0.8750; Fig. 16), prezygodiapophyseal lamina present in posterior cervical and anterior–middle dorsal vertebrae (character 182, state 1, CI = 0.2500; RI = 0.6667; Fig. 16), and the absence of an entepicondylar foramen in the distal end of the humerus (character 90, state 1, CI = 1.0000; RI = 1.0000; Fig. 17A–C).

An entepicondylar foramen is absent in almost all archosauromorphs, with exception of *Czatkowiella harae* [46]. However, the nature of the multitaxonomic bonebed that yielded *Czatkowiella harae* suggests that caution may be necessary in referring humeri with this morphology to *Czatkowiella harae* (the holotype of which is based on a right maxilla) rather than to a non-archosauromorph diapsid.

The presence of parallelogram-shaped centra in the cervico-dorsal vertebrae in lateral view is present in all the archosauromorphs sampled in this analysis (i.e. *Aenigmastropheus parringtoni*; *Trilophosaurus buettneri* [44]; *Prolacerta broomi*, BP/1/2675; *Proterosuchus fergusi*, NM QR 1484; *Erythrosuchus africanus*, NHMUK R3592; *Tanystropheus longobardicus*, PIMUZ T2817T, SMNS 54628, 54630; *Euparkeria capensis*, SAM-PK-5867; *Protorosaurus speneri*, BSPG 1995 I 5; *Macrocnemus bassanii*, PIMUZ T4355; *Eorasaurus olsoni*, PIN 156/108, 109) and was convergently acquired by the varanopid synapsid *Apsisaurus witteri* ([73]: fig. 6a).

A prezygodiapophyseal lamina in posterior cervical and anterior–middle dorsal vertebrae is present in *Aenigmastropheus parringtoni* and several archosauromorphs, including *Trilophosaurus buettneri* (S. Nesbitt pers. comm. 2013; MDE pers. obs. USNM mounted skeleton), *Prolacerta broomi* (BP/1/2675), *Erythrosuchus africanus* (NHMUK R3592), *Tanystropheus longobardicus* (PIMUZ T2817T), *Euparkeria capensis* (UMZC T921), *Protorosaurus speneri* (BSPG 1995 I 5), *Macrocnemus bassanii* (PIMUZ T4822) and *Eorasaurus olsoni* (PIN 156/108–110). This feature is convergently present in the varanopid synapsid *Apsisaurus witteri* ([73]: fig. 6a) and within Archosauromorpha it is interpreted as secondarily lost in rhynchosaurs (*Noteosuchus colletti*, AM 3591; *Mesosuchus browni*, SAM-PK-6046; *Howesia browni*, SAM-PK-5886) and *Proterosuchus fergusi* (NM QR 1484, SAM-PK-K140).

The position of *Aenigmastropheus parringtoni* within Archosauromorpha is better supported by two character states that optimize as synapomorphies of Protorosauria: dorsal vertebrae with anterior centrodiapophyseal lamina or paradiapophyseal lamina (character 180, state 1, CI = 0.1667; RI = 0.6154; Fig. 16), and with posterior centrodiapophyseal lamina (character 181, state 1, CI = 0.5000; RI = 0.8000; Fig. 16). The presence of this suite of laminae in the dorsal vertebrae is reconstructed as independently acquired in Protorosauria and in the *Prolacerta*+Archosauriformes clade because these laminae are absent in *Trilophosaurus* and rhynchosaurs. However, this optimization should be considered as a preliminary result because several archosauromorphs (e.g. *Pamelaria dolichotrachela* [198]; *Spinosuchus caseanus* [112]; *Augustaburiania vatagini* [108]) and the enigmatic neodiapsid *Helveticosaurus zollingeri* also possess laminae in the neural arches of their dorsal vertebrae and were not included in this analysis (Fig. 16E). For example, the position of *Helveticosaurus zollingeri* among neodiapsids may affect the optimization of the presence of laminae in the dorsal vertebra and the absence of an ectepicondylar foramen in the distal end of the humerus. Some authors favored a position of *Helveticosaurus zollingeri* as closely related to or included within sauropterygians, but outside Sauria [39], [199], [200], or nested within lepidosauromorphs as a sauropterygian [32]. If either of these positions is correct, and depending on the phylogenetic interrelationships within Lepidosauromorpha, the optimization of these character states could turn out to be an ambiguous synapomorphy of Sauria or an independent acquisition in archosauromorphs and *Helveticosaurus zollingeri*. By contrast, Rieppel [133] concluded that *Helveticosaurus zollingeri* should be considered a basal member of Archosauromorpha. In this hypothesis, these character states will likely be optimized as synapomorphies of Archosauromorpha and *Aenigmastropheus parringtoni* would probably be recovered as the most basal archosauromorph. In any case, the combination of the presence of strongly developed laminae in the dorsal vertebrae and the absence of an ectepicondylar foramen in the distal end of humerus is congruent with archosauromorph affinities for *Aenigmastropheus parringtoni*. The exact optimization of these characters will remain an open question until the development of a more robust consensus for diapsid interrelationships, a topic beyond the scope of this paper.

Among archosauromorphs, the position of *Aenigmastropheus parringtoni* as more closely related to *Protorosaurus speneri* than to any other member of the group is supported by the following synapomorphies: anterior and mid-dorsal vertebrae with zygapophyses placed close to one another medially (character 185, state 1, CI = 0.2500; RI = 0.6250; Fig. 18A–C), and ulna with a strongly developed olecranon process, being higher than its transverse depth at its base (character 94, state 2, CI = 0.2500; RI = 0.7143; Fig. 17D–F). The absence of zygapophyses that are mainly sagittally oriented in the dorsal vertebrae of *Tanystropheus longobardicus* (SMNS 54630), *Macrocnemus bassanii* (PIMUZ T4822), *Trilophosaurus buettneri* [44] and *Mesosuchus browni* (SAM-PK-6046) results in the optimization of this character state as independently acquired in the *Aenigmastropheus*+*Protorosaurus* and *Prolacerta*+Archosauriformes clades and in some ophiacodontid and varanopid “pelycosaurs”. A strongly developed olecranon process occurs rarely among “younginiforms” and basal archosauromorphs (e.g. tanystropheids, rhynchosaurs, *Prolacerta* and basal archosauriforms). In *Trilophosaurus* the olecranon process is relatively well developed but to a lesser degree than in *Aenigmastropheus parringtoni* and *Protorosaurus speneri*.

Under constrained topologies only one additional step is necessary to position *Aenigmastropheus parringtoni* outside Archosauromorpha and as the sister-taxon of the “pelycosaurian” synapsid *Ophiacodon* (Templeton test, p-value = 1.0000, non-significant [NS]). This result is not unexpected because of the fragmentary condition of the type specimen of *Aenigmastropheus parringtoni*, which can be scored for less than 10% of the characters of our data set, and previous authors have already highlighted similarities between the vertebrae of this taxon and those of “pelycosaurian” synapsids [144]. However, we still consider the position of *Aenigmastropheus parringtoni* within “pelycosaurian” synapsids to be unlikely because it possesses several features that are not congruent with the typical morphology of the group, such as the presence of posterior centrodiapophyseal and prezygodiapophyseal laminae and low and sub-triangular neural spines in the cervico-dorsal vertebrae, and the absence of both entepicondylar and ectepicondylar foramina on the distal end of the humerus.

Three additional steps are necessary to recover *Aenigmastropheus parringtoni* as a non-diapsid sauropsid or a very basal diapsid (Templeton test for the most basal neodiapsid hypothesis, p-value = 0.5078 [NS]). Two additional steps are necessary to position *Aenigmastropheus parringtoni* as the most basal archosauromorph (Templeton test, p-value = 0.6875 [NS]) and five for it to be positioned as the sister-taxon of Sauria (Templeton test, p-value = 0.1797 [NS]). Accordingly, although the support for the archosauromorph position of *Aenigmastropheus parringtoni* is relatively low, based on the currently available information for the species and the MPTs obtained in our phylogenetic analysis we favor the hypothesis that *Aenigmastropheus parringtoni* is a Late Permian archosauromorph.

### Phylogenetic position of *Eorasaurus olsoni*

*Eorasaurus olsoni* was included for the first time in a quantitative phylogenetic analysis and our recovery of this species within Archosauromorpha partially supports the original interpretation of Sennikov [89]. However, the position of *Eorasaurus olsoni* within Archosauriformes within our phylogenetic results differs from the phylogenetic hypothesis of Sennikov [89] that *Eorasaurus olsoni* was closely related to *Protorosaurus speneri* and, as a result, a member of Protorosauridae. The inclusion of *Eorasaurus olsoni* within Archosauriformes is supported by the presence of diapophyses in the anterior dorsal vertebrae that are wider than 65% of the length of the centrum (character 178, state 1, CI = 0.2000; RI = 0.2000; Fig. 18D–F).

The position of *Eorasaurus olsoni* as an archosauriform closer to *Erythrosuchus africanus* and *Euparkeria capensis* than to Proterosuchidae is supported by the presence of a posterior centrodiapophyseal lamina in the posterior cervical and dorsal vertebrae (character 181, state 1; Fig. 16). This character state is also present in protorosaurs (i.e. *Aenigmastropheus parringtoni*, *Protorosaurus speneri*, *Tanystropheus longobardicus*, *Macrocnemus bassanii*), but is absent in *Trilophosaurus buettneri*, rhynchosaurs, *Prolacerta broomi* and *Proterosuchus fergusi*. As a result, the present analysis optimizes independent acquisitions of a posterior centrodiapophyseal lamina for protorosaurs and the *Eorasaurus*+*Erythrosuchus+Euparkeria* clade (see above). *Eorasaurus olsoni* differs from protorosaurs in the presence of proportionally shorter cervical vertebrae and the presence of a very well developed, laterally projected diapophysis in anterior dorsal vertebrae. As a result, two extra steps are necessary to recover *Eorasaurus olsoni* within Protorosauria (Templeton test, p-value = 0.6250 [NS]). Only one additional step is required to position *Eorasaurus olsoni* as the sister-taxon of Archosauriformes (Templeton test, p-value = 1.000 [NS]), three additional steps to be placed as the sister-taxon of *Prolacerta broomi* (Templeton test, p-value = 0.2500 [NS]), four additional steps to be positioned at the base of Archosauromorpha (Templeton test, p-value = 0.2187 [NS]), and five additional steps to be found outside Sauria, being nested within Varanopidae (Templeton test, p-value = 0.1250 [NS]). As a result, the nesting of *Eorasaurus* within Sauria and Archosauromorpha seems to be quite well supported (but is not statistically significant).

The position of *Eorasaurus olsoni* as a member of Archosauriformes would imply that it is the oldest known member of the group, but the phylogenetic position recovered here for the species should be considered tentative given that the known material is highly incomplete and its phylogenetic position labile in sub-optimal trees. The position of *Eorasaurus olsoni* should be further tested in phylogenetic analyses that include wider taxonomic samples of archosauromorphs.

### Timing of the crocodile-lizard (or bird-lizard) divergence and recommendations for molecular calibrations

**Figure 19:**
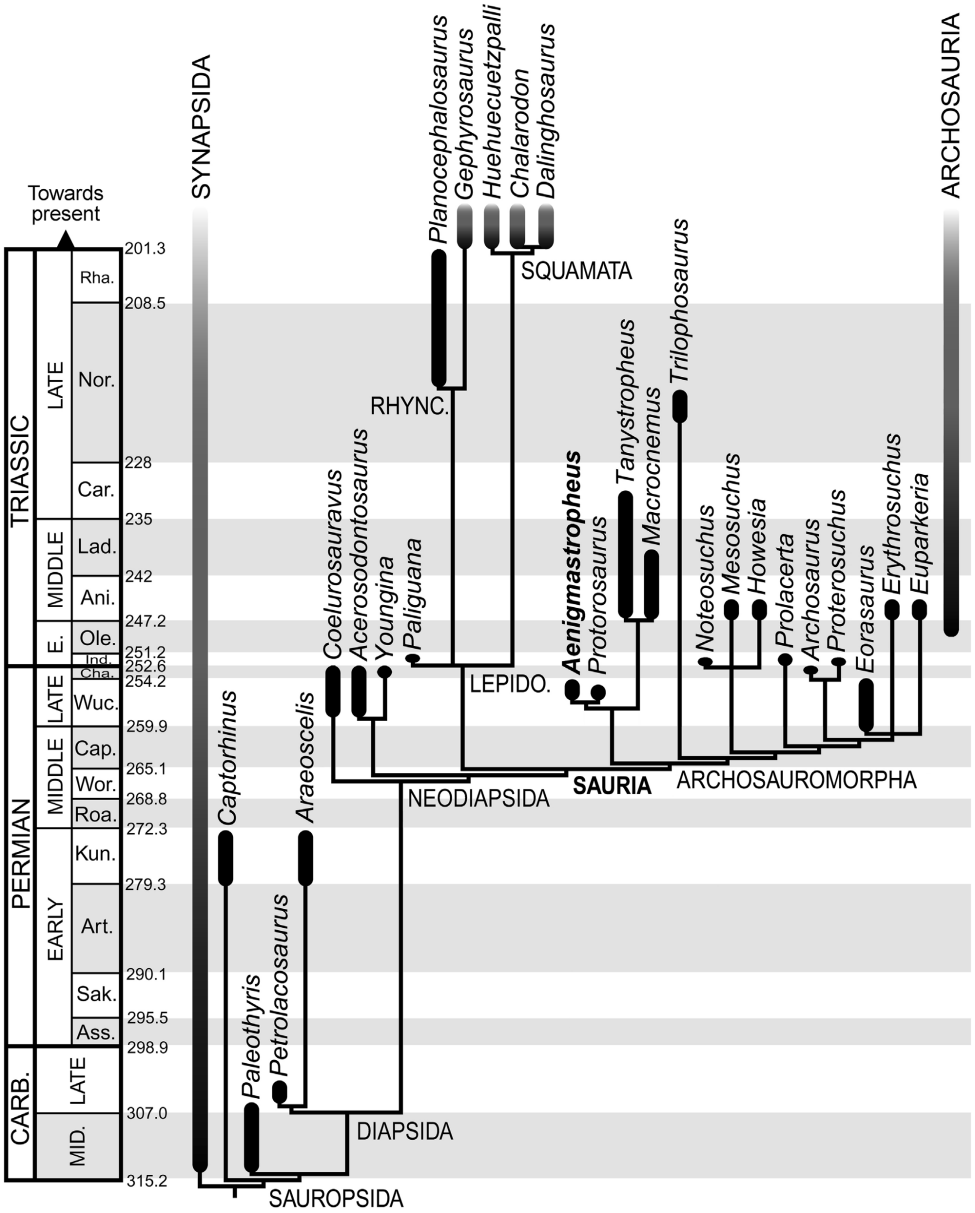
Time-calibrated cladogram showing the basal saurian interrelationships recovered here. Geological timescale after Gradstein et al. [157].

Our revision of the Permian saurian record indicates that only four species can be considered as well-supported pre-Mesozoic members of the group: the approximately contemporaneous early–middle Late Permian *Protorosaurus speneri*, *Aenigmastropheus parringtoni* and *Eorasaurus olsoni* and the latest Permian *Archosaurus rossicus* (Table 7). Other supposed saurian taxa are identified as more basal diapsid forms (e.g. *Saurosternon*, *Youngina*) or as indeterminate reptiliomorphs (e.g. BP/1/4220), or as of Early Triassic or indeterminate Late Permian–Early Triassic age. All the Permian saurian taxa recognized here can be assigned to Archosauromorpha and, as a result, there is no fossil record of Lepidosauromorpha prior to the Early Triassic (*Paliguana whitei*) (Fig. 19). The radioisotopic estimate for the age of *Protorosaurus* establishes a minimum divergence time for the crocodile-lizard split (origin of Sauria; equivalent to the bird-lizard split).

**Table 7:** List of specimens/species previously considered as pre-Mesozoic saurians and their age and current taxonomic assignment.

Specimen/Taxon	Age	Occurrence	Reported taxonomic assignment	Revised taxonomic assignment
***Aenigmastropheus parringtoni***	**middle–late Wuchiapingian, middle Late Permian**	**Tanzania**	**“Proterosuchia” (Charig & Sues ** [**127**] **)**	**Archosauromorpha, ?Protorosauria**
***Archosaurus rossicus***	**Changhsingian, latest Permian**	**Russia**	**Proterosuchidae (Tatarinov ** [**88**] **; Nesbitt ** [**59**] **)**	**Archosauriformes, Proterosuchidae**
BP/1/4220	Late Permian–Early Triassic	South Africa	?*Proterosuchus* sp. (Cruickshank [90])	Reptiliomorpha
Buena Vista Fm specimens	Late Permian–Early Triassic	Uruguay	Synapsida (Piñeiro et al. [140])	Amniota
***Eorasaurus olsoni***	**late Capitanian– Wuchiapingian, late Middle Permian–early Late Permian**	**Russia**	**Archosauromorpha, Protorosauria (Sennikov ** [**89**] **)**	**Archosauromorpha, ?Archosauriformes**
*Lanthanolania ivakhnenkoi*	late Wordian, middle Middle Permian	Russia	Lepidosauromorpha/sister-taxon of Sauria (Modesto & Reisz [15]); non-saurian Neodiapsida (Reisz et al. [75])	Non-saurian Neodiapsida
*Lacertulus bipes*	Late Permian–Early Triassic	South Africa	Lepidosauromorpha (Carroll & Thompson [80]); non-squamate Diapsida (Estes [86]; Evans [3])	Non-squamate Diapsida
*Palaeagama vielhaueri*	Late Permian–Early Triassic	South Africa	Lepidosauromorpha (Carroll [10]; Gauthier et al. [57]); non-saurian Neodiapsida (Müller [32])	Non-saurian Neodiapsida
*Paliguana whitei*	Early Triassic	South Africa	Lepidosauromorpha (Carroll [10], [93]; Evans [56])	Lepidosauromorpha
***Protorosaurus speneri***	**middle Wuchiapingian, middle Late Permian**	**Germany and England**	**Archosauromorpha Protorosauria (Benton ** [**52**] **; Gottman-Quesada & Sander ** [**21**] **)**	**Archosauromorpha, Protorosauria**
*Saurosternon bainii*	Late Permian	South Africa	Lepidosauromorpha (Carroll [10]; Gauthier et al. [57]); non-saurian Neodiapsida (Müller [32])	Non-saurian Neodiapsida

Permian specimens/species currently supported as saurian are highlighted in bold font.

Following the explicit five-point protocol for fossil calibrations proposed by Parham et al. [201], we make the following recommendations for molecular biologists wishing to use fossil data to calibrate the crocodile-lizard (or bird-lizard) split: (1) the split can be calibrated on the basis of the voucher specimen NHMW 1943 I 4, an almost complete skeleton missing the skull, lectotype of *Protorosaurus speneri* Meyer 1830 [98] (see Gottmann-Quesada & Sander [21] for designation of lectotype material); (2) *Protorosaurus speneri* is universally considered and strongly supported as a non-archosaurian member of Archosauromorpha upon the basis of the explicit morphological phylogenetic analyses conducted here, and many other studies [4], [21], [52], [56], [72], [73], [74], [105], [106]; (3) results of morphological phylogenetic analyses for major saurian relationships are generally consistent with molecular analyses, with the exception of the highly controversial phylogenetic position of turtles (e.g. [26]–[39]). However, because the earliest fossil turtle remains are Late Triassic in age [202] the problematic position of turtles has no impact on the calibration proposed here; (4) NHMW 1943I4 comes from the locality Glücksbrunn in Thuringia, central Germany, and is from the Kupferschiefer (cycle Z1) of the Zechstein Group; (5) as discussed above, the Kupferschiefer is dated as 257.3±2.6 Ma (Late Permian/Lopingian: Wuchiapingian) based on a Re-Os geochronological study [101]. This age is consistent with biostratigraphic data from the conodont *Mesogondolella britannica* supporting a middle Wuchiapingian age [102], [103] (Schneider pers. comm. 2012).

These data suggest a minimum or soft calibration date for the crocodile-lizard split of 254.7 Ma (the youngest date for the Kupferschiefer suggested by geochronology). This is younger than the minimum calibration date proposed by Benton & Donoghue [24], who proposed a calibration of 259.7 Ma, based on their interpretation of the Kupferschiefer as older (Capitanian) in age, and slightly older than the 252 Ma calibration recommended by Reisz & Müller [22].

A maximum or hard calibration date for the crocodile-lizard split is more difficult, and Benton & Donoghue [24] proposed that *Apsisaurus* from the Archer City Formation of Texas, dated as Asselian (298.9±0.2 Ma to 295.5±0.4 Ma; [157]) can be used to constrain the maximum date of divergence, and therefore recommended a maximum or hard calibration date of 299.1 Ma. However, *Apsisaurus* was recently re-interpreted as a varanopid synapsid [43]. Accordingly, we propose here that the basal neodiapsid *Lanthanolania ivakhnenkoi* be used to constrain the maximum divergence time of Sauria because it is the closest of the sister taxa to Sauria [75] that is unambiguously older than the earliest known saurian fossils. *Lanthanolania* comes from the Wordian of Russia [15], [75] (268.8±0.5 Ma to 265.1±0.4 Ma; [157]) and implies a maximum calibration date of 269.3 Ma, which is around 30 Ma younger than the date previously recommended by Benton & Donoghue [24].

### Ghost lineages and archosauriform divergence

As discussed above, Archosauromorpha and Lepidosauromorpha diverged prior to 254.7–259.9 Ma (middle–late Wuchiapingian; [157], [203]) (Fig. 19). This minimum divergence date matches that of the immediate successive outgroups of Sauria, such as “younginiforms” and tangasaurids [75], implying the absence of an unambiguous ghost lineage for Sauria (Fig. 19). A middle–late Wuchiapingian age for the origin of the group is consistent with dates estimated by some recent molecular clocks, such as those of Alfaro et al. [204] of 257–292 Ma and Sanders & Lee [25] of 249.5–269.1 Ma. Conversely, the age estimated here for the origin of Sauria is considerably younger than dates estimated by some other recent molecular clock analyses that have proposed an Early Permian divergence data (e.g. 285–289 Ma by Hugall et al. [205]; 276–295 Ma by Shen et al. [38]). As a result, the ghost lineage between the origin of Sauria (and several non-saurian neodiapsids) and the first appearance of the group in the fossil record would be of 21 to 40 million years based on the latter molecular estimates. This extensive ghost lineage could be explained by gaps in the fossil record or by errors in the estimations based on molecular evidence (such as a more rapid rate of molecular evolution than those expected by the molecular models). Accordingly, although the oldest known unambiguous members of Sauria are middle Late Permian in age, it would not be surprising to find early members of the group in Middle Permian rocks. Such discoveries would result in a considerably older minimum origin time for the group, as has also recently been documented for neodiapsid sauropsids [75].

The recovery of the Russian *Eorasaurus olsoni* within Archosauriformes in our phylogenetic analysis implies that it represents the oldest member of the group, also suggesting a minimum middle Wuchiapingian divergence time for rhynchosaurians, prolacertids, proterosuchids and the *Erythrosuchus*+*Euparkeria* clade (Fig. 19). As a result, this phylogenetic position would indicate that archosauriforms are not a group that appeared immediately before the Permo-Triassic mass extinction event and, conversely, had already undergone substantial taxonomic diversification by the Late Permian, with the presence of proterosuchids and the *Eorasaurus* lineage. However, the fragmentary nature of the known specimens of *Eorasaurus olsoni* and the weak support for its position within archosauriforms (e.g. only one extra step is necessary to recover it outside Archosauriformes) suggests that the phylogenetic position recovered here for this taxon should be considered as tentative.

### Paleobiogeography

**Figure 20:**
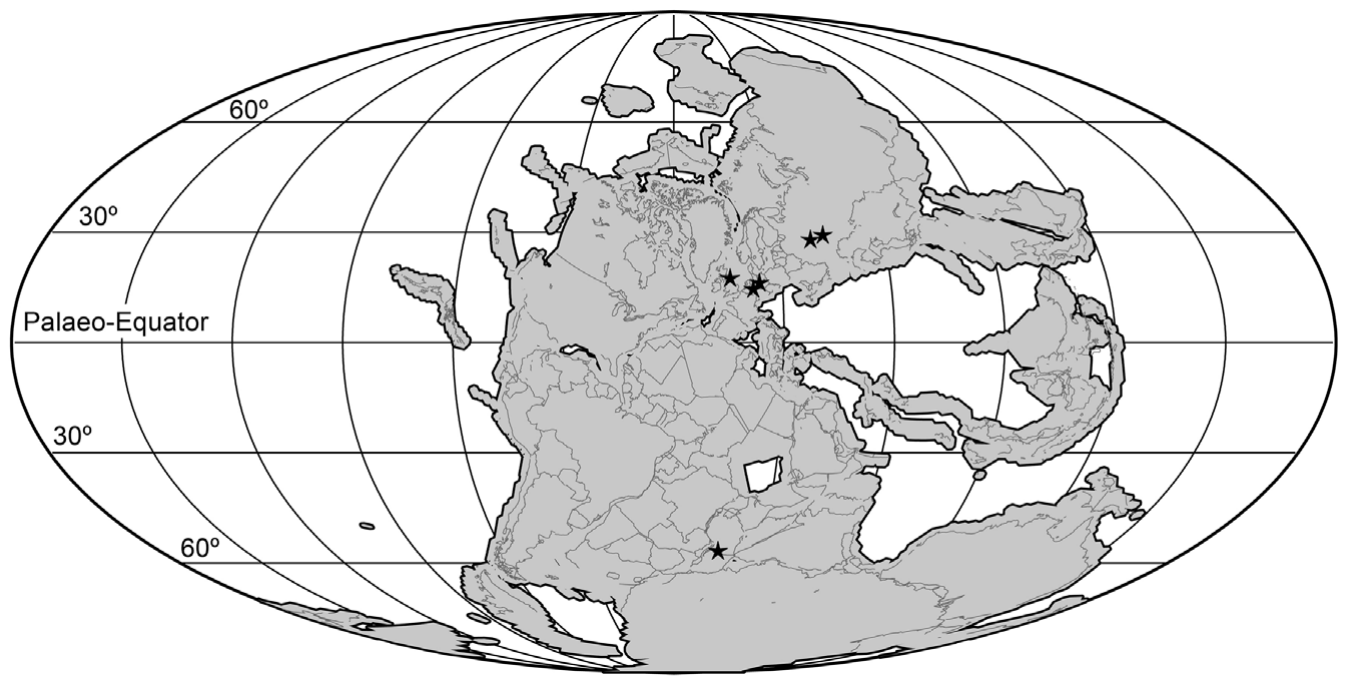
Paleobiogeographical distribution of Archosauromorpha across Pangea during the late Middle–Late Permian. Black stars indicate archosauromorph records (paleomap for 260 Ma downloaded from *Fossilworks* using data from the *Paleobiology Database*: [206]).

The presence of early archosauromorphs in the middle Upper Permian of Germany, England, Russia and Tanzania indicates a broad geographical distribution for the group during the late Paleozoic, spanning from close to the paleo-Equator (Germany) to a paleolatitude of 30°N (Russian localities) in the northern hemisphere to high paleolatitudes of 55°S (Tanzania) in southern Pangea (Fig. 20; paleomap generated using *Fossilworks* based upon data from the *Paleobiology Database*: [206]). This broad paleobiogeographic distribution during the Permian undermines previous hypotheses that proposed a dispersal event for archosauromorphs from Eurasia to southern high latitudes (southern Africa) following the Permo-Triassic mass extinction [14]. The occurrences of *Protorosaurus speneri* and *Aenigmastropheus parringtoni* imply either a northern–southern dispersal event or a wider paleobiogeographic distribution for archosauromorphs during the Late Permian, which is not currently well documented in the fossil record. In addition, previous authors have discussed the presence of endemic taxa in the Usili Formation of Tanzania (e.g. [147], [149], [155], [207]–[210]), which yielded the holotype of *Aenigmastropheus parringtoni*. The occurrence of the basal archosauromorph *Aenigmastropheus parringtoni* in the Usilli Formation contrasts with the current absence of the group in the Late Permian of the Upper Madumabisa Mudstone of Zambia and the considerably better sampled *Cistecephalus* Assemblage Zone of the South African Karoo Basin. Thus, *Aenigmastropheus parringtoni* enlarges the list of endemic taxa for the Tanzanian unit (see Angielczyk et al. [155]) and contrasts with previous hypotheses of a more homogenous Pangean fauna during the Late Permian (e.g. [211]).

### Paleobiology

The osteohistological evidence gathered from *Aenigmastropheus parringtoni* has potential implications for the understanding of the paleobiology of early archosauromorphs. The analyzed bone of *Aenigmastropheus parringtoni* belonged to an animal that exhibited high growth rates during early development, as exemplified by the short period of fibrolamellar bone formation. The steady decrease in vascularization coupled with the increase in spatial organization of the bone matrix up until the formation of lamellar-zonal bone indicates, however, that the animal was growing more slowly for most of its life. The closely spaced growth marks found in the lamellar-zonal bone of the outer part of the cortex could be interpreted as an outer circumferential layer (OCL, see Ponton et al. [212]; often referred to also as the external fundamental system [EFS]), in which case the animal could be considered to have reached skeletal maturity (Fig. 15D, E). It remains unclear though why the outermost thin layer of bone directly below the external bone surface does not show growth marks. Growth marks in this layer could be simply obscured in some areas or they could be truly absent. If the latter condition is assumed to be correct, this would argue against the presence of an OCL, as the supposed OCL would not reach the external bone surface. Instead, the closely spaced LAGs would instead indicate that the animal had a prolonged period in life (e.g. several years) as an adult in which it experienced unfavorable growth conditions (e.g. nutritional shortage, drought, disease, etc.) leading to restricted growth (Fig. 15F). The growth pattern observed in *Aenigmastropheus parringtoni* resembles those of *Prolacerta broomi* and the basal archosauriforms *Proterosuchus fergusi*, *Erythrosuchus africanus* and *Chanaresuchus bonapartei*, with rapid growth during their early development [213]. By contrast, extant lepidosauromorphs [214], [215] and the basal archosauromorphs *Trilophosaurus buettneri* and hyperodapedontid rhynchosaurs [216], [217] exhibit a slow overall growth pattern. Accordingly, the currently available osteological evidence indicates the presence of diverse growth strategies among basal members of Archosauromorpha, but further sampling of early members of the group is required to establish the optimization of growth strategies and its implications for the early evolution of archosauromorphs.

**Figure 21:**
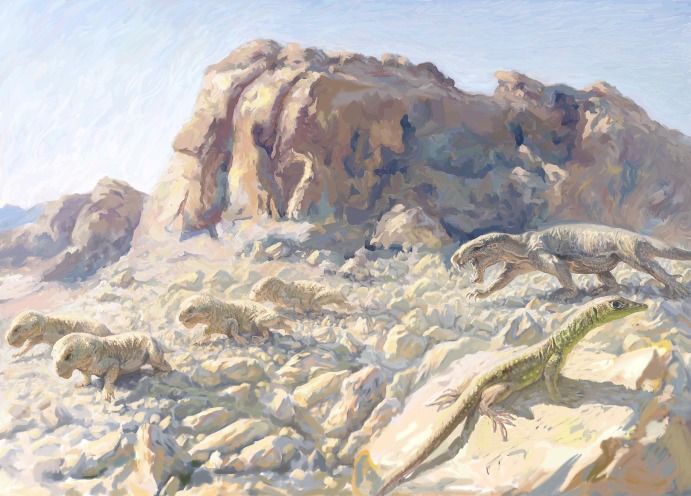
Late Permian of Tanzania at the time of deposition of the Usili Formation. Life restoration of *Aenigmastropheus parringtoni* (foreground), and herd of the dicynodont *Endothiodon* being pursued by a gorgonopsian. All these vertebrates were found together at the type locality of *Aenigmastropheus parringtoni* (locality B35). Drawing by Emilio López-Rolandi.

The early archosauromorph *Protorosaurus speneri* was interpreted as a fully terrestrial animal found in marine paleoenvironments as an allochthonous element, in which its carcasses were transported from nearby islands [21], [218]. The presence of terrestrial plant remains (i.e. ovules of a conifer) in the gut content of one *Protorosaurus speneri* specimen provides support for this hypothesis [219]. The fragmentary condition of the holotype of *Aenigmastropheus parringtoni* complicates an assessment of the paleobiology of the species. However, the strongly developed distal humeral condyles and olecranon process of the ulna suggest a fully terrestrial mode of life (Fig. 21). The sectioned probable humerus of *Aenigmastropheus parringtoni* has a relatively thick cortex (k = 0.44, represented by the ratio between the inner diameter and outer diameter of the bone; [220]) that closely resembles the condition of some bones of terrestrial basal archosauriforms, such as the humerus and tibia of *Euparkeria capensis* (k = 0.44, 48) and the fibula of *Proterosuchus fergusi* (0.37) [213]. The k value observed in *Aenigmastropheus parringtoni* is higher than those observed in aquatic and semi-aquatic animals (k<0.30) [221]–[223], supporting a terrestrial mode of life for this species and thus also for the earliest known archosauromorphs.

## Supporting Information

File S1 **Supporting information.** Appendix SI, character list corresponding to the data matrix analyzed in this paper; Appendix SII, character states modified from Reisz et al. (2010) original data matrix; Appendix SIII, TNT file of the data matrix corresponding to the data set analyzed in this paper; Appendix SIV, list of unambiguous synapomorphies in the sauropsid branch recovered in the MPT; Appendix SV, supplementary references.
(DOCX)
