# Correction: The Origin and Early Evolution of Sauria: Reassessing the Permian Saurian Fossil Record and the Timing of the Crocodile-Lizard Divergence

**DOI:** 10.1371/journal.pone.0097828

**Published:** 2014-05-13

**Authors:** The PLOS ONE Staff

The reported age of the Kupferschiefer fossil, 257.3±2.6 Ma, is incorrect. The authors have provided the corrected age of 257.3±1.6 Ma.

As a result, the recommended age of the crocodile-lizard split, 254.7 Ma, is also incorrect. The correct recommended age is 255.7 Ma. The authors also report that Archosauromorpha and Lepidosauromorpha diverged prior to 255.7-258.9 Ma rather than 254.7–259.9.

## References

[1] Ezcurra MD, Scheyer TM, Butler RJ (2014) The Origin and Early Evolution of Sauria: Reassessing the Permian Saurian Fossil Record and the Timing of the Crocodile-Lizard Divergence. PLoS ONE 9(2): e89165 doi:10.1371/journal.pone.0089165 24586565 10.1371/journal.pone.0089165 PMC3937355

