# Supplementary material for: The Origin and Early Evolution of Sauria: Reassessing the Permian Saurian Fossil Record and the Timing of the Crocodile-Lizard Divergence
Source: PLoS One. 2014 Feb 27;9(2):e89165. doi: 10.1371/journal.pone.0089165 (PMC3937355; doi:10.1371/journal.pone.0089165)
Supplement: File S1 — Supporting information. Appendix SI, character list corresponding to the data matrix analyzed in this paper; Appendix SII, character states modified from Reisz et al. (2010) original data matrix; Appendix SIII, TNT file of the data matrix corresponding to the data set analyzed in this paper; Appendix SIV, list of unambiguous synapomorphies in the sauropsid branch recovered in the MPT; Appendix SV, supplementary references. (DOCX) [file pone.0089165.s001.docx]

**Supporting information for Ezcurra et al. “The origin and early evolution of Sauria: reassessing the Permian saurian fossil record and the timing of the crocodile-lizard divergence”**

APPENDIX SI

Character list corresponding to the data matrix analyzed in this paper. Character 1–112 follow those of Reisz et al. (2010) with modifications detailed accordingly. Characters 113–218 have been added here mainly from Dilkes (1998), Müller (2004) and Senter (2004), plus some additional new characters.

(1) Teeth, tooth attachment: subthecodont (= protothecodont) (0); ankylothecodont (1); pleurodont (2); acrodont (teeth fused to jawbone in adults so that no root can be discerned; no tooth replacement) (3); thecodont (4) (Dilkes, 1998: 55; Laurin, 1991: G4; Müller, 2004: 38; modified from Reisz et al., 2010: 1). We have added the fifth state ‘thecodont’ to account for the condition present in *Erythrosuchus africanus* and *Euparkeria capensis*.

(2) Teeth, distal curvature of marginal teeth: strong (0); slight (1); absent (2) (Reisz and Dilkes, 2003: 1; Reisz et al., 2010: 2), ORDERED.

(3) Teeth, serrations on crown: absent (0); present (1) (Reisz and Dilkes, 2003: 32; Reisz et al., 2010: 3).

(4) Teeth, lateral compression of marginal dentition: only distally or nowhere (0); over two-thirds of tooth (1) (Reisz and Dilkes, 2003:1, 34; Reisz et al., 2010: 4).

(5) Snout, shape of antorbital region: broad, nasal largely dorsal element (0); narrow and tall, nasal has nearly vertical contribution to snout (1) (Reisz and Dilkes, 2003: 12; Reisz et al., 2010: 5).

(6) Snout, antorbital-postorbital ratio: postorbital part (posterior margin of orbit to caudal tip of skull) longer than antorbital part (0); antorbital part of snout (tip to anterior margin of orbit) longer but nasal shorter or equal in length to frontal (1); antorbital part of snout (tip to anterior margin of orbit) longer and nasal longer than frontal (2) (Rieppel et al., 1999 in part; Reisz and Dilkes, 2003: 50 in part; Müller, 2004: 4, 8; modified from Reisz et al., 2010: 6, 22), ORDERED.

(7) Snout, proportions: width > height (0); width < height (1). (Reisz and Dilkes, 2003: 53; Reisz et al., 2010: 7)

(8) Premaxilla, number of teeth: ≥ 5 (0); 2 to 4 (1); 0 (2) (Laurin, 1991: G1; Reisz and Dilkes, 2003: 41; Müller, 2004: 152; Reisz et al., 2010: 8), ORDERED.

(9) Premaxilla, anterior process: absent (0); present (1) (Reisz and Dilkes, 2003: 51; Reisz et al., 2010: 9).

(10) Premaxilla, downturned alveolar margin: no (0); slightly (1); strongly (2) (Dilkes, 1998: 6; Reisz et al., 2010: 10), ORDERED.

(11) Premaxilla, narial shelf: more or less sharp edge between lateral (= sculptured, if sculpture is present) surface of skull and ventral wall of naris (0); rounded ventral narial shelf that transitions smoothly into ventral edge of skull (1) (Reisz and Dilkes, 2003: 2; Reisz et al., 2010: 11).

(12) Premaxilla, postnarial process (= dorsolateral process): absent (0); small, extends slightly behind naris but maxilla forms most of the border of the external naris (1); well-developed, forms more of the border of the external naris than the maxilla or excludes the maxilla from participation in the external naris (2) (Laurin, 1991: F1; modified from Reisz et al., 2010: 12), ORDERED.

(13) Maxilla, dorsal process: absent (0); starts just behind external naris, extends to level of dorsal narial margin (1); spike-like, just in front of orbit, overlies lacrimal (2); massive, pillar-like, extends above narial margin (3) (Reisz and Dilkes, 2003: 5; Reisz et al., 2010: 13).

(14) Maxilla, contact with prefrontal: absent (0); present (1) (Reisz and Dilkes, 2003: 6; Müller, 2004: 179; Reisz et al., 2010: 14).

(15) Maxilla, tooth number: 25 or fewer (0); 26 or more (1) (Reisz and Dilkes, 2003: 28; Reisz et al., 2010: 15).

(16) Maxilla, caniniform region: region absent (0); region present (1); one or two caniniform teeth present (2) (Laurin, 1991: B1, and Reisz and Dilkes, 2003: 39; Reisz et al., 2010: 16), ORDERED.

(17) Maxilla, dorsal of caniniform tooth or region: flat (0); swollen (1); well-defined buttress (2) (Reisz and Dilkes, 2003: 10; Reisz et al., 2010: 17). This character is scored as inapplicable in taxa that have state 16(0), ORDERED.

(18) Septomaxilla, shape: pillar-like (0); curled sheet (1); absent (2) (Dilkes, 1998: 14; Müller, 2004: 87; Reisz et al., 2010: 18). We deleted the “flat” state and added an additional character-state in order to include the condition of taxa in which the septomaxilla is absent (e.g. *Erythrosuchus*; Gower, 2003), ORDERED.

(19) Septomaxilla, lateral sheet-like exposure: absent (0); present (1) (Reisz and Dilkes, 2003: 42; Reisz et al., 2010: 19). This character is scored as inapplicable in taxa that lack a septomaxilla, character-state 18(3).

(20) External naris, position: marginal (minimal distance between nares ≥ 0.35 snout width at same level) (0); close to midline (1); confluent (2) (Laurin, 1991: F2; Müller, 2004: 85; Reisz et al., 2010: 20), ORDERED.

(21) External naris, posterodorsal expansion: absent (0); pinched between nasal and maxilla (1); greatly enlarged, between nasal and lacrimal (2) (Reisz and Dilkes, 2003: 43; Reisz et al., 2010: 21), ORDERED.

(22) Nasal, external narial shelf: absent (0); present (1) (Reisz and Dilkes, 2003: 49; Reisz et al., 2010: 23).

(23) Lacrimal, length: participates in margin of external naris (0); does not reach external naris (1) (Laurin, 1991: B2; Reisz et al., 2010: 24).

(24) Lacrimal, duct: opens on posterior edge of lacrimal (0); opens laterally near posterior edge of lacrimal (1); opens laterally on concave surface of lacrimal (2) (Reisz and Dilkes, 2003: 19; cf. Müller, 2004: 129; Reisz et al., 2010: 25), ORDERED.

(25) Frontal, orbital border: absent or narrow (0); broad and forms most of dorsal edge (1) (Reisz and Dilkes, 2003: 13; Reisz et al., 2010: 26).

(26) Frontal, posterolateral process: absent, fr-par suture forming right angle to parasagittal plane (0); absent or very short, fr-par suture forming obtuse angle to parasagittal plane (1); long, narrow, fr-par suture forming acute angle with parasagittal plane (2) (Reisz and Dilkes, 2003: 4; Müller, 2004: 10; Reisz et al., 2010: 27), ORDERED.

(27) Parietal, extension over interorbital region: absent or marginal (0); present (1) (Reisz and Dilkes, 2003: 16; Reisz et al., 2010: 28).

(28) Parietal, ventrolateral flange: absent (0); present (1) (Laurin, 1991: D1; Reisz et al., 2010: 29). Scored as applicable only in taxa where the parietal borders a temporal fenestra (i.e. in diapsids).

(29) Parietal, sagittal crest: absent (0); present (1) (Laurin, 1991: G2; Müller, 2004: 13 in part; Reisz et al., 2010: 30).

(30) Parietal, size of pineal foramen: large, more than 25% of mid-parietal length (0); small, less than 25% of mid-parietal length (1); absent (2) (Laurin, 1991: G3; Müller, 2004: 12; Reisz et al., 2010: 31), ORDERED.

(31) Parietal, position of pineal foramen in dorsal view: completely enclosed by parietals in the anterior half of the bone (excluding posterolateral processes of the parietals) (0); completely enclosed by parietals close to mid-length or in the posterior half of the bone (excluding posterolateral processes of the parietals) (1); enclosed by both frontals and parietals (2) (Reisz and Dilkes, 2003: 17; Müller, 2004: 12; Reisz et al., 2010: 32). Scored as inapplicable in OTUs that have 31(2), ORDERED.

(32) Prefrontal, suture with nasal: parasagittal, at least in its caudal third (0); anterolateral (1) (Laurin, 1991: E1; Reisz et al., 2010: 33).

(33) Prefrontal and jugal, tuberous ornamentation on their external surface: absent (0); present (1) (Reisz and Dilkes, 2003: 14; Reisz et al., 2010: 34).

(34) Squamosal, anterodorsal process: no or little underlap of posterior process of postorbital (0); extensive underlap of posterior process of postorbital (1) (Reisz and Dilkes, 2003: 8; Reisz et al., 2010: 35). Scored as inapplicable in the absence of a lateral temporal fenestra.

(35) Squamosal, posterodorsal process: absent (0); present (1) (Reisz and Dilkes, 2003: 33; Reisz et al., 2010: 36). Scored as inapplicable in the absence of a lateral temporal fenestra.

(36) Squamosal, ventral process: broad, with proximal length approximately equal to dorsoventral height (0); narrow, with proximal length less than dorsoventral height (1); squamosal confined dorsally (2) (Laurin, 1991: D2, E4; Reisz and Dilkes, 2003: 24; modified from Dilkes, 1998: 34 and Reisz et al., 2010: 37) ORDERED.

(37) Squamosal, occipital shelf: broad, contributes to occipital surface of skull (0); narrow, quadrate exposed in occipital view (1); absent, posterior edge of quadrate exposed in lateral view (2) (Reisz and Dilkes, 2003: 15; Laurin, 1991: B5 in part; Müller, 2004: 132; Reisz et al., 2010: 38), ORDERED.

(38) Supramtemporal fenestra (= dorsal temporal fenestra): absent (0); present, postfrontal does not enter (1); present, postfrontal enters (2) (Laurin, 1991: A1, B3; Dilkes, 1998: 22; Müller, 2004: 89, 90; Reisz et al., 2010: 39), ORDERED.

(39) Infratemporal fenestra (= lateral temporal fenestra): absent (0); present, quadratojugal excluded (1); present, quadratojugal enters or is absent (2) (Laurin, 1991: A2; Reisz and Dilkes, 2003: 3 and 9 and 11 in part; Reisz et al., 2010: 40).

(40) Postorbital region, ventral temporal bar (zygomatic arch): absent (no fenestra) (0); tall, occupying more than 20% of skull height (1); narrow but complete, occupying less than 20% of postorbital skull height (2); incomplete (3); absent (with fenestra) (4) (Laurin, 1991: B4, J1; Reisz and Dilkes, 2003: 18; Müller, 2004: 91; Reisz et al., 2010: 41), ORDERED.

(41) Postorbital region, ventral margin: straight or convex (0); concave, though nowhere dorsal to tooth row (1) (Reisz and Dilkes, 2003: 52; Reisz et al., 2010: 42). Scored as inapplicable in taxa where state 40(4) is present.

(42) Postorbital, dorsal and lateral surfaces: form smooth curve (or dorsal surface absent, postorbital not participating in skull roof) (0); sharply divided (meeting at edge) (1) (Reisz and Dilkes, 2003: 25; Reisz et al., 2010: 43).

(43) Postorbital, lateral boss at orbital margin: absent (0); present (1) (Reisz and Dilkes, 2003: 37; Reisz et al., 2010: 44).

(44) Postorbital, posterior process if temporal fenestrae are absent reaches supratemporal or if at least one fenestra is present extends up to or beyond posterior margin of fenestrae: absent (0); present (1) (Laurin, 1991: I1; Reisz and Dilkes, 2003: 23; Müller, 2004: 131; reworded from Reisz et al., 2010: 45).

(45) Quadratojugal, anterior extent: maxilla-quadratojugal suture (0); extending anterior to ventral portion of squamosal, but not contacting maxilla (1); ≤ anterior extent of ventral portion of squamosal (2); quadratojugal absent (3) (Reisz and Dilkes, 2003: 36; Reisz et al., 2010: 46), ORDERED.

(46) Quadratojugal, anterodorsal process: absent (0); present, covered by squamosal (1); present and superficial (2) (Reisz and Dilkes, 2003: 35; partially in Reisz et al., 2010: 47). This character is not applicable if character 45 has state (3).

(47) Palate, suborbital fenestra: absent (0); present (1) (Laurin, 1991: A3; Reisz et al., 2010: 48).

(48) Pterygoid, teeth on transverse flange: single row on edge (0); additional teeth anterior to single row (or no rows recognizable) (1); absent (2) (Laurin, 1991: E5; Reisz and Dilkes, 2003: 30; Müller, 2004: 163; Reisz et al., 2010: 49).

(49) Parasphenoid, dentition posterior to level of transverse flange: absent (0); along edges (1); on edges and posterior body (2) (Reisz and Dilkes, 2003: 45; Reisz et al., 2010: 50), ORDERED.

(50) Basisphenoid, basipterygoid processes: short, broad, with short articulating facets facing anterolaterally (0); long, wing-like, with long articulating facets facing anteriorly (1); long, with hemispherical articulating facets facing more or less anterolaterally (2) (Reisz and Dilkes, 2003: 20; Müller, 2004: 96; Reisz et al., 2010: 51).

(51) Supratemporal: broad element of skull table (0); slender, in parietal and squamosal trough (1); absent (2) (Reisz and Dilkes, 2003: 22; Müller, 2004: 21; Reisz et al., 2010: 52), ORDERED.

(52) Tabular: large, sheet-like (with ventral expansion) (0); narrow, slender (1); absent (2) (Laurin, 1991: E3; Reisz and Dilkes, 2003: 46; Reisz et al., 2010: 53), ORDERED.

(53) Postparietal, size: sheet-like, both together not much smaller than suproccipital in state when the posttemporal fenestra is small (because of broad, plate-like dorsal process of suproccipital) (0); small, splint-like (1); absent (2) (Laurin, 1991: E2, G5, J2; reworded from Reisz et al., 2010: 54), ORDERED.

(54) Quadrate, shape: straight posteriorly (0); shallowly emarginated (1); with conch (2) (Laurin 1991: E7, J3; Müller, 2004: 29 in part; Reisz et al., 2010: 55), ORDERED.

(55) Quadrate, occipital margin: anterior slope ≥ 80° (0); 80° > anterior slope > 50° (1); anterior slope ≤ 50° (2) (Reisz and Dilkes, 2003: 27; Reisz et al., 2010: 56), ORDERED.

(56) Opisthotic, paroccipital process shape: vertical or nearly vertical sheet, height ≥ 0.5 transverse length (0); elliptical in cross-section, height < 0.5 transverse length (1) (Reisz and Dilkes, 2003: 26; Reisz et al., 2010: 57).

(57) Opisthotic, paroccipital process attachment: ends freely (0); weak contact (1); strong contact (2) (Laurin, 1991: A4, E6; Reisz and Dilkes, 2003: 26; Reisz et al., 2010: 58), ORDERED.

(58) Posttemporal fenestra, size: large compared to suproccipital (narrow dorsal process of suproccipital tapers dorsally) (0); small (because of broad, plate-like dorsal process of suproccipital) (1); more or less foramen (2); absent (3) (Dilkes, 1998: 53, 54; Reisz et al., 2010: 59), ORDERED.

(59) Coronoids, number: two (0); one (1) (Reisz et al., 2010: 60).

(60) Angular, shape of posteroventral edge: ridged or keeled (0); rounded (1) (Reisz and Dilkes, 2003: 38; Reisz et al., 2010: 61).

(61) Angular, size of lateral exposure: wide (0); narrow (1) (Laurin, 1991: J4; Müller, 2004: 167; Reisz et al., 2010: 62).

(62) Retroarticular process, size: absent (0); small (1); large (2) (Laurin, 1991: B6, E10, J5; Reisz et al., 2010: 63), ORDERED.

(63) Retroarticular process, composition: composite (0); formed only by articular (1) (Laurin, 1991: J5; Reisz et al., 2010: 64). This character is scored as inapplicable in taxa with 63(0).

(64) Stapes, shape: robust, with thick shaft (0); slender, rod-like shaft (1) (Laurin, 1991: E8; Reisz et al., 2010: 65).

(65) Stapedial foramen: present (0); absent (1) (Laurin, 1991: E9; Reisz et al., 2010: 66).

(66) Hyoid: short, directed to quadrate region (0); long, directed posteriorly beyond skull (1) (Reisz and Dilkes, 2003: 40; Reisz et al., 2010: 67).

(67) Vertebrae, notochordal canal: present throughout ontogeny (0); absent in adults (1) (Laurin, 1991: F3; Reisz et al., 2010: 68).

(68) Cervical vertebrae, centra length: no longer than posterior dorsals (0); longer than posterior dorsals but fourth and fifth cervical centra less than three times their height (1); longer than posterior dorsals and fourth and fifth cervical centra equal or more than three times their height (2) (Laurin, 1991: H1; modified from Senter, 2004: 28, Müller, 2004: 174, and Reisz et al., 2010: 69). We have added the second character state and modified the first one in order to add the information provided by the character 28 of Senter (2004).

(69) Cervical vertebrae, ventral surface of cervical centra: rounded (0); strongly keeled (1) (Laurin, 1991: H2; Reisz et al., 2010: 70).

(70) Cervical vertebrae, neural arch excavation (lateral to the base of the neural spine): absent (0); shallow (1); deep, represented by a pit (2) (modified from Reisz and Dilkes, 2003: 47; Reisz et al., 2010: 71), ORDERED.

(71) Cervical vertebrae, neural spines: triangular (0); rectangular (1) (Laurin, 1991: C1; Reisz et al., 2010: 72).

(72) Dorsal vertebrae, ratio of height of mid-dorsal neural spines from base of zygapophysis: maximum centrum height: ≤ 1.5 (0); > 1.5 (1) (Reisz and Dilkes, 2003: 31; Reisz et al., 2010: 73).

(73) Dorsal vertebrae, mid-ventral surface of dorsal centra: rounded (0); ridged (with slightly swollen sides) (1); keeled (sharp edge) (2) (Laurin, 1991: H2; Reisz and Dilkes, 2003: 44; Reisz et al., 2010: 74), ORDERED.

(74) Dorsal vertebrae, transverse processes in trunk: short (0); moderately long (1) (Laurin, 1991: F5; Reisz et al., 2010: 75).

(75) Cervico-dorsal vertebrae, mammillary processes on posterior cervical and anterior dorsal neural spines: absent (0); present (1) (Laurin, 1991: H3; Reisz et al., 2010: 76).

(76) Cervical vertebrae, accessory process on anterolateral surface of anterior cervical ribs: absent (0); present (1) (Laurin, 1991: H4; Müller, 2004: 48; Reisz et al., 2010: 77).

(77) Cervical vertebrae, proximal rib heads: some or all holocephalous (0); all dichocephalous (1) (Laurin, 1991: F4; Reisz et al., 2010: 78).

(78) Dorsal vertebrae, trunk ribs: dichocephalous (0); holocephalous (1) (Laurin, 1991: D3; Reisz et al., 2010: 79).

(79) Sacral vertebrae, sacral ribs: two unequal (0); two equal (1); three (2) (Reisz and Dilkes, 2003: 48; Reisz et al., 2010: 80), ORDERED.

(80) Sternum: not mineralized (0); mineralized (bone or calcified cartilage) (1) (Laurin, 1991: A5; Müller, 2004: 81; Reisz et al., 2010: 81). Scored as missing data in taxa withouth sufficiently articulated specimens.

(81) Interclavicle, anterior half shape: +-shaped (anterior process present) (0); T-shaped (anterior process absent) (1) (Laurin, 1991: J6; deBraga and Reisz, 1995: 26; Müller, 2004: 55; Reisz et al., 2010: 82).

(82) Interclavicle, webbed between lateral and posterior processes: yes (head triangular or diamond-shaped) (0); no (rather sharp angles between processes) (1) (Laurin, 1991: J6; Reisz et al., 2010: 83).

(83) Interclavicle, minimal shaft width: ≤ 0.105 tip-to-tip width (0); ≥ 0.137 tip-to-tip width (1) (Laurin, 1991: J6; Reisz et al., 2010: 84).

(84) Cleithrum: present (0); absent (1) (Laurin, 1991: E11; Reisz et al., 2010: 85).

(85) Scapula, anterior margin: straight, at least dorsally (0); convex along entire length (1) (deBraga and Reisz 1995: 27; Reisz et al., 2010: 86).

(86) Scapula, supraglenoid foramen: absent (0); present (1) (Reisz and Dilkes, 2003: 29; Reisz et al., 2010: 87).

(87) Coracoid, triceps process: absent or small (0); large (1) (Laurin, 1991: H5; Reisz et al., 2010: 88).

(88) Humerus, ratio of width of distal head to shaft length: ≥ 0.3 (0); < 0.3 (1) (Laurin, 1991: D4; Reisz et al., 2010: 89).

(89) Humerus, entepicondyle: moderately large (0); strongly developed at maturity (1) (Laurin, 1991: I2; Reisz et al., 2010: 90).

(90) Humerus, entepicondylar foramen: present (0); absent (1) (Laurin, 1991: F6; Reisz et al., 2010: 91).

(91) Humerus, ectepicondylar region: foramen, process bridged (0); supinator process present, groove present (1); process, groove and foramen absent (2) (Laurin, 1991: J7; Müller, 2004: 64, 176; Reisz et al., 2010: 92), ORDERED.

(92) Radius-humerus, length ratio: < 0.68 (0); 0.68 to 0.82 (1); > 0.82 (2) (Laurin, 1991: A6, H7; Reisz et al., 2010: 93), ORDERED.

(93) Radius, shape: straight (0); twisted in lateral view (1) (Laurin, 1991: I3; Reisz et al., 2010: 94).

(94) Ulna, olecranon process: absent or very low (0); prominent but lower than its transverse depth at base (1); strongly developed, being higher than its transverse depth at base (2) (Laurin, 1991: C2; deBraga and Rieppel, 1997; Müller, 2004: 147; modified from Reisz et al., 2010: 95), ORDERED. The original character of Reisz et al. (2009) was split into two different characters. Character 95 describes the degree of development of the olecranon process and character 162 describes if the process is ossified separately or not. Moreover, an additional state was added to character 95 to include the degree of development of the prominent olecranon processes.

(95) Carpus, medial centrale carpi: present (0); absent (1) (Laurin, 1991: F7; Reisz et al., 2010: 96).

(96) Carpus, lateral centrale carpi: large (0); small or absent (1) (Laurin, 1991: E12; Reisz et al., 2010: 97).

(97) Pelvic girdle: solid (0); fenestrate (= open acetabulum) (1) (Laurin, 1991: J8; reworded from Reisz et al., 2010: 98).

(98) Pubis, lateral and distal pubic tubercles: small or absent (0); large (1) (Laurin, 1991: H6; Reisz et al., 2010: 99).

(99) Pelvic girdle, acetabulum: elongate (0); circular (1) (Laurin, 1991: B7; Reisz et al., 2010: 100).

(100) Hindlimb-trunk, length ratio: hindlimb much shorter than trunk (0); hindlimb almost as long as trunk or longer (1) (Reisz et al., 2010: 101).

(101) Femur, distal articular surface: uneven, fibular condyle projecting distinctly beyond tibial condyle (0); both condyles prominent and approximately at same level (1); both condyles do not project beyond shaft (distal articular surface concave or almost flat) (2) (Laurin, 1991: B8; modified from Rieppel et al., 1999, and Müller, 2004: 72; Reisz et al., 2010: 102). We have modified state (1) and added a third sate (2) in order to include the information provided by the character 72 of Müller (2004).

(102) Femur, maximum length/distal width ratio: < 4 (0); ≥ 4 (1) (Reisz and Dilkes, 2003: 21; Reisz et al., 2010: 103).

(103) Femur-humerus, length ratio: > 1.2 (0); 1 to 1.2 (1); < 1 (2) (Laurin, 1991: C3; Reisz et al., 2010: 104), ORDERED.

(104) Femoral-humerus, shaft diameters: femur = 150% humerus (0); more or less equal (up to 120%) (1) (deBraga & Reisz 1995: 38; Reisz et al., 2010: 105).

(105) Lower leg-foot length, ratio: articulated tibia + tibiale/astragalus longer than articulated 4th metatarsal + digit (0); shorter (1) (deBraga and Reisz, 1995: 33, 42; Reisz et al., 2010: 106).

(106) Proximal tarsals, astragalus-calcaneum articulation: flat (0); concave-convex (1); foramen on calcaneum, articulation expanded (2); sutured or fused (3) (Laurin, 1991: F8; Müller, 2004: 171; Reisz et al., 2010: 107).

(107) Proximal tarsals, lepidosauriform ankle joint: absent (0); present (1) (Laurin, 1991: J9; Müller, 2004: 171; Reisz et al., 2010: 108).

(108) Proximal tarsals, lateral tuber on calcaneum: absent (0); present (1) (Laurin, 1991: F9; Müller, 2004: 75; Reisz et al., 2010: 109).

(109) Metatarsus, metapodials overlapping proximally: absent (0); present (1) (deBraga and Reisz, 1995: 43; Reisz et al., 2010: 110).

(110) Metatarsus, fourth metatarsal: short (0); long (at least 40% of digit IV) (1) (Laurin, 1991: F10; Reisz et al., 2010: 111).

(111) Metatarsus, fifth distal tarsal: present (0); absent (1) (Laurin, 1991: E13; Reisz et al., 2010: 112).

(112) Metatarsus, fifth metatarsal: straight (0); hooked (1) (Laurin, 1991: E14; Müller, 2004: 80; Reisz et al., 2010: 113).

New characters added to the original character list of Reisz et al. (2010):

(113) Skull, relative length of snout: <50% of total skull length (0); equal to or >50% of total skull length (1) (Dilkes 1998: 2). This character is independent from character 6 because there are some operational taxonomic units in which the length of the snout is less than 50% of the total skull length, but the antorbital part of snout is still longer than the total skull length (e.g. *Mycterosaurus*, *Mesenosaurus*, *Aerosaurus*, *Varanops*, *Erythrosuchus*, some *Proterosuchus* specimens). It is also independent from character 22 because in some taxa that the nasal is longer than the frontal (e.g. *Coelurosauravus*, *Erythrosuchus* and some specimens of *Proterosuchus*: BSPG 1934-VIII-514, BP/1/4016), the snout represents less than 50% of the total skull length. Although characters 6 and 22 are potentially clustered to each other, we decided to keep the original character sampling of Reisz et al. (2010) in the present analysis.

(114) Premaxilla, contact with prefrontal: absent (0); present (1) (Dilkes 1998: 7).

(115) Premaxilla, premaxillary body size: small, the premaxillary body forms less than half of snout in front of the posterior border of the external nares (0); large, the premaxillary body forms half or more than half of snout in front of the posterior border of the external nares (1) (Rieppel et al., 1999; modified from Müller, 2004: 1).

(116) External nares, shape: rounded (0); elongate (1) (Dilkes 1998: 12; Müller, 2004: 86).

(117) Snout, diastema between maxillary and premaxillary teeth: absent (0); present (1) (Nicholls, 1999; Müller, 2004: 116). This character is considered inapplicable in taxa without premaxillary teeth (e.g. *Trilophosaurus*, *Hyperodapedon*).

(118) Antorbital fenestra: absent (0); present (1) (Dilkes 1998: 5).

(119) Maxilla, antorbital fossa exposed in lateral view: absent (0); present (1) (modified from Benton 2004; Dilkes and Sues 2009: 3). This character is not applicable for taxa that lack an antorbital fenestra.

(120) Maxilla, anterior maxillary foramen: absent (0); present (1) (Dilkes, 1998: 17; Müller, 2004: 88; Modesto and Sues, 2004). See discussion of the distribution of the condition of this character is Modesto and Sues (2004: 346-347).

(121) Maxilla, ventral margin: straight, concave or sigmoid (0); convex (1) (modified from Dilkes 1998: 16).

(122) Maxilla, posterior extension: at level or posterior to posterior orbital border (0); anterior to posterior orbital border (1) (deBraga and Rieppel, 1997; Müller, 2004: 127).

(123) Maxilla, orbital exposure: absent (0); present (1) (deBraga and Rieppel, 1997; Müller, 2004: 128).

(124) Maxilla, tooth plate: absent (0); present (1) (Dilkes 1998: 60).

(125) Maxilla, number of tooth rows: single row (0); multiple rows (1) (Dilkes 1998: 61).

(126) Maxilla, location of teeth: only on occlusal surface (0); on occlusal and lingual surfaces (1) (Dilkes 1998: 63).

(127) Nasal, shape of anterior margin at midline: strongly convex with anterior process (0); transverse with little convexity (1) (Dilkes 1998: 13).

(128) Frontal, suture with nasal: transverse (0); oblique, forming an angle of at least 30° with long axis of the skull and frontal(s) entering between both nasals (1); oblique, and nasal entering considerably between frontal(s) in a non-interdigitate suture (2) (deBraga and Rieppel, 1997; modified from Müller, 2004: 154).

(129) Frontal, narrowly approaches or enters the anteromedial margin of the supratemporal fossa: absent, in which the suture between the frontals and parietals is usually transverse (0); present (1) (Rieppel et al., 1999; modified from Müller 2004: 178). This character is considered inapplicable in taxa that lack of a supratemporal fenestra.

(130) Frontal, shape of dorsal surface next to sutures with postfrontal and parietal: flat to slightly concave (0); longitudinal depression with deep pits is present (1) (Dilkes 1998: 20).

(131) Postfrontal, shape of dorsal surface: flat or slightly concave towards raised orbital rim (0); depression present with deep pits (1) (Dilkes 1998: 21).

(132) Quadratojugal, anterior process: present (0); absent (1) (Rieppel et al., 1999; Müller, 2004: 20).

(133) Parietal, median contact between both parietals: suture present (0); fused with loss of suture (1) (Dilkes 1998: 25).

(134) Skull roof, distinct posterior emargination in late ontogeny: absent (0); present (1) (Müller, 2004: 177).

(135) Vomer, contact with maxilla: absent (0); present (1) (Dilkes 1998: 38; Müller, 2004: 92).

(136) Palatine, palatal teeth: present (0); absent (1) (Dilkes, 1998: 67; Müller, 2004: 99).

(137) Pterygoids, contact between each other: join cranially (0); remain separate (1) (Dilkes 1998: 126).

(138) Pterygoid, palatal process: extends anterior to the anterior limit of the palatine (0); forms oblique suture with palatine but process ends before reaching anterior limit of palatine (1); forms transverse suture with palatine (2) (deBraga and Rieppel, 1997; Müller, 2004: 139).

(139) Pterygoid, teeth on palatine ramus: present in two fields (0), present in one field (1), present in three fields (2); absent (3) (Dilkes 1998: 68; Müller, 2004: 100).

(140) Pterygoid, orientation of the transverse flange: directed predominately laterally (0); directed anterolaterally (1) (deBraga and Rieppel, 1997; Müller, 2004: 140).

(141) Pterygoid, transverse flange lateral margin: posterolateral margin with an acute corner (0); posterolateral margin merges smoothly into anterolateral margin forming a smoothly convex lateral outline (1) (deBraga and Rieppel, 1997; Müller, 2004: 164).

(142) Ectopterygoid: simple overlap of ectopterygoid and pterygoid (0); complex overlap between ectopterygoid and pterygoid (1); ectopterygoid absent (2) (modified from Dilkes 1998: 142).

(143) Ectopterygoid, shape along suture with pterygoid: transversely broad (0); posteroventrally elongate and does not reach lateral corner of transverse flange (1); posteroventrally elongate and reaches corner of transverse flange (2) (Dilkes 1998: 42; Müller, 2004: 95), ORDERED. This character is not applicable to taxa that lack of an ectopterygoid (e.g. *Captorhinus*; Fox and Bowman 1966) (see character 131).

(144) Ectopterygoid, contact with maxilla: absent (0); present (1) (Dilkes, 1998: 40; Müller, 2004: 94).

(145) Ectopterigoid, posterior expansion in contact with jugal: absent (0); present (1) (modified from Dilkes 1998: 39). This character is not applicable to taxa that lack of an ectopterygoid (e.g. *Captorhinus*; Fox and Bowman 1966) (see character 131).

(146) Opisthotic, paroccipital processes orientation: extend laterally forming 90° with parasagittal plane (0); deflected posterolaterally at an angle of about 20° from the transverse width of the skull (1) (deBraga and Rieppel, 1997; modified from Müller, 2004: 158).

(147) Opisthotic, paroccipital processes shape: slender (0); robust, with anteroposterior dimension at least one third greater than dorsoventral dimension (1) (deBraga and Rieppel, 1997; modified from Müller, 2004: 159).

(148) Opisthotic, club-shaped ventral ramus: absent (0); present (1) (Dilkes 1998: 46).

(149) Laterosphenoid: absent (0); present (1) (Dilkes 1998: 50).

(150) Parasphenoid, position of foramina for entrance of internal carotid arteries leading to the pituitary fossa: lateral wall of braincase (0); ventral surface of parasphenoid (1) (Parrish 1993; Dilkes 1998: 45).

(151) Basioccipital-basisphenoid, fused to each other: absent (0); present (1) (modified from deBraga and Rieppel, 1997; Müller, 2004: 137).

(152) Parasphenoid, cultriform process: reaching forward to the level of the posterior limit of the internal nares (0); short, not reaching the level of the internal nares (1) (deBraga and Rieppel, 1997; modified from Müller, 2004: 141).

(153) Prootic, lateral surface: continuous and slightly convex (0); crista prootica present (1) (Dilkes 1998: 47).

(154) Prootic, anterior inferior process: absent (0); present (1) (Dilkes 1998: 48).

(155) Prootic, contact with parietal: absent (0); present (1) (deBraga and Rieppel, 1997; Müller, 2004: 160).

(156) Abducens foramina: in dorsum sella (0); between prootic and dorsum sella (1) (Dilkes 1998: 49).

(157) Occipital condyle, position: even with craniomandibular joint (0); anterior to craniomandibular joint (1); posterior to craniomandibular joint (2) (Dilkes 1998: 51).

(158) Lower jaw, distinct dorsal process behind the alveolar margin: absent, with a slightly convex dorsal margin behind the alveolar portion (0); present, formed by a dorsally well-developed surangular (1); present, formed by a dorsally well-developed coronoid and sometimes the posterodorsal ramus of the dentary (2); present, formed only by a dorsally hypertrophied coronoid bone (3) (Rieppel et al., 1999; modified from Müller, 2004: 36).

(159) Lower jaw, Meckelian fossa orientation: dorsomedially (0); mostly dorsally due to greatly expanded prearticular resulting in a ventral border of the fossa situated dorsal to the half height of the lower jaw at that level (1) (deBraga and Rieppel, 1997; Müller, 2004: 165).

(160) Dentary, number of tooth rows: one (0); two (1); more than two (2) (Dilkes 1998: 64), ORDERED.

(161) Jaw occlusion: single-sided overlap (0); flat occlusion (1); blade and groove (2) (Dilkes 1998: 65).

(162) Surangular, anterior extension: beyond coronoid eminence (0); posterior to reaching the anterior border of the coronoid eminence (1) (deBraga and Rieppel, 1997; Müller, 2004: 143).

(163) Surangular, lateral shelf: absent (0); present (1) (deBraga and Rieppel, 1997; Müller, 2004: 166).

(164) Surangular, anterior surangular foramen: absent (0); present (1) (Modesto and Sues, 2004: 145).

(165) Surangular, posterior surangular foramen: absent (0); present (1) (Modesto and Sues, 2004: 146).

(166) External mandibular fenestra: absent (0); present (1) (Dilkes 1998: 76).

(167) Retroarticular process: not upturned (0); upturned (1) (Dilkes 1998: 75; Müller, 2004: 101). This character is scored as inapplicable in taxa with 63(0).

(168) Cervical vertebrae, postaxial cervical intercentra: present (0); absent (1) (Dilkes 1998: 79; Müller, 2004: 43).

(169) Cervical vertebrae, distinct longitudinal lamina extending along the lateral surface of the centrum at mid-height in anterior cervical vertebrae: absent (0); present (1) (new character).

(170) Cervical vertebrae, longitudinal lamina connecting the prezygapophysis and postzygapophysis in the third cervical neural arch: absent (0); present (1) (new character).

(171) Cervical vertebrae, dimensions of postaxial anterior cervical neural spines: tall, with height and length approximately equal (0); long and low, with height lower than length (1) (modified from Dilkes 1998: 82).

(172) Cervical vertebrae, postaxial cervical neural spines with an anterior overhang: absent (0); present (1) (modified from Senter 2004: 30).

(173) Cervical ribs, slender and tapering at low angle to vertebrae: absent (0); present (1) (Dilkes 1998: 77; Müller, 2004: 102).

(174) Cervico-dorsal vertebrae, parallelogram centra in lateral view, in which the anterior articular surface is situated higher than the posterior one: absent (0); present (1) (Bonaparte, 1975; Novas, 1996).

(175) Dorsal vertebrae, intercentra: present (0); absent (1) (Rieppel et al., 1999; Müller, 2004: 42).

(176) Dorsal vertebrae, length of the anterior dorsal centra: subequal to the height of the centrum (0); more than two times the height of the centrum (1) (modified from Sereno, 1999)

(177) Dorsal vertebrae, subcentral foramen in the lateral surface of the centra: absent (0); present (1) (new character).

(178) Dorsal vertebrae, ratio between transverse width of diapophysis and length of the centrum in anterior dorsal vertebrae: <0.65 (0); >0.75 (1) (new character).

(179) Dorsal vertebrae, anterior and middle dorsal neural spines: sub-rectangular, with the anterior margin vertical, anterodorsally or slightly posterodorsally inclined (0); sub-triangular, with the anterior margin strongly posterodorsally oriented (1) (new character).

(180) Dorsal vertebrae, anterior centrodiapophyseal lamina or paradiapophyseal lamina: absent (0); present (1) (modified from Galton, 1990).

(181) Dorsal vertebrae, posterior centrodiapophyseal lamina: absent (0); present (1) (modified from Galton, 1990).

(182) Dorsal vertebrae, prezygodiapophyseal lamina in posterior cervicals and anterior-middle dorsals: absent (0); present (1) (modified from Bonaparte, 1986).

(183) Dorsal vertebrae, postzygodiapophyseal lamina in anterior dorsals: absent (0); present (1) (modified from Coria & Salgado, 2000).

(184) Dorsal vertebrae, dorsally opened pit lateral to the base of the neural spine: absent (0); present (1) (modified from Dilkes 1998: 84; Müller, 2004: 103 in part)

(185) Dorsal vertebrae, zygapophyses close to each other medially, respectively, in anterior-middle dorsals: absent, zygapophyses laterally divergent beyond the lateral margin of the centrum (0); present, zygapophyses mainly oriented in the parasagittal axis (1) (new character).

(186) Dorsal vertebrae, zygosphene-zygantrum articulation: absent (0); present (1) (Rieppel et al., 1999; Müller, 2004: 44).

(187) Sacral ribs, second sacral rib: not bifurcate (0); bifurcate with posterior process pointed bluntly (1); bifurcate with posterior process truncated sharply (2) (Dilkes 1998: 87; Müller, 2004: 105).

(188) Sacral and/or anterior caudal vertebrae, transverse processes and ribs: sutured to the vertebra (0); fused to the vertebra (1) (Rieppel et al., 1999; Müller, 2004: 50).

(189) Caudal vertebrae, anterior caudal neural spine height: moderately tall with height/length between >1.0 and <2.0 (0); low with height/length <1.0 (1); tall with height/length >2.0 and <3.0 (2); very tall with height/length >3.0 (3) (Dilkes 1998: 88; Müller, 2004: 106).

(190) Caudal vertebrae, transverse processes: absent beyond fifth caudal (0); present beyond fifth caudal (1) (deBraga and Rieppel, 1997; Müller, 2004: 169).

(191) Caudal vertebrae, ratio of lengths of transverse processes and centra in anterior caudal vertebrae: equal or <1.0 (0); >1.0 (1) (Dilkes 1998: 89).

(192) Chevrons, distal width of haemal spine: equivalent to proximal width (0); tapering (1); wider than proximal width (2) (Dilkes 1998: 91; Müller, 2004: 108).

(193) Gastralia: present (0); absent (1) (Dilkes, 1998: 92; Müller, 2004: 109).

(194) Clavicles, position: anteroventrally to the interclavicle (0); dorsally to interclavicle (1) (Rieppel et al., 1999; Müller, 2004: 53).

(195) Interclavicle, anterior margin with a median notch: absent (0); present (1) (modified from Dilkes 1998: 97; Müller, 2004: 111).

(196) Interclavicle, posterior stem: little change in width along entire length (0); expansion present (1) (Dilkes 1998: 98; Müller, 2004: 112).

(197) Humerus, torsion between proximal and distal ends: around 45° or more from one another (0); 20° or less from one another (1) (Müller, 2004: 145).

(198) Humerus, capitellum (radial condyle) and trochlea (ulnar condyle): strongly developed as distinct ball-shaped structures (0); poorly developed but distinct from the ectepicondyle and entepicondyle (1); absent (2) (modified from Rieppel et al. 1999 and Müller 2004: 63), ORDERED.

(199) Humerus, trochlea (ulnar condyle) situated approximately at mid-width of the distal end of the bone: present (0); absent, it is considerably laterally displaced (1) (new character). This character is inapplicable for taxa that lack or have incipient radial and ulnar condyles.

(200) Ulna, olecranon process as a separate ossification: absent (0); present (1) (modifed from Laurin, 1991: C2). See character 95.

(201) Ulna, olecranon process in lateral view: tapering toward its distal end (0); subrectangular or slightly expanded towards its distal end (1) (new character). This character is not applicable to taxa without an olecranon process (e.g. *Macrocnemus*).

(202) Radius, length: shorter than ulna (0); longer than ulna or approximately of the same length as the ulna (1) (modified from Rieppel et al., 1999, and Müller, 2004: 66). The olecranon process of the ulna should not be taken into account if present.

(203) Manus: greater than humeral length (0); subequal to or lower than humeral length (1) (Senter 2004: 51).

(204) Metacarpus, fourth metacarpal: longer than metacarpal III (0); equal or shorter than metacarpal III (1) (deBraga and Rieppel, 1997; Müller, 2004: 148).

(205) Ilium, anteroposterior development of the iliac blade: well developed (0); reduced (1) (Rieppel et al., 1999; Müller, 2004: 67).

(206) Ilium, iliac blade: posterior process only (0); large posterior process and smaller anterior process (1); equally developed anterior and posterior processes (2); large anterior projection (3) (Dilkes 1998: 102; Müller, 2004: 113 in part).

(207) Pubis, pectineal process: absent (0); present (1) (new character).

(208) Pubis and ischium, thyroid fenestra: absent (0); present (1) (Rieppel et al., 1999; Müller, 2004: 68).

(209) Pubis, anterior apron: absent (0); present (1) (Dilkes 1998: 104).

(210) Femur, shaft: diameter constant or widening distally (0); diameter distally narrowed (1) (Senter 2004: 61).

(211) Tibia, articulation with astragalus: loose fitting (0); tightly fitting with well-developed articulation (1) (deBraga and Rieppel, 1997; Müller, 2004: 170).

(212) Proximal tarsus, foramen for the passage of the perforating artery between the astragalus and calcaneum (= perforating foramen): present (0); absent (1) (Rieppel et al., 1999; Müller, 2004: 74).

(213) Astragalus, articulation with distal tarsal 4: poorly defined (0); well defined (1); absent (2) (deBraga and Rieppel, 1997; Müller, 2004: 150).

(214) Distal tarsus, number of pedal centralia: both medial and lateral centralia present (0); only lateral pedal centrale and does not contact tibia (1); only lateral pedal centrale and contacts the tibia (2); pedal centralia absent as sperate ossifications (3) (deBraga and Rieppel, 1997; modified from Dilkes 1998: 117; Müller, 2004: 151), ORDERED.

(215) Distal tarsus, distal tarsal 1: present (0); absent (1) (Rieppel et al., 1999; Müller, 2004: 76).

(216) Metatarsus, ratio of lengths of metatarsals I and IV: equal or >0.42 (0); <0.42 and equal to or >0.32 (1); equal to or <0.32 (2) (Dilkes 1998: 123; Müller, 2004: 173; modified from Modesto and Sues 2004: 123). The ratio of the supposed rhynchosaurs *Mesosuchus* (0.32; SAM-PK-7416), *Noteosuchus* (0.30; AM 3591) and *Hyperodapedon gordoni* (Benton, 1983: fig. 36) depart from those of other diapsids. Accordingly, the boundary between states (1) and (2) was moved to 0.32 (i.e. the upper rhynchosaur limit), ORDERED.

(217) Metatarsus, metatarsal V lateral plantar tubercle in late ontogeny: absent (0); present (1) (new character).

(218) Metatarsus, metatarsal V medial plantar tubercle in late ontogeny: absent (0); present (1) (deBraga and Rieppel, 1997; modified from Müller, 2004: 172).

(219) Pedal digits, ratio of lengths of digits III and IV: equal or <0.8 (0); >0.8 and <0.9 (1); equal to or >0.9 (2) (Dilkes 1998: 124), ORDERED.

APENDIX SII

Character states modified from Reisz et al. (2010) original data matrix.

Characters 6 and 22 of Reiz et al. (2010) were merged together because the all taxa with nasals longer than frontals have a preorbital portion of the skull longer than the postorbital one in the current taxonomic sample. The addition of a state (i.e. preorbital region longer the postorbital and nasal longer than frontal) resulted in the following changes. Additionaly, the modified multistate character 6 was treated as additive here.

Changed from (1) to (2): *Archaeothyris*, *Ophiacodon*, *Coelurosauravus*, *Youngina*, *Prolacerta*, *Proterosuchus*, *Erythrosuchus*, *Tanystropheus*, *Euparkeria*, *Protorosaurus* and *Macrocnemus*. In particular, in the case of *Youngina*, the scoring for the character 22 of Reisz et al. (2010) was a missing entry for this taxon. However, a first hand observation of GHG K106 allowed recognizing that the nasals are longer than the frontals in this specimen.

Changed from (1) to (?): *Acerosodontosaurus*. The absence of most of the preorbital region in the holotype of *Acerosontosaurus* (Bickelmann et al., 2009) does not allow to recognize the condition in this taxon.

Character 12: the second state of the character of Reisz et al. (2010) was splited into two states that take into account the development of the postnarial process with respect to its contribution of the border of the external naris.

Chnaged from (0) to (?): *Elliotsmithia*.

Changed from (0) to (1): *Mesenosaurus*, *Huehuecuetzpalli*, *Dalinghosaurus*.

Changed from (0) to (2): *Eothyris* and *Varanodon*.

Changed from (1) to (2): *Trilophosaurus*, *Prolacerta*.

Character 31:

Changed from (?) to (0): *Araeoscelis*.

Changed from (0) to (1): *Tseajaia*, *Eothyris*, *Archaeothyris*, *Ophiacodon*, *Mycterosaurus*, *Mesenosaurus*, *Elliotsmithia*, *Aerosaurus*, *Varanops*, *Varanodon*, *Archaeovenator*, *Paleothyris*, *Youngina* and *Prolacerta*.

Changed from (2) to (?): *Araeoscelis* and *Chalarodon madagascariensis*.

Changed from (2) to (0): *Captorhinus* and *Dalinghosaurus*.

Changed from (2) to (1): *Planocephalosaurus*.

Character 46: the character was modified in order to include the third state (anterodorsal process of quadratojugal covered by squamosal; Reisz and Dilkes, 2003; Reisz et al., 2009).

Changed from (0) to (1): *Casea*, *Eothyris*, *Archaeothyris*, *Ophiacodon*, *Mesenosaurus*, *Elliotsmithia*, *Apsisaurus* and *Myceterosaurus* and *Archaeovenator* (these terminals were scored as question marks by Reisz et al. [2009], but they were scored as [0] in Reisz et al. [2010]).

Changed from (1) to (2): *Aerosaurus*, *Varanops* and *Varanodon*.

Character 94:

Changed from (1) to (2): *Araeoscelis*, *Petrolacosaurus*, *Paleothyris*, *Captorhinus* and *Ophiacodon*.

Character 101:

Change from (1) to (0): *Tseajaia* and *Varanops*.

Change from (1) to (1/2): *Chalarodon madagascariensis*.

Change from (1) to (2): *Coelurosauravus*.

*Acerosodontosaurus*

Character 9 and 11: the premaxilla is not preserved in the holotype and only known specimen of *Acerosodontosaurus* (MNHN 1908-32-57) and, as a result, the scorings of these characters were changed from (0) to (?).

Character 13: only the base of the ascending process of the maxilla is preserved (MNHN 1908-32-57) and, as a result, the degree of dorsal development of the process cannot be determined. Accordingly, the scoring was of this character was changed from (1) to (1/3).

Character 14: Bickelmann et al. (2009) inferred a contact between the maxilla and prefrontal based on the shape of the lacrimal. Accordingly, the scoring of this character was changed from (?) to (1).

Character 16: the anterior end of the maxilla is not preserved and, as a result, is not possible to determine if the most anterior teeth were caniniform or not (MNHN 1908-32-57). Accordingly, the scoring of this charater was changed from (0) to (?).

Character 24: the lacrial duct opens laterally on the lacrimal, close to the posterior margin of the bone (MNHN 1908-32-57). As a result, the scoring of this character was changed from (0) to (1).

Character 32: the morphology of the suture between the prefrontal and nasal cannot be confidently determined and, particularly, because the skull is exposed in medial view (MNHN 1908-32-57). Accordingly, the scoring of this character was changed from (0) to (?).

Character 38: the anterior end of the anterior process of the preserved postorbital and the parietals are missing. As a result, it is not possible to determine if the postfrontal participated in the supratermporal fenestra or not (i.e. if it was a contact between the postorbital and parietal) (MNHN 1908-32-57). Accordingly, the scoring of this character was chaged from (2) to (1/2).

Character 40: Bickelmann et al. (2009) stated that the jugal lacks a contact with the quadratojugal. Accordingly, the state of this character was changed from (2) to (3).

Character 41: the ventral margin of the jugal, between its main body and the base of the posterior process, is concave (MNHN 1908-32-57). Accordingly, the scoring of this character was changed from (0) to (1).

Character 42 and 43: only the mould of the medial surface of the postorbital is preserved and it cannot be determined the morphology of the lateral surface of the bone (MNHN 1908-32-57). Accordingly, the states of these characters were changed from (0) to (?).

Character 45: it is not clear if the quadratojugal was absent or not (Bickelmann et al. 2009), but it is clear that this bone did not contact the maxilla (MNHN 1908-32-57). Accordingly, the state of this character was changed from (1) to (1/2/3).

Character 46: the quadratojugal is not preserved (if it was present) (Bickelmann et al. 2009). Accordingly, the state of this character was changed from (0) to (?).

Character 55: the quadrate is detached from the rest of the skull and it is not possible to determine the angle of the anterior slope (MNHN 1908-32-57). Accordingy, the state of this character was changed from (0) to (?).

Character 63: only the medial surface of the retroarticular process is preserved as a mould and, as a result, it is not possible to determine if the surangular participated laterally in the retroarticular process or not (MNHN 1908-32-57). Accordingy, the state of this character was changed from (0) to (?).

Character 69: the condition of this character cannot be confidently determined in the holotype of *Acerosodontosaurus* (MNHN 1908-32-57). As a result, it is preferred here to score the character as (?) rather than (0).

Character 70: the cervicals lack a depression or pit lateral to the base of the neural arch (MNHN 1908-32-57). Accordingly, the state of this character was changed from (1) to (0).

Character 72: the height of the neural spine varies through the dorsal series, with neural spineheight:centrum height values that range above and below 1.5. Accordingly, the state of this character was changed from (0) to (0&1).

Character 73: the condition of this character cannot be confidently determined in the holotype of *Acerosodontosaurus* (MNHN 1908-32-57). As a result, it is preferred here to score the character as (?) rather than (1).

Character 76: none anterior cervical ribs are preserved in MNHN 1908-32-57 and, as a result, the scoring of this character was changed from (0) to (?).

Character 78: Bickelmann et al. (2009) described that all the dorsal ribs are dichocephalous. Accordingly, the state of this character was changed from (1) to (0).

*Araeoscelis*

Character 68: the addition of a third character state resulted in the rescoring of *Aeroscelis* from (1) to (2) because the ratio between the length and height in the fourth and fifth cervical centra is 3.97–4.12 based on Vaughn (1955: fig. 5a).

Character 89: in *Araeoscelis* the entepicondyle is strongly developed in comparison with the shaft width at mid-length (Vaugh 1955: fig. 8). Accordingly, the scoring of this character was changed from (0) to (1).

*Capthorinus*

Character 81: the interclavicle lacks an anterior process (Fox and Bowman, 1966: fig. 27). Accordingly, the scoring of this character was changed from (0) to (1).

*Chalarodon*

Character 36: the scoring of this character was changed from (2) to (1) based on the discussion of Modesto and Sues (2004: 347) and YPM 12866.

Character 98: the condition of this character cannot be assessed here. Accordingly, the scoring of this character was changed from (0) to (?).

Character 104: the condition of this character cannot be assessed here. Accordingly, the scoring of this character was changed from (1) to (?).

*Coelurosauravus*

Character 1: the tooth implantation is pleurodont (Evans and Haubold, 1987: 297). Accordingly, the scoring of this character was changed from (0&3) to (2).

Character 5: the nasal has a large vertical contribution to the snout (SMNS 59349 cast of SSWG 113/7; Evans and Haubold, 1987: figs. 2, 6). Accordingly, the scoring of this character was changed from (?) to (1).

Character 6: the postorbital length of the skull is greater than the preorbital length (SMNS 59349; Schaumberg et al. 2007: fig. 4). Accordingly, the scoring of this character was changed from (2) to (0).

Character 7: the preserved skulls of *Coelurosauravus* are strongly compressed in order to determine the condition of this character (Evans and Haubold, 1987; Schaumberg et al., 2007). Accordingly, the scoring of this character was changed from (1) to (?).

Character 9: the premaxilla of *Coelurosauravus* lacks an anterior process (SMNS 59349). Accordingly, the scoring of this character was changed from (?) to (0).

Character 19: it is unknown the presence or absence of a septomaxilla in *Coelurosauravus*. Accordingly, the scoring of this character was changed from (0) to (?).

Character 20: the external nares are situated in a marginal position (SMNS 59349). Accordingly, the scoring of this character was changed from (?) to (0).

Character 25: the frontal broadly participates in the border of the orbit (Evans and Haubold 1987: fig. 3). Accordingly, the scoring of this character was changed from (?) to (1).

Character 26: the posterolateral process of the frontal is long and forms an acute angle with the parasagittal plane (Evans and Haubold 1987: fig. 3). Accordingly, the scoring of this character was changed from (1) to (2).

Character 28: the posterior extensions of the parietals in order to form the posttemporal frill can be considered homologous to the posterolateral (= posteroventral) processes present in other neodiapsids because they delimite the supratemporal fenestrae and articulate laterally with the supratemporal/squamosal complex of *Coelurosauravus*. Accordingly, the scoring of this character was changed from (0) to (1).

Character 30: the pineal foramen represents at least 35% of the length of the mid-parietal length (Schaumberg et al. 2007: fig. 4). Accordingly, the scoring of this character was changed from (?) to (0).

Character 31: the pineal foramen is completely enclosed by the parietals and situated close to the mid-length of the bone (Evans and Haubold 1987: fig. 3; Schaumberg et al., 2007: fig. 4). Accordingly, the scoring of this character was changed from (?) to (1).

Character 32: the suture between the nasal and prefrontal is anterolaterally oriented (SMNS 59349; Evans and Haubold, 1987: fig. 3). Accordingly, the scoring of this character was changed from (?) to (1).

Character 34: Evans and Haubold (1987: fig. 6) reconstructed an extensive underlap of the anterior process of the squamosal on the posterior process of the postorbital. The condition of the character is considered ambiguous here because of the poorly preserved temporal region of the available specimens. Accordingly, the scoring of this character was changed from (0) to (?).

Character 43: there is no lateral boss on the postorbital (SMNS 59349). Accordingly, the scoring of this character was changed from (1) to (0).

Character 47: *Coelurosauravus* possesses a suborbital fenestra (Evans and Haubold 1987: 300). Accordingly, the scoring of this character was changed from (?) to (1).

Character 52: the tabulars are absent in *Coelurosauravus* (Evans and Haubold 1987: 297). Accordingly, the scoring of this character was changed from (?) to (2).

Character 53: the postparitals are absent in *Coelurosauravus* (Evans and Haubold 1987: 297). Accordingly, the scoring of this character was changed from (?) to (2).

Character 54: the quadrate of *Coelurosauravus* is not emarginated (Evans and Haubold 1987: 301) and neither possesses a conch. Accordingly, the scoring of this character was changed from (?) to (0).

Character 63: the condition of this character cannot be determined and, as a result, the socring was changed from (0) to (?).

Character 67: the vertebrae of *Coelurosauravus* are notochordal (Carroll, 1978; Evans, 1982). Accordingly, the scoring of this character was changed from (?) to (0).

Character 69 and 73: the condition of this character cannot be determined and, as a result, the socring was changed from (0) to (?).

Character 70: the cervical vertebrae lack an excavation lateral to the base of the neural spine (SMNS 59349; Evans and Haubold 1987: fig. 14). Accordingly, the scoring of this character was changed from (?) to (0).

Character 71: the cervical neural spines of of *Coelurosauravus elivensis* are subrectangular, whereas those of *Coelurosauravus jaekeli* are subtriangular (Evans and Haubold 1987: 288, figs. 9a, 13a). Accordingly, the scoring of this character was changed from (0) to (0&1).

Character 77: the condition of this character cannot be confidently determined (Evans and Haubold 1987: 284). Accordingly, the scoring of this character was changed from (0) to (?).

Character 85: the anterior margin of the distal end of the scapula is gently concave (Evans and Haubold 1987: fig. 14). Accordingly, the scoring of this character was changed from (?) to (0).

Character 86: there is no evidence of a supraglenoid foramen (Evans and Haubold 1987: fig. 14). Accordingly, the scoring of this character was changed from (?) to (0).

Character 94: the ulna of *Coelurosauravus* possesses a completely ossified and high olecranon process (Evans and Haubold 1987: fig. 14). As a result of the modification of the original character-states the scoring was changed from (1) to (2).

Character 96: *Coelurosauravus* has a large lateral centrale (Evans and Haubold 1987: fig. 15). Accordingly, the scoring of this character was changed from (?) to (0).

Character 98: the condition of this character cannot be determined. Accordingly, the scoring of this character was changed from (0) to (?).

Character 99: the condition of this character cannot be determined. Accordingly, the scoring of this character was changed from (1) to (?).

Character 101: the distal condyles of the femur are prominent (Evans and Haubold 1987: figs. 11, 16), but the condition seems to be polymorphic. Accordingly, the scoring of this character was changed from (2) to (0/1).

Character 110: the condition of this character cannot be assessed. Accordingly, the scoring of this character was changed from (0) to (?).

*Dalinghosaurus*

Character 8: Evans and Wang (2005: 728) described the presence of seven premaxillary teeth. Accordingly, the scoring of this character was changed from (1) to (0).

Character 23: the lacrimal is lost or fused (Evans and Wang 2005: 727) and, as a result, the scoring is innaplicable. The scoring was changed from (?) to (-).

Character 34: Evans and Wang (2005: 730) described that the length of the squamosal probably implied a contact with the jugal. If it is the case, the squamosal should have extensively underlaped the posterior process of the postorbital. Accordingly, the scoring of this character was changed from (0) to (1).

Character 36: the scoring of this character was changed from (2) to (0/1) based on the discussion of Modesto and Sues (2004: 347) and Evans and Wang (2005: 730, fig. 3).

Character 50: the basipterygoid processes are very long and anterolaterally oriented (Evans and Wang, 2005: fig. 2b). Accordingly, the scoring of this character was changed from (0) to (2).

Character 67: the scoring of this character was changed from (?) to (1) based on the scoring of Evans and Wang (2005) of the character 167 (presence of notochordal centra) in the character list of Lee (1998).

Character 69: the cervical vertebrae have a ventral keel on the centrum (Evans and Wang 2005: fig. 6a). Accordingly, the scoring of this character was changed from (?) to (1).

Character 88: the ratio between the width of distal end and the total length of the humerus is 0.32 (Ji and Ji 2004: 900). Accordingly, the scoring of this character was changed from (1) to (0).

Character 89: the entepicondyle is strongly developed with respect to the width of the shaft (Ji and Ji 2004: fig. 3). Accordingly, the scoring of this character was changed from (0) to (1).

Character 92: the radius:humerus length ratio is 0.61 based on Ji and Ji (2004: fig. 3b). Accordingly, the scoring of this character was changed from (1) to (0).

Character 94: Ji and Ji (2004: fig. 3b) clearly showed an olecranon process developed as a separate ossification from the rest of the ulna. This process is well proximally developed, but its transverse width cannot be determiend and, as a result, cannot be assessed if state (1) or (2) is present. Accordingly, the scoring of the character was changed from (?) to (1/2).

Character 96: Ji and Ji (2004: 901) described a rounded and very small centrale. This bone articulates with the intermidiate (Ji and Ji 2004: fig. 3b) and, as a result, it is interpreted here as a lateral centrale. The bone is proportionally smaller than in basal diapsids (e.g. *Acerosodontosaurus*: MNHN 1908-32-57). Accordingly, the scoring of this character was changed from (?) to (1).

Character 97: there is no enough information to determine the condition of this character and, as a result, its scoring was changed from (1) to (?).

Character 98: the pubis possesses a large lateral tubercle similar to that of some “pelycosaurs” and araeosclidans (Ji and Ji, 2004: fig. 4). Accordingly, the scoring of this character was changed from (?) to (1).

Character 105: the metatarsal IV + fourth digit is considerably longer than the tibia + astragalocalcaneum (Ji and Ji, 2004: fig. 1; Evans and Wang, 2005: figs. 4, 7, 8, 9a). Accordingly, the scoring of this character was changed from (0) to (1).

Character 110: the length of the metatarsal IV represents 48% (based on Evans and Wang, 2005: fig. 7) to 51.2% (based on Ji and Ji, 2004: table 5) of the total length of the fourth pedal digit. Accordingly, the scoring of this character was changed from (0) to (1).

*Huehuecuetzpalli*

Character 2: the tooth crowns are not distally curved (Reynoso 1998: figs. 4, 5). Accordingly, the scoring of this character was changed from (0) to (1).

Character 36: the scoring of this character was changed from (2) to (0) based on the discussion of Modesto and Sues (2004: 347) and Reynoso (1998: fig. 4).

Character 57: the desarticulated nature of the available specimens does not allow determining the morphology of the attachment between the parapoccipital process and the temporal region of the skull. As a result, the scoring of this character was changed from (2) to (?).

Character 69: the cervical vertebrae are not exposed in ventral view in the available specimens (Reynoso 1998). Accordingly, the scoring of this character was changed from (0) to (?).

Character 70: in the cervical vertebrae of both individuals there is no excavations lateral to the neural spines (Reynoso 1998: figs. 1, 5a). Accordingly, the scoring of this character was changed from (?) to (0).

Character 71: the cervical neural spines of the juvenile specimen are rectangular in lateral view (Reynoso 1998: fig. 2). Accordingly, the scoring of this character was changed from (?) to (1).

Character 73: the condition of this character cannot be determined and, as a result, the scoring of the character was changed from (0) to (?).

Character 76: the anterior cervical ribs lack an anterior process (Reynoso 1998: fig. 1). Accordingly, the scoring of this character was changed from (?) to (0).

Character 82: the angle between the lateral and posterior processes is sharp and, as a result, the scoring of this character was changed from (?) to (1).

Character 89: in *Huehuecuetzpalli* the entepicondyle is strongly developed in comparison with the shaft width at mid-length (Reynoso 1998: fig. 1). Accordingly, the scoring of this character was changed from (0) to (1).

Character 98: the pubis possesses dorsolaterally projected, large process that closely resembles the lateral tubercle of some “pelycosaurs” and araeoscelidans (Reynoso 1998: fig. 8b). Accordingly, the scoring of this character was changed from (?) to (1).

Character 104: the femoral shaft diameter represents 136% of the humeral shaft diameter (Reynoso 1998: fig. 1). Accordingly, the scoring of this character was changed from (1) to (0).

*Hyperodapedon gordoni*

Character 36: the scoring of this character was changed from (2) to (1) based on the discussion of Modesto and Sues (2004: 347) and Benton (1983: fig. 5).

Character 57: the paroccipital processes of *Hyperodapedon gordoni* have a weak contact with the parietals (Benton 1983: figs. 6, 7) and, as a result, the scoring of this character was changed from (2) to (1).

*Paleothyris*

Character 89: in *Paleothyris* the entepicondyle is strongly developed in comparison with the shaft width at mid-length (Carroll 1969: fig. 7e). Accordingly, the scoring of this character was changed from (0) to (1).

*Planocephalosaurus*

Character 19: it is unknown the presence or absence of a septomaxilla in *Planocephalosaurus* (Fraser, 1982). Accordingly, the scoring of this character was changed from (0) to (?).

Character 26: the posterolateral process of the frontal is long and the suture with the parietal forms an acute angle with the parasagittal plane (Fraser, 1982: fig. 1c). Accordingly, the scoring of this character was changed from (1) to (2).

Character 30: the length of the pineal foramen represents 25.5% of the length of the parietal along its mid-width based on Fraser (1982: fig. 1c). Accordingly, the scoring of this character was changed from (1) to (0).

Character 36: the scoring of this character was changed from (2) to (1) based on the discussion of Modesto and Sues (2004: 347) and Fraser (1982: fig. 1).

Character 61: the postdentary bones of *Planocephalosaurus* are fused with each other (Fraser, 1982) and, as a result, it is not possible to determine the degree of exposition of the angular in lateral view. Accordingly, the scoring of this character was changed from (1) to (?).

Character 63: the postdentary bones of *Planocephalosaurus* are fused with each other (Fraser, 1982) and, as a result, it is not possible to determine if retroarticular process of the lower jaw was composed only by the articular or not. Accordingly, the scoring of this character was changed from (1) to (?).

Character 83: the posterior ramus of the interclavicle is not known (Fraser and Walkden 1984). Accordingly, the scoring of this character was changed from (0) to (?).

Character 88: the ratio between the width of the distal end of the humerus and the length of the bone is 0.37 (Fraser and Walkden 1984: fig. 14b). Although the proximal margin of the bone is missing the value is considerably higher than 0.3 and, as a result, the scoring of the character was changed from (1) to (0).

Character 89: in *Planocephalosaurus* the entepicondyle is strongly developed in comparison with the shaft width at mid-length (Fraser and Walkden 1984: fig. 14). Accordingly, the scoring of this character was changed from (0) to (1).

Character 97: the acetabulum is completely closed (Fraser and Walkden 1984: fig. 16). Accordingly, the scoring of this character was changed from (1) to (0).

Character 100: the trunk vertebral count in unknown and, as a result, the ratio between the hindlimb and trunk lengths cannot be determined. Accordingly, the scoring of this character was changed from (1) to (?).

Character 104: the mean of the diameters of the humerus and femora are not known. Accordingly, the scoring of this character was changed from (1) to (?).

*Prolacerta broomi*

Character 18: the septomaxilla of *Prolacerta broomi* possesses a curved lateral lip (Modesto and Sues, 2004) and, as a result, is not a completely flat sheet of bone. Accordingly, the scording of this character was changed from (2) to (1).

Character 36: the scoring of this character was changed from (2) to (1) based on the discussion of Modesto and Sues (2004: 347).

Character 53: the postparietals are absent in *Prolacerta broomi* (Modesto and Sues, 2004). Accordingly, the scording of this character was changed from (1/2) to (2).

Character 57: in *Prolacerta broomi* the paroccipital processes possess a weak contact with the posterior temporal region of the skull (BP/1/471). Accordingly, the scoring of this character was changed from (2) to (1).

Character 68: the addition of a third character state resulted in the rescoring of *Prolacerta broomi* from (1) to (2) because the ratio between the length and height of the centrum is 3.26 in the fourth cervical and 3.68 in the fifth cervical vertebrae of BP/1/2675.

Character 69: the cervical vertebrae have a ventral keel in the centrum (BP/1/2675). Accordingly, the scoring of this character was changed from (0) to (1).

Character 73: the anterior dorsal vertebrae of *Prolacerta* have a ridged ventral surface of the centrum, but the middle and posterior dorsal verterbrae have a transversely convex ventral surface (BP/1/2675). Accordingly, the scoring of this character was changed from (1) to (0&1).

Character 75: the posterior cervical and anterior dorsal vertebrae of BP/1/2675 have mammillary processes on the lateral surface of the neural spines. Accordingly, the scoring of this character was changed from (0) to (1).

Character 77: the condition for this character is not known through the entire cervical series of *Prolacerta broomi* (e.g. BP/1/2675) and, as a result, the scoring was changed from (1) to (?).

Character 81: the interclavicle of *Prolacerta* lacks an anterior process (BP/1/2675). Accordingly, the scoring of this character was changed from (0) to (1).

Character 85: the anterior margin of the scapular blade is severely damaged in the scapulae of BP/1/2675 and, as a result, the condition of the character cannot be determined. Accordingly, the scoring of this character was changed from (1) to (?).

Character 86: the supraglenoid foramen is absent in BP/1/2675. Accordingly, the scoring of this character was changed from (?) to (0).

Character 92: the radius-humerus length ratio is 0.88 based on Gow (1975: 111; BP/1/2675). Accordingly, the scoring of this character was changed from (?) to (2).

Character 99: the scoring of this character was changed from (0) to (1) because in BP/1/2676 the height and length of the acetabulum are subequal.

*Trilophosaurus*

Reisz *et al*. (2010) did not mention which species of *Trilophosaurus* they considered for their scorings. However, we have employed only *Trilophosaurus buettneri* for the scorings of the present data matrix.

Character 2: this scoring was changed from (1) to (2) because *Trilophosaurus buettneri* lacks of a distal curvature in the margin teeth (Spielmann et al. 2008: figs. 18, 20, 21).

Character 5: the snout of *Trilophosaurus buettneri* is narrow and tall, and the nasal has a strong vertical contribution to the snout (Spielmann et al. 2008: figs. 20-22). Accordingly, this scoring was changed from (0) to (1).

Character 12: the dorsolateral process of the premaxilla is well extended beyond the posteiror margin of the external naris (Spielmann et al. 2008: fig. 20). Accordingly, the scoring of this character was changed from (?) to (1).

Character 13: the dorsal process of the maxilla is present and extends to the dorsal margin level of the external naris (Spielmann et al. 2008: fig. 20). As a result, the scoring of this character was changed from (?) to (1).

Character 24: the new interpretations of the skull of *Trilophosaurus buettneri* strongly differs from those of Gregory (1945) regarding the shape and position of the lacrimal and the foramen showed by this author should not be positioned within the lacrimal based on Parks (1969) and Spielmann et al. (2008). However, the latter author did not give information about the passage of the lacrimal duct in *Trilophosaurus buettneri*. Accordingly, the scoring of this character was changed from (0) to (?).

Character 25: the orbital border of the frontal is broad in TMM 31025-207 (Spielmann et al. 2008: fig. 20e, f). Accordingly, the scoring of this character was changed from (0) to (1).

Character 27: the parietals do not extend over the interorbital region in TMM 31025-140 (Spielmann et al. 2008: fig. 19). As a result, the scoring of this character was changed from (1) to (0).

Character 39: the lateral temporal fenestra is absent in *Trilophosaurus buettneri* (Gregory 1945, Spielmann et al. 2008). Accordingly, the scoring of this character was changed from (0&2) to (0).

Character 40: the lateral temporal fenestra is absent in *Trilophosaurus buettneri* (Gregory 1945, Spielmann et al. 2008). Accordingly, the scoring of this character was changed from (0&4) to (0).

Character 41: the ventral margin of the postorbital region of the skull is severely damaged in TMM 31025-140 and the condition of the character cannot be confidently assessed (Spielmann et al. 2008: fig. 19). Accordingly, the scoring of this character has been changed from (0) to (?).

Character 45: the quadratojugal of TMM 31025-140 is broken and the anterior extent of this bone cannot be assessed with the currently available specimens (Spielman et al. 2008: fig. 19). As a result, the scoring of this character has been changed from (2) to (?).

Character 46: we consider that this character cannot be assessed with the currently available specimens of *Trilophosaurus buettneri* and the scoring was changed from (1) to (?).

Character 51: the supratemporal is absent in *Trilophosaurus buettneri* (Gregory 1945: 278). Accordingly, the scoring of this character has been changed from (1&2) to (2).

Character 53: the postparietal cannot be discerned in *Trilophosaurus buettneri* (Gregory 1945: 278) and, as a result, the scoring was changed from (1&2) to (2).

Character 57: the paroccipital processes of *Trilophosaurus buettneri* have a weak contact with the parietals (Spiealmann et al. 2008: fig. 18) and, as a result, the scoring of this character was changed from (2) to (1).

Character 61: the exposition of the angular in lateral view is wide in TMM 31025-5 (Spielmann et al. 2008: fig. 28). Accordingly, the scoring was changed from (?) to (0).

Character 68: some cervical vertebrae are longer then the posterior dorsals and others shorter (Spielman et al. 2008: appendix 10). Accordingly, the scoring of this character was changed from (0) to (0&1).

Character 69: the ventral surface of the cervical centra is strongly keeled in *Trilophosaurus buettneri* (Spielmann et al. 2008). Accordingly, the scoring of this character was changed from (0) to (1).

Character 77: the axial cervical rib possesses a single articular head (Spielmann et al. 2008: figs. 30, 31). Accordingly, the scoring of this character was changed from (1) to (0).

Character 79: *Trilophosaurus buettneri* has two unequal sacral vertebrae (Spielmann et al. 2008) and, as a result, the scoring of this character was changed from (?) to (0).

Character 83: this ratio is lower than 0.09 in TMM 31025-68B. Accordingly, the scoring of this character was changed from (1) to (0).

Character 85: the anterior margin of the scapular blade is not completely preserved in the available specimens of *Trilophosaurus buettneri* (Speilmann et al. 2008). Accordingly, the scoring of this character was changed from (0) to (?).

Character 88: based on the measurements provided by Spielmann et al. (2008: appendix 10) the ratio between the width of the distal end of the humerus and the length of the bone ranges between 0.27-0.36. This ratio is usually lower in smaller specimens. Accordingly, the scoring of this character was changed from (0) to (0&1).

Character 89: the entepicondyle is well laterally projected considerably larger than the ectepicondyle (Spielmann et al. 2008: fig. 67). Accordingly, the scoring of this character was changed from (0) to (1).

Character 92: in TMM-31025-140 the ratio between the radial length (14.8 cm: Spielmann et al. 2008: fig. 72) and the humeral length (17.0 cm: Spielmann et al. 2008: appendix 10) is of 0.87. Accordingly, the scoring of this character was changed from (1) to (2).

Character 94: in *Trilophosaurus buettneri* the olecranon process is prominent but it is not ossified separately (Spielmann et al. 2008: figs. 69, 70). As a result, the scoring of this character was changed from (0) to (1).

Character 103: in the articulated skeleton of TMM 31025-140 the ratio between the femoral and humeral length is 1.21 (Spielmann et al. 2008: appendix 10). However, this value is very close to the boundary between states (0) and (1). Accordingly, the scoring of this character was changed from (1) to (0/1).

Character 109: the calcaneal tuber is absent in TMM 31025-140 (Spielmann et al. 2008: fig. 91) and, as a result, the scoring of this character was changed from (1) to (0).

*Tseajaia*

Character 18: the septomaxilla is present and is not pillar-like (Moss 1972), as a result the scoring of the character was changed from (0) to (1).

Character 35: a posterodorsal process in the squamosal is absent. Accordingly, the scoring of this character was changed from (1) to (0).

*Youngina*

Character 1: Gow (1975) described a subthecodont tooth implantation for *Youngina* and the cronws are not fused to the tooth-bearing bone (e.g. BP/1/3859, GHG K 106), contrasting with the condition present in some specimens of *Prolacerta broomi* (Modesto and Sues, 2004) and *Proterosuchus fergusi* (e.g. RC 59, 96). Accordingly, the scoring of this character was changed from (1) to (0).

Character 24: the foramen for the opening of the nasolacrimal duct could not be located in any of the studies specimens (BP/1/2459, 3859, GHG K 106, GHG RS 160, TM 1490, 4095). Accordingly, the scoring of this character was changed from (0) to (?).

Character 32: the suture between the prefrontal and nasal is anterolaterally oriented along its entire extension (BP/1/3859, GHG K 106). Accordingly, the scoring of this character was changed from (0) to (1).

Character 41: the ventral margin of the ventral temporal bar (i.e. formed by the posterior process of the jugal and the anterior process of the quadratojugal) is concave (BP/1/3859). Accordingly, the scoring of this character was changed from (0) to (1).

Character 66: the hyoids were not recognized in the studied specimens and neither mentioned by Gow (1975). Accordingly, the scoring of this character was changed from (0) to (?).

Character 80: the presence of mineralized sternal plates in *Youngina* was confirmed by Smith and Evans (1996). Accordingly, the scoring of this character was changed from (?) to (1).

Character 82: the anterior half of the interclavicle is T-shaped, with sharp angles between lateral and posterior processes (Gow, 1975: 9c; Smith and Evans, 1996: 6c). Accordingly, the scoring of this character was changed from (0) to (1).

Character 84: the presence of cleithrum is uncertain in *Youngina capensis* (Smith and Evans, 1996: 296). Accordingly, the scoring of this character was changed from (0) to (?).

Character 87: the triceps process seems to be absent in the coracoid of *Youngina* (Smith and Evans, 1996: fig. 6c). Accordingly, the scoring of this character was changed from (?) to (0).

Character 91: a supinator process and an ectopicondylar groove are present is *Youngina* (BP/1/3859). Accordingly, the scoring of this character was changed from (?) to (1).

Character 94: *Youngina* lacks an olecranon process in the ulna (Gow 1975: fig. 9a). Accordingly, the scoring of this character was changed from (?) to (0).

Character 97: the pelvic girdle is solid in *Youngina* (BP/1/3859). Accordingly the scoring of this character was changed from (1) to (0).

Character 100: the hindlimb is considerably longer than the trunk (Smith and Evans, 1996: fig. 4). Accordingly the scoring of this character was changed from (?) to (1).

Character 103: the femur-humerus length ratio ranges between 1.12–1.27 in *Youngina* based on Smith and Evans (1996: table 1). Accordingly the scoring of this character was changed from (1) to (0&1).

Character 105: the metatarsal IV + digit 4 are considerably longer than the tibia (Smith and Evans, 1996: table 1). Accordingly the scoring of this character was changed from (?) to (1).

Character 107: *Youngina* does not have a “lepidosauriform ankle joint” (BP/1/3859). Accordingly the scoring of this character was changed from (?) to (0).

Character 109: the metatarsals overlap proximally with each other (BP/1/3859). Accordingly the scoring of this character was changed from (?) to (1).

Character 110: the length fourth metatarsal represents 44% of the length of its respective digit (BP/1/3859) and the ratio is even larger in Smith and Evans (1996: fig. 8a). Accordingly the scoring of this character was changed from (?) to (1).

APPENDIX SIII

TNT file of the data matrix corresponding to the data set analyzed in this paper. Inapplicable characters are marked with a dash.

xread

219 40

Tseajaia 01000101?[0 1]1000021100000?010-001?0-0-10000000100100000110010000-00?00001000001000?0100100101101?00?000?10000000?0001110-011000000-00000011110000?00000?00000?0110000000-000????00010000000001??1??0?110?0?010000000010??????

Casea [0 1]10000011[0 1]001000-0000100010-0010001000110001010100001010120000-00000001001001020000000001010010001000021100000000?0100-0001000??-????0?000101????000???1????0000010000-1?000???0??0????????????0????00?0????0310?0000[0 1]00???

Eothyris [0 1]1000001100200021?000100010-00100010001100010101?00000100[2 3]00?0-?????????????????????????????????????????????????001100-000100001-00000????0??????0?0??1?????00?0000000-????????????????????????????????????????????????????

Archaeothyris 0100121?0??000110??00000120-001?00?0??11110111?0?010[0 1]0??03?0?0-0??0?00111?0??000111??0?0101?????01??001????0?0???????0-?0?0000?1-00?00??????????????????????0??00?????-?00000000000?000000?0?1?0????00??????0010?0?????????

Ophiacodon [0 1]1?112100000001100000000110-0010000000111101110000100010030000-000000011110010000[0 1]100000101102000100001000001000101100-011000001-00000010?000??1000000?1000?1200011001-0?00000000101000010?031000000000011??001000000001??0

Mycterosaurus [0 1]0111110001031010?001011121-0010101010120111210?11110010120?0??0010111111?00?010????110?001?000?010?1100?000?00000?100-0010000?1-00000?0?????????000?1?1????10???10000?????????????????????????????????0????????????1??????

Mesenosaurus [0 1]01111100011310110001011121-001010102012011121011111000?03000100010111101000?010?????10?001?00000???110??000?0?0001100-001?00000-0000000000110010?01??0?????02000010000????????????????????????????????0???????????????????

Elliotsmithia 0011?11?????????????????121-001?1110101201111100??1100201[1 2 3]?0010??1???????????????????????????????????????????????????????1?000??-00000????[0 1 2]0?????????????????0??00???0?0?0000??????????????????????????????????????????????

Aerosaurus 000111110010200120102012121-001011?12022011012002111?0201?01010??1011111100010200000000000110000000111??12001100001000-000000002-00000????0??????110000?000010000000000???000?0?0?000000??[1 2]1?1??00??0??0?10000?0?0000?0????

Varanops [0 1]00111110010201120102012121-0010111120220110120?211110201?010100?10?111120001020001000100012000000010101120011000010?1010000000?-00000????[0 1 2]??????1????0?000?10?0000?0010????0?000000000110?1????0???0000??00000000000?0????

Varanodon [0 1]00110110012201120112012121-001011?12022011012?0?11110201[2 3]01010??101111121001020???0001000110000?00??10?????11??0010010000000000-00000??????????????????????10??0???001?0000?0?00?00000?????????0??????0?1?0???????????????

Archaeovenator [0 1]100111100100010-0102010121-001000101012010121000111000??[1 2 3]?000-??100001010001010000?01????????0?0??1?1??10001000000000-101?00001-00000?0?001?00?0?????01?????000000000-0??000?00??0???????01?100??00????????001000000100???

Captorhinus 310000011100000101000000010-00010-0-00000000200100120001011101000000000?0000000010100100102002000001001110000000001000-001011100-00000[0 1]00?0002---??0010???0000021?00000???000?0???100000??01???1001000100?0?000000000[0 1]00???

Paleothyris [0 1]1000100000000120100000?110-01100-0-0000000020010011001?011000-00000000020001000?0?00101101002000000011010000000001000-000000000-000000001000????000??01????0000010000-000100?00001000000??0?1??001000100??1001000?00000??0

Petrolacosaurus 01000100000000120100000012100110000011210000101110110001011000-00?0112111011000000101111001202000101001000001000000100-1010000010000000000011000000?010100??0000000000-000100?0000010001??000101101000?0?100011000000100??0

Araeoscelis 010001?0000000020??00000121001000-0-11000000201100110001111000-00?0212001011000100101111100202000101010000001000001100-001000002100000???[0 1]0110010000000?????0000010100-000100000000000010001010110?000100??0001000000100???

Apsisaurus [0 1 2]100????????????????????????????1??02?120???210[0 1]?1???0[0 1]0???00?????011110100??01?000?0101001100??00??1000?????????????????1?000?????0?????[0 1]?0???????0??0??????0???01000?10??1?10?00?101000??00?0??0??02-0-1????1000?????????

Coelurosauravus 210010?000001100-??0101?121100110?0?2224-000201???02200????101????00?0[0 1]0?000?0?????000?100010200???1[0 1]11110001?00001110-001100001000100??????????0????????????0?000???0?1001[0 1]00110?100000??0??0?10????010?111?????001010000?

Acerosodontosaurus 0100????????[1 3]11?-?????11121??????001?[1 2]231??1[1 2 3]?????????????1??1????00?01[0 1]?00??0?????0????101?1000001?1??1?????????????0-?010000??????????????????0????????????0?00????01????0?0101?01000000??????0????100-1?000??00?????????

Youngina 0100020100001110-100001?12110111000122221001101000110101101101000?000010000001[0 1]1010?000010111000001111[0 1]110001100000100-001000000100000000120100?01010001010100000?0000000000000000010000000?111210000100-1?0101000000101000

Trilophosaurus_buettneri 12001?1200021?00-?00001?120112-101022100?00???120022210110?102011?1[0 1]11111101010?0001?00[0 1]11120111001111[0 1]1110111110011-0-100100001100?01011?3????10100011?11??1210010000[0 1]000011100010001100001[0 2]100?10110100?10011000000101??0

Prolacerta 1001020001021000-101001112110[1 2]110001212300002010001221011111020???121010[0 1]111?10?1001?000011200110011110101011111001100-1010000011001010010000?00?11101001101100000011010011111000001010110110112?01002-0-110010010000[1 2]?????

Planocephalosaurus 3100010100001100-??1001?1201001101012223000020120222220????1?2????000000?000010?11?10010100?????001?110??3101??1000000-101100000100-10000120101101??010?????02?0010000000000000?001000000121?11??01?02-?????101100?1????11?

Chalarodon_madagascariensis 2200000100001100-1010010100101?100012[1 2]24-0003-1202122201201112111110?1011000010001011001000101011?1?[1 2]10?13100011000000-00100000200?-10011111101100100??011??23000111000????????????????????????????????1???????????????????

Huehuecuetzpalli 2200110000011100-??1001[0 1]1001012000002124-0003-1???122201??111??1?1?0?010?00001[0 1]1?1?1?000100101??11?1110013101011000100-001000001000-10??????????????0???????03?0000000?0000?0?00?010000001?111101??01??10001011100111310110

Dalinghosaurus 21000?0000011100-??100-11001010101?[0 1]2124-0003-12?212220?[1 2]?1112????101000?0-001100111?0001?000[1 2]?1?1?1110013101111000100-011000002000-1000??1????10???0??????003?000110000?0??0?10?0-0000??001?11010100????01101110011?310??0

Proterosuchus_fergusi 10110210020210[0 1]0-101001?1[0 1 2][0 1]10[1 2]110001212[2 3]00012010001201012[1 3]11020111111[1 2]10011101001111000?011?001?001?10???1011?11[0 1]011110110000001100[0 1]010010200?2011111111111110000011111000001100010100[0 1]11021[0 2]1120010?2-001??010010000200?0?

Erythrosuchus_africanus 4011021001021000-2-10011000102-00001212200101012002211012111020?1?1012112101?00?????0000011100??001120?1?10111110011011001000000100001?10?301??01101111?111110000010111000?0?10001011101100011000???12-011??01001001031?10?

Aenigmastropheus_parringtoni ??????????????????????????????????????????????????????????????????0??0??1?0?????????????111??2???????????????????????????????????????????????????????????????????????????????1?01?11110010???????????0000??????????????????

Tanystropheus_longobardicus 0[0 1 2]000200000[1 2]1100-??100111[0 1]111[0 1][0 2]10001222300003-1200?2210[0 1]00110201?112101021010[0 1]?0???100?1011[0 1]00110001110010001111101100-001000001000-110000[1 3]000??010?0?1110??00?0000000011011111100011110000101120??012-0-111010111001?1010[1 2]

Euparkeria_capensis 4011020100021000-2-1001?111102-00001212200002012002211011111120?1?11101001010000??010000012200??00111100?10111110010011011000001100001?0110001101101111?111110?00011111000001110000111011?01210100?012-001?1010010010310???

Mesosuchus_browni 41000001010211?0-2-200111101110100012123000020120002210111?1120??01110100101010010010000011?0011001110?1?1011?11011100-10100100111111010002011111111011011?111?21000001100001?00000000010?21[2 3]1120011?2-0-??00[0 1]1010000202?0?

Howesia_browni 12000?0?????[1 2]1-????2001?110112-0000121?30000??12000221?1?11?120???1?101?01????0?11???0??01??????001?1????101??1??1??????11?11111111?10?0?[0 1]?0011111??011?????11?21??00011?0????0?0???0001??21211200??1???????01101000020?10?

Protorosaurus_speneri [0 2][1 2]0002?100011010-2-1??1111011[1 2]??0001?1?30000??1???22?10110?1020???12?[0 1]11?1110[0 1]00000?011011120200?0?1111?[0 1]00011?1100100-101000000100?[0 1]1????2??????100????????00?0000?00110011111000011111100021020?101[0 1]110210??0011000100000

Archosaurus_rossicus 1??????00202??????????????????????????????????????????????????????????????????????????????????????????????????????11???????????????????????????????????????????????????????????????????????????????????????????????????????

Paliguana ?200010?????????????????1?010[0 1][0 2]10?01212??000??120??2220?????????????????????????????????????????????????????????00???0-?01??????100?10??0??????????00???????0??00?0??00????????????????????????????????????????????????????

Macrocnemus_bassanii 000002?000021100-???????121112-1000?21?30000???????2???1???10????112?0102001012010010001011200??[0 1]0011100?0001?11101?00-101000001000?01????0?????????0??????????00?0?0000111111110001?1000?21?1120010?2-0-01[0 1]01011100020[0 1]11?

Noteosuchus_colletti ??????????????????????????????????????????????????????????????????1????1010???0?????????????????001110???1011?11??????????????????????????????????????????????????????????????01??00000???[1 2]1211?0??????????000?010000[1 2]0210?

Gephyrosaurus 2200010000001110-??110101101011101002223000020120222220???1112????00000?1000010001?1?1?0100??[1 2]??001??????310??11000100-10110000110011000012000110???0101???012000101000000?0000?1010000001?1?112000?0???????10110011?3??11?

Eorasaurus_olsoni ??????????????????????????????????????????????????????????????????1[0 1]11???1?????????????????????????????????????????????????????????????????????????????????????????????010???1???10111001??????????????????????????????????

;

ccode + 1 5 7 9 11 15.17 19 20 23 25 29 30 35.37 39 44 48 50.54 56 57 61 69 72 78 90 91 93 102 142 159 197 213 215 218 *;

proc/;

APPENDIX SIV

List of unambiguous synapomorphies in the sauropsid branch recovered in the MPT.

Sauropsida: 17(1→0), 45(1→2), 52(0→1), 56(0→1), 59(0→1), 94(1→2).

*Paleothyris* + Diapsida: 8(1→0), 25(0→1), 30(0→1), 73(0→1), 88(0→1), 102(0→1), 171(0→1).

Diapsida: 26(1→2), 27(0→1), 37(0→1), 38(0→1), 47(0→1), 109(0→1), 128(0→1), 141(0→1).

Araeoscelidia: 70(0→2), 75(0→1), 76(0→1), 85(0→1), 87(0→1), 92(1→2), 98(0→1), 105(1→0), 140(0→1), 184(0→1), 193(0→1).

Neodiapsida: 13(0→1), 14(0→1), 16(1/2→0), 23(0→1), 32(0→1), 37(1→2), 40(0/1→3), 60(0→1), 62(0→1), 86(1→0), 203(0→1).

“Younginiformes” + Sauria: 54(0→1), 88(1→0), 94(2→1), 171(1→0), 192(1→2), 198(0→1).

“Younginiformes”: 15(0→1), 41(0→1), 44(0→1), 93(0→1), 94(1→0), 180(0→1), 199(1→0).

Sauria: 20(0→1), 26(2→1), 27(1→0), 62(1→2), 64(0→1), 65(0→1), 84(0→1), 96(0→1), 111(0→1), 112(0→1), 143(0→1), 153(0→1), 217(0→1).

Lepidosauromorpha: 1(0→2), 50(0→2), 54(1→2), 61(0→1), 91(1→0), 106(0→3), 107(0→1), 133(0→1), 208(0→1), 211(0→1), 212(0→1), 214(1/2→3), 218(0→1).

Rhynchocephalia: 34(0→1), 123(0→1), 162(0→1), 205(0→1).

Squamata: 129(1→0).

*Dalinghosaurus* + *Chalarodon*: 128(1→2), 163(0→1), 164(0→1).

Archosauromorpha: 29(0→1), 74(0→1), 76(0→1), 90(0→1), 92(1→2), 134(0→1), 138(1→0), 141(1→0), 151(0→1), 173(0→1), 174(0→1), 182(0→1), 189(1→2), 197(0→1).

Protorosauria: 113(0→1), 171(0→1), 175(0→1), 180(0→1), 181(0→1), 207(1→0), 210(0→1).

Protorosauridae: 94(1→2), 185(0→1).

Tanystropheidae: 27(0→1), 73(1→2), 88(0→1), 89(1→0), 94(1→0), 129(1→0), 169(0→1), 176(0→1), 207(0→1).

*Trilophosaurus* + Archosauriformes: 1(0→1), 106(0→1), 108(0→1), 157(0→1).

Rhynchosauria + Archosauriformes: 10(0→1), 58(0→1), 73(1→0), 81(0→1), 89(1→0), 94(1→0), 145(0→1), 147(0→1), 148(0→1), 184(0→1).

Rhynchosauria: 20(1→2), 51(1→0), 61(0→1), 114(0→1), 125(0→1), 130(0→1), 131(0→1), 133(0→1), 134(1→0), 158(0→1), 160(0→2), 161(0→1), 168(0→1), 182(1→0), 216(0/1→2).

*Prolacerta* + Archosauriformes: 2(1→0), 4(0→1), 14(1→0), 29(1→0), 144(1→0), 164(0→1), 165(0→1), 180(0→1), 185(0→1), 207(1→0).

Archosauriformes: 3(0→1), 53(2→1), 118(0→1), 149(0→1), 155(0→1), 163(0→1), 166(0→1).

Proterosuchidae: 10(1→2).

*Eorasaurus + Erythrosuchus + Euparkeria*: 1(1→4), 32(1→0), 51(1→2), 78(1→0), 119(0→1), 120(1→0), 147(1→0), 181(0→1), 191(1→0), 212(0→1), 214(2→3), 215(0→1).

APPENDIX SV

Supplementary references.

Benton MJ (2004) Origin and relationships of Dinosauria. In: Weishampel DB, Dobson P, Osmólska H, editors. The Dinosauria. 2nd edition. Berkeley: University of California Press. pp. 7–24.

Bonaparte JF (1975) Nuevos materiales de *Lagosuchus talampayensis* Romer (Thecodontia - Pseudosuchia) y su significado en el origen de los Saurischia. Chañarense inferior, Triásico Medio de Argentina. Acta Geologica Lilloana 13: 5–90.

Bonaparte JF (1986) Les Dinosaures (Carnosaures, Allosauridés, Sauropodes, Cetiosauridés) du Jurassique Moyen de Cerro Cóndor (Chubut, Argentine). Ann Paléontol 72: 247–289.

Carroll RL (1969) A Middle Pennsylva-nian captorhinomorph, and the interrelationships of primitive reptiles. J Paleontol 43: 151–70.

Carroll RL (1978) A Gliding Reptile from the Upper Permian of Madagascar. Palaeontol Afr 21: 143–159.

Coria RA, Salgado L (2000) A basal Abelisauria Novas 1992 (Theropoda– Ceratosauria) from the Cretaceous of Patagonia, Argentina. GAIA 15: 89–102.

deBraga M, Reisz RR (1995) A new diapsid reptile from the uppermost Carboniferous (Stephanian) of Kansas. Palaeontology 38: 199–212.

Dilkes D, Sues H-D (2009) Redescription and phylogenetic relationships of *Doswellia* *kaltenbachi* (Diapsida: Archosauriformes) from the Upper Triassic of Virginia. J Vert Paleontol 29: 58–79.

Evans SE (1982) Gliding reptiles of the Upper Permian. Zool J Linn Soc 76: 97–123.

Evans SE, Wang Y (2005) *Dalinghosaurus*, a lizard from the Early Cretaceous Jehol Biota of northeast China. Acta Palaeontol Polonica 50: 725–742

Fraser NC (1982) A new rhynchocephalian from the British Upper Triassic. Palaeontology 25: 709–725.

Galton PM (1990) Basal Sauropodomorpha-Prosauropoda. In: Weishampel DB, Dodson P, Osmólska H, editors. The Dinosauria. Bekerley: University of California Press. pp. 320-344.

Gregory JT (1945) Osteology and relationships of *Trilophosaurus*. University of Texas 4401: 273–359.

Ji S-A, Ji Q (2004) Postcranial anatomy of the Mesozoic *Dalinghosaurus* (Squamata): evidence from a new specimen of Western Liaoning. Acta Geologica Sinica 78: 897–906

Lee MSY (1998) Convergent evolution and character correlation in burrowing reptiles: towards a resolution of squamate relationships. Biol J Linn Soc 65: 369–453.

Nicholls EL (1999) A reexamination of *Thalattosaurus* and *Nectosaurus* and the relationships of the Thalattosauria (Reptilia: Diapsida). PaleoBios 19: 1–29.

Novas FE (1996) Dinosaur monophyly. J Vertebr Paleontol 16: 723–741.

Parks P (1969) Cranial anatomy and mastication of the Triassic reptile *Trilophosaurus*. MS Thesis. Austin: University of Texas. 100 p.

Parrish JM (1993) Phylogeny of the Crocodylotarsi, with reference to archosaurian and crurotarsan monophyly. J Vertebr Paleontol 13: 287–308.

Reisz RR, Godfrey S, Scott D (2009) *Eothyris* and *Oedaleops*: Do these early Permian synapsids form a clade? J Vertebr Paleontol 29: 39–47.

Reynoso VH (1998) *Huehuecuetzpalli mixtecus* gen. et sp. nov: a basal squamate (Reptilia) from the Early Cretaceous of Tepexi de Rodríguez, Central México. Philos Trans R Soc Lond B 353: 477–500.

Rieppel O, Mazin M, Tchernov E (1999) Sauropterygia from the Middle Triassic of Makhtesh Ramon, Negev, Israel. Fieldiana 40: 1**–**85.

Schaumberg GS, Unwin DM, Brandt S (2007) New information on the anatomy of the Late Permian gliding reptile *Coelurosauravus*. Paläontol Z 81: 160–173.

Sereno PC (1999) The evolution of dinosaurs. Science 284: 2137–2147.

Smith RMH, Evans SE (1996) New material of *Youngina*: evidence of juvenile aggregation in Permian diapsid reptiles. Palaeontology 39: 289–303.
